# Novel Oxime-Derivatized Synthetic Triterpene Glycosides as Potent Saponin Vaccine Adjuvants

**DOI:** 10.3389/fimmu.2022.865507

**Published:** 2022-05-06

**Authors:** Roberto Fuentes, Leire Aguinagalde, Carlo Pifferi, Adrián Plata, Nagore Sacristán, Donatello Castellana, Juan Anguita, Alberto Fernández-Tejada

**Affiliations:** ^1^ Chemical Immunology Laboratory, Center for Cooperative Research in Biosciences (CIC bioGUNE), Basque Research and Technology Alliance BRTA, Derio, Spain; ^2^ Research and Development, Center for Cooperative Research in Biosciences (CIC bioGUNE), Basque Research and Technology Alliance BRTA, Derio, Spain; ^3^ Inflammation and Macrophage Plasticity Laboratory, Center for Cooperative Research in Biosciences (CIC bioGUNE), Basque Research and Technology Alliance BRTA, Derio, Spain; ^4^ Ikerbasque, Basque Foundation for Science, Bilbao, Spain

**Keywords:** vaccine adjuvants, synthetic saponins, carbohydrates, immune responses, immunological evaluation

## Abstract

Vaccine adjuvants are key for optimal vaccine efficacy, increasing the immunogenicity of the antigen and potentiating the immune response. Saponin adjuvants such as the carbohydrate-based QS-21 natural product are among the most promising candidates in vaccine formulations, but suffer from inherent drawbacks that have hampered their use and approval as stand-alone adjuvants. Despite the recent development of synthetic derivatives with improved properties, their full potential has not yet been reached, allowing the prospect of discovering further optimized saponin variants with higher potency. Herein, we have designed, chemically synthesized, and immunologically evaluated novel oxime-derivatized saponin adjuvants with targeted structural modifications at key triterpene functionalities. The resulting analogues have revealed important findings into saponin structure-activity relationships, including adjuvant mechanistic insights, and have shown superior adjuvant activity in terms of significantly increased antibody response augmentation compared to our previous saponin leads. These newly identified saponin oximes emerge as highly promising synthetic adjuvants for further preclinical development towards potential next generation immunotherapeutics for future vaccine applications.

## Introduction

**Figure 1:**
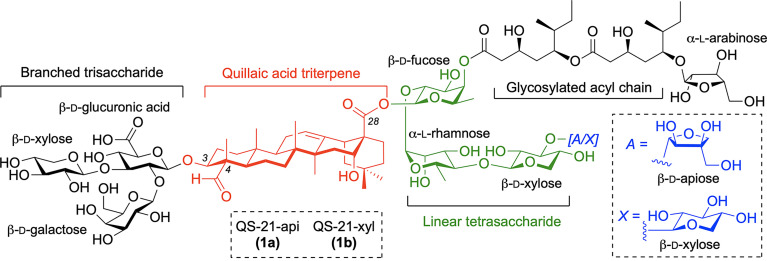
Structure of QS-21 saponin natural product adjuvant (**1a**/**1b**) with its four domains.

Current vaccine formulations based on well-defined antigenic substructures displayed in pathogens and cancer cells show low immunogenicity and require coadministration with an immunological adjuvant for optimal efficacy (1–3). Inclusion of the adjuvant increases the immunogenicity of these subunit vaccines, boosting the immune response (4). As such, appropriate selection of this key component is critically important for the clinical success of modern vaccines against infectious diseases and cancer. Among the most potent molecular adjuvants is QS-21, a saponin natural product extracted from the bark of the South American tree *Quillaja Saponaria* Molina (5, 6), that activates both humoral and cellular immunity, as demonstrated in several anticancer and antiviral vaccine trials (7, 8, 9). The structure of QS-21 is that of a complex triterpenoid glycoside incorporating a central quillaic acid (QA) triterpene (characterized by its C4-aldehyde and C16-hydroxyl group), which is flanked by a branched trisaccharide attached at its C3-hydroxyl and by a linear tetrasaccharide linked to its C28-carboxylic acid (**Figure 1**). This right-hand carbohydrate domain features either a terminal apiose (QS-21-api, **1a**) or xylose (QS-21-xyl, **1b**) unit, giving rise to a ~2:1 mixture of isomeric constituents, and is further functionalized at the 4-position of the fucose residue with a glycosylated diester acyl chain. QS-21 has been widely applied in a variety of infectious and antitumor vaccine clinical trials including two recent approvals in combination with monophosphoryl lipid A (MPLA) as part of the liposome-based AS01 adjuvant system in malaria (10) and shingles (11) vaccine formulations. However, the natural product QS-21 suffers from important liabilities that have impeded its advancement as a stand-alone adjuvant in vaccines, most notably limited availability (5) and lack of homogeneity from the natural source (12), structural instability (13), and toxic side effects (14). Reformulations of QS-21 with certain excipients and additives (co-polymers, lipids, etc.) have helped to increase its stability, reduce the associated pain, and improve acceptability (15), as best exemplified by the MPLA-containing liposomal coformulation in AS01 (16). Nonetheless, the AS01-adjuvanted shingles vaccine can cause short-term adverse events more intense than other vaccines, with about 1 out of 6 individuals experiencing side effects that prevented them from doing regular activities 2-3 days after injection (17). In addition, the molecular mechanism of action of QS-21 is not fully understood (18), with a proposed hypothesis suggesting a role for the quillaic acid (QA) C4-aldehyde substituent in adjuvant activity by engaging T cell receptors *via* Schiff-base formation (19), analogously to the structurally unrelated, aldehyde-containing, small-molecule immunopotentiator tucaresol (20, 21). The importance of the aldehyde was suggested based on the diminished activity of modified QS-21 saponins *via* condensation of the triterpene carbonyl group with exogenous ethylendiamine or glycine (22). However, the resulting derivatives obtained in these early studies were not fully characterized structurally, and not only lacked the aldehyde (as intended) but also altered the molecule net charge, which could be the origin of its attenuated adjuvant activity.

**Figure 2:**
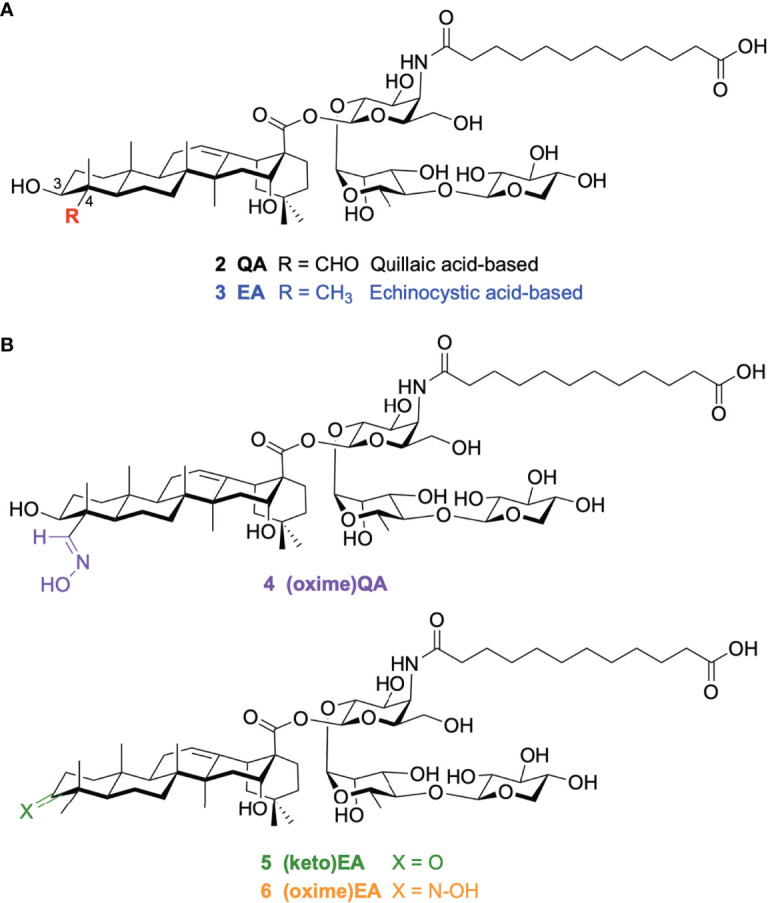
Chemical structure of **(A)** our previous quillaic acid and echinocystic acid-based saponin lead compounds: **2** QA and **3** EA; and **(B)** our novel saponin variants **4–6** discovered in this work.

To address the inherent challenges of the natural product in terms of manufacturing and dose-limiting tolerability, we have developed increasingly streamlined, less toxic QS-21 variants (23–28), that have provided critical insights into structure–activity relationships, while seeking improved, practical alternatives to QS-21 for adjuvant development. First, the complex, ester-containing labile side chain was shown to be replaceable with an aliphatic, amide-stabilized surrogate (29). Next, the terminal apiose/xylose residue within the linear oligosaccharide (30) and the whole left-hand carbohydrate domain proved to be dispensable for adjuvant activity (31) as assessed by antibody response augmentation, ultimately providing the most streamlined saponin variant 2 (**Figure 2**) (32). Modification of the triterpene C4 and C16 positions in simplified QS-21 synthetic derivatives based on alternative scaffolds led to potent saponin variants derived from the echinocystic acid (EA) core (e.g. **3**), which incorporated a C4-methyl group instead of the original C4-aldehyde substituent (32). While these new analogues induced high IgG antibody levels, they were below those elicited by QS-21, suggesting the prospect of discovering new saponin variants with potentially improved adjuvant activity through alternative structural variations within the triterpene region. In considering additional chemical modifications in this domain, we focused on the C4-aldehyde substituent and a previously unexplored site in the triterpene core (the C3-OH position originally glycosylated with the dispensable branched trisaccharide), which allowed us to explore further the importance of a triterpene carbonyl group for activity using homogeneous synthetic compounds. Herein, we report a new series of saponin variants (**4–6**) with 3-keto and 3/4-oxime modifications at the corresponding triterpene positions (**Figure 2**) that elicited potent antibody responses in mice. In the case of the oximated analogues, the design was inspired by previous (33) and ongoing unpublished studies in our group (34). Showing the positive impact of oxime linkages in the immunological activity of synthetic vaccine constructs. Notably, the oxime derivatives **4** and **6** were superior to saponin adjuvants **2** and **3**, (**Figure 2**) our previously identified lead compounds that induced antibody titers close (although lower) to those of the natural product adjuvant QS-21.

## Results and Discussion

### Synthesis of Saponin Triterpene Variants

**Scheme 1:**
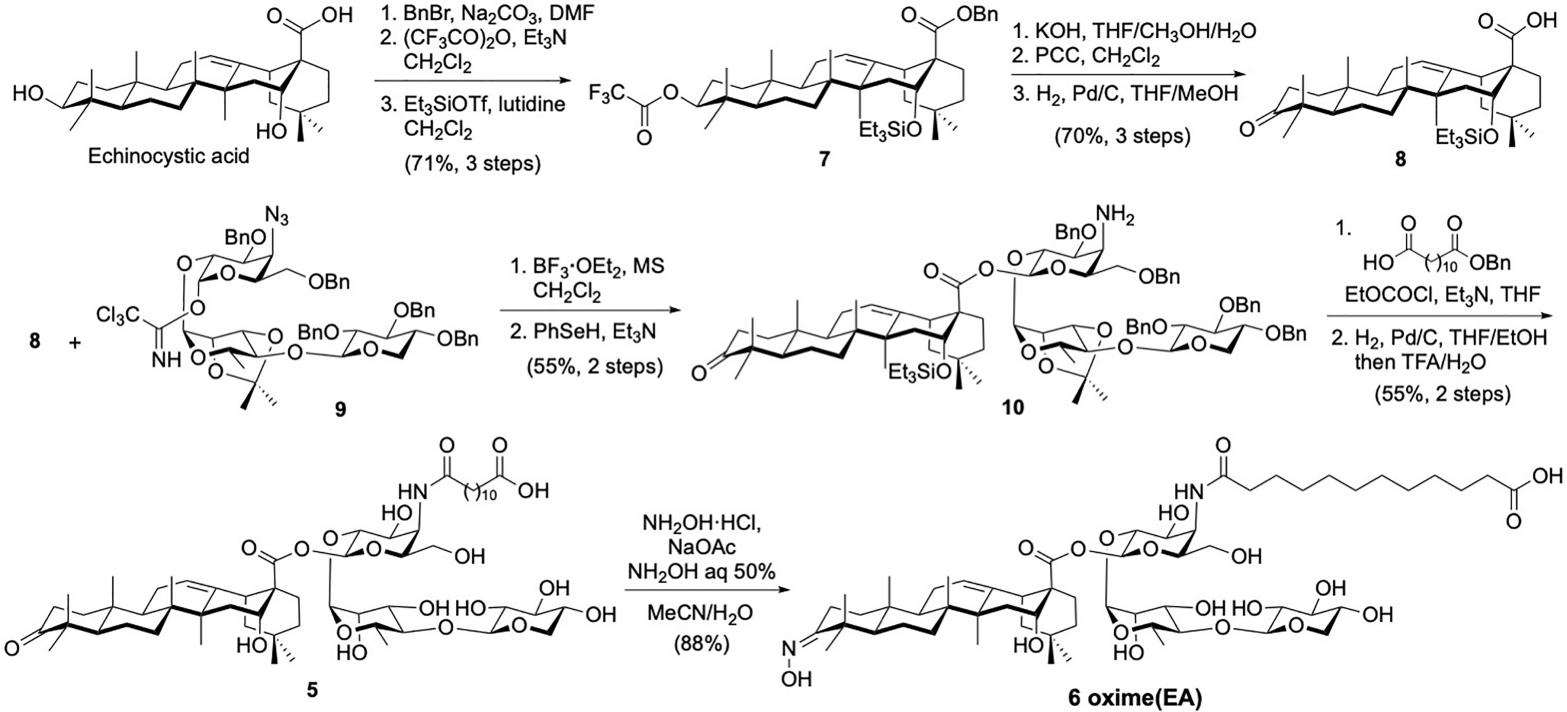
Chemical synthesis of echinocystic acid-based saponin adjuvants: C3-keto variant **5** and ketoxime saponin **6**.

Building upon our saponin leads **2** and **3**, we prepared three novel variants derived from quillaic acid and echinocystic acid, respectively. To synthesize saponin analogue **4**, late-stage oximation of quillaic acid variant **2** for 4 h at 45°C using a combination of hydroxylamine hydrochloride/sodium acetate in a mixture of acetonitrile/water and additional aqueous hydroxylamine (35, 36), provided the desired saponin aldoxime **4** (92% yield) after reverse-phase HPLC purification. In the case of the echinocystic acid derivatives, we followed a different approach involving initial preparation of a suitably protected triterpene having an oxidized C3-OH position as a ketone functionality *via* appropriate protecting group manipulation and oxidation reactions (**Scheme 1**). Briefly, commercially available echinocystic acid was selectively benzylated (benzyl bromide, sodium carbonate) and its C3- and C16-hydroxyl groups were orthogonally protected as a trifluoroacetate (trifluoroacetic anhydride, triethylamine) and a silyl ether (triethylsilyl trifluoromethanesulfonate (TESOTf), 2,6-lutidine), respectively, giving intermediate **7** (37). Selective deprotection of the C3-OH and subsequent oxidation with pyridinium chlorochromate (PCC), followed by removal of the benzyl ester afforded the desired C3-keto echinocystic acid scaffold (kEA, **8**). This oxidized triterpene was glycosylated with trisaccharide imidate donor **9** under Schmidt coupling conditions (BF_3_·OEt_2_) (32) to afford the corresponding glycoconjugate intermediate **10** after benzeneselenol (PhSeH) reduction of the azide. The remaining steps entailed acylation of the resulting amine with dodecanedioic acid monobenzyl ester followed by global deprotection *via* hydrogenolysis (H_2_, Pd/C) and acid hydrolysis (TFA/H_2_O) to provide, after HPLC purification, the novel C3-keto EA variant **5** (**Scheme 1**). Following analogous reaction conditions to those used for the synthesis of (oxime) QA **4**, we next derivatized the ketone moiety in **5** *via* oximation to prepare saponin ketoxime **6** in 88% yield, allowing us to investigate the effect of the C3 carbonyl group for adjuvant activity.

### Immunological Evaluation of Saponin Variants

**Figure 3:**
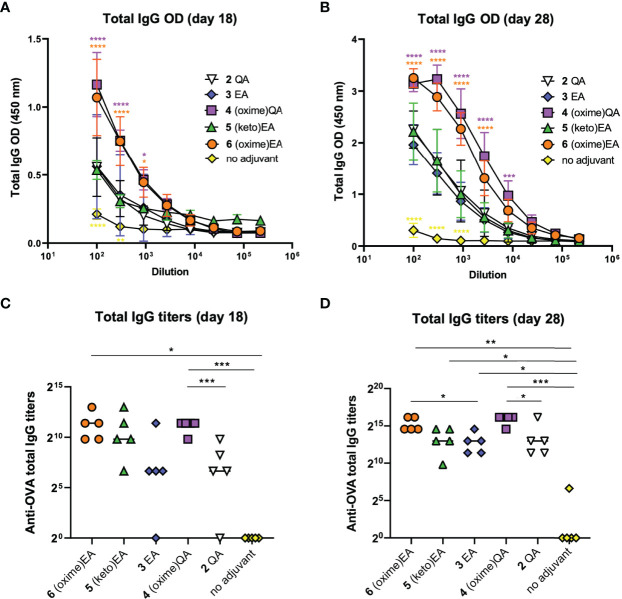
Antibody responses in mice induced by previous lead compounds **2** and **3** and novel saponin adjuvants **4**–**6** coadministered with OVA antigen. **(A, B)** ELISA standard curves showing total anti-OVA IgG levels (represented as optical density [OD] measurements) in mouse sera on **(A)** day 18 and **(B)** day 28 after first immunization. Sera were measured using 3-fold serial dilutions starting at 1/100 serum dilution. Statistical significance across the different dose-response curves was assessed by comparing to saponin lead **3** EA group using two-way ANOVA Dunnett’s multiple comparisons test at the various dilutions. *p ≤ 0.05, **p ≤ 0.01, ***p ≤ 0.001, ****p ≤ 0.0001. **(C, D)** Anti-OVA total IgG titers on **(C)** day 18 and **(D)** day 28. Data points correspond to individual mice (five animals per group) and horizontal bars indicate median titers. Statistical significance was assessed by (1) comparing each group to the no-adjuvant (OVA alone) control and (2) comparing each new saponin variant to its corresponding parent lead (e.g. **4** versus **2** QA; **5**, **6** versus **3** EA) using a two-tailed unpaired Student’s *t*-test with a 95% confidence interval (CI) *p ≤ 0.05, **p ≤ 0.01, ***p ≤ 0.001.

With these three new saponin analogues in hand [**4–6** (**Figure 2**)], we evaluated their adjuvant activity in a mouse vaccination model involving coadministration with ovalbumin (OVA) as an immunogen. Groups of five mice (female C57BL/6, 6–8 weeks of age) were immunized with endotoxin-free OVA (EndoFit™ Ovalbumin; Invitrogen) (10 μg) and the saponin of interest (50 μg) in phosphate-buffered saline (PBS, 100 μl) *via* subcutaneous injections on days 0 and 11, followed by a booster on day 21. As a key goal of this study was to improve upon our previous saponin candidates, for comparison, positive control groups included mice vaccinated with the parent quillaic acid and echinocystic acid lead adjuvants **2** and **3**, (**Figure 2**) which induced the closest antibody responses to QS-21 among our synthetic variants (32). As negative control, another group was administered the OVA antigen only (no-adjuvant group). Mouse blood was collected three days before (day 18) and one week after the booster (third) immunization, at the time of sacrifice (day 28), and the presence of antibodies against OVA in sera was detected by ELISA at both time points (**Figures 3A, B**). Anti-OVA antibody titers were calculated following ELISA analysis at days 18 and 28 after the first immunization, according to the previously reported method (31) (**Figures 3C, D**).

On day 18, aldoxime **4** and ketoxime saponin **6** elicited significantly higher anti-OVA IgG levels than those of the EA lead variant **3**, which in turn were also significantly increased compared to those of the OVA-alone (no adjuvant) negative control (**Figure 3A**). In terms of titers, (oxime)QA **4** induced significantly higher IgG antibodies than the corresponding QA saponin 2 (**Figure 3C**). The IgG titers elicited by the new EA derivatives, (keto)EA **5** and (oxime)EA **6**, were considerably increased relative to those of the parent EA variant **3**, albeit there was no significant difference in these cases. When compared to the no-adjuvant control group, both oxime saponins **4** and **6** induced significantly higher titers than mice immunized with OVA alone (**Figure 3C**). As expected, IgG antibody levels augmented on day 28 upon the booster, with all variants eliciting further elevated humoral responses (**Figure 3B**). Remarkably, while mice administered the C3-keto EA analogue **5** showed comparable antibody levels to those of the positive control groups immunized with compounds **2** and **3**, the levels induced by saponin oximes **4** and **6** were even significantly higher than those observed in the presence of our previous lead variant **3** EA (**Figure 3B**). This trend was maintained when looking at antibody titers, with all three novel saponins showing significantly higher IgG antibodies than the no-adjuvant control group, and both oxime derivatives **4** and **6** inducing significantly increased titers compared to those of their respective QA and EA analogues **2** and **3** (**Figure 3D**). Given the comparable antibody response augmentation previously shown by these parent variants (2 QA and **3** EA) relative to QS-21 (32), it can be expected that these newly developed saponin oxime adjuvants (**4** and **6**) could induce antibody titers generally rivalling those of QS-21, a prospect that we are testing in further comparative studies.

**Figure 4:**
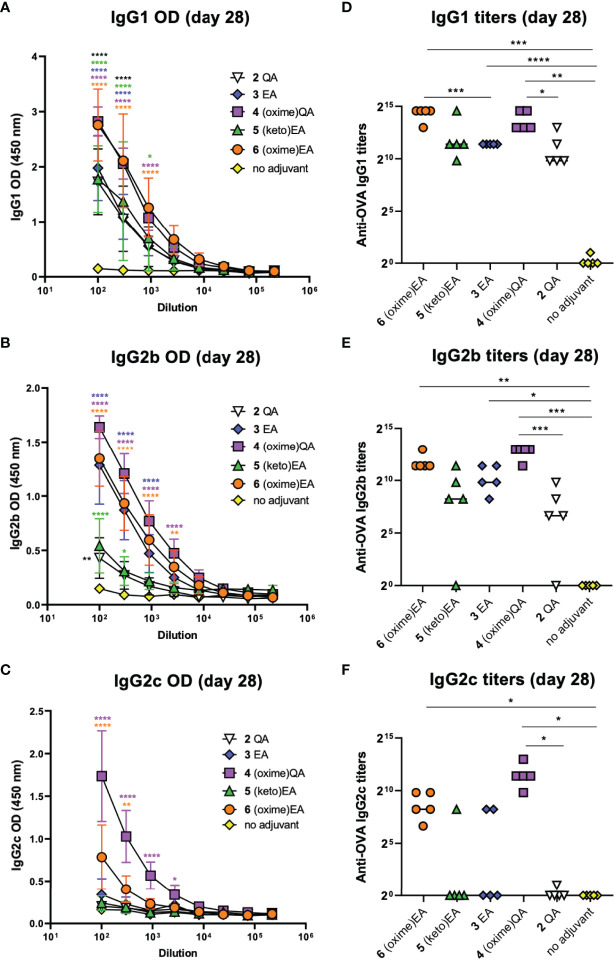
IgG subtyping of anti-OVA antibodies at day 28 resulting from mouse vaccinations with previous lead compounds **2** and **3** and novel saponin adjuvants **4**–**6** coadministered with OVA antigen. **(A–C)** ELISA standard curves showing anti-OVA IgG subtype levels (represented as optical density [OD] measurements) of **(A)** IgG1, **(B)** IgG2b, and **(C)** IgG2c antibodies on day 28 after first immunization. Sera were measured using 3-fold serial dilutions starting at 1/100 serum dilution. Statistical significance across the different dose-response curves was assessed by comparing to the no-adjuvant (OVA alone) control group using two-way ANOVA Dunnett’s multiple comparisons test at the various dilutions. *p ≤ 0.05, **p ≤ 0.01, ***p ≤ 0.001, ****p ≤ 0.0001. **(D–F)** Anti-OVA IgG subtype titers of **(D)** IgG1, **(E)** IgG2b, and **(F)** IgG2c antibodies on day 28. Data points correspond to individual mice (five animals per group) and horizontal bars indicate median titers. Statistical significance was assessed by (1) comparing each group to the no-adjuvant negative control and (2) comparing each new saponin variant to its corresponding parent lead (e.g. **4** versus **2** QA; **5**, **6** versus **3** EA) using a two-tailed unpaired Student’s *t*-test with a 95% confidence interval (CI) *p ≤ 0.05, **p ≤ 0.01, ***p ≤ 0.001, ****p ≤ 0.0001.

IgG subtyping of the anti-OVA antibodies (day 28) elicited by the saponin variants showed a similar trend for the IgG1 subclass (**Figures 4A, D**) to that observed for total IgG (**Figures 3B, D**). Both oxime-modified saponins (**4** and **6**) induced increased IgG1 antibodies than the other adjuvant-active variants: **2**, **3**, and the novel EA derivative **5**. Yet, this C3-keto saponin **5** generated considerably higher IgG1 levels than the negative control group (**Figures 4A, D**). In terms of IgG2 isotypes, the QA-derived oxime analogue **4** showed the highest antibody levels for both the IgG2b (**Figures 4B, E**) and IgG2c (**Figures 4C, F**) subclass, with titers significantly higher than the OVA-alone (no adjuvant) group and even than the positive control **2** QA (**Figures 4E, F**). The corresponding EA-based variants, oxime **6**, ketone **5**, and previous lead 3 elicited generally lower IgG2 responses, standing out ketoxime **6** among them (**Figures 4E, F**). Notably, the differences in antibody subclass levels were greater in the case of the IgG2c subtype, with both oxime saponins **4** and **6** inducing significantly higher titers than the no-adjuvant control (**Figure 4F**). Because IgG2a (IgG2c in C57BL/6 mice) production is associated to Th1 immunity (38) and is increased by Th1 adjuvants, like QS-21, the high levels of this subclass induced by the saponin oximes **4** and **6** suggest that these two oxime-functionalized variants may elicit a Th1-biased response, as is the case of natural QS-21. However, despite having an oxo group, the C3-keto saponin **5** elicited IgG1 rather than IgG2c antibodies, suggestive of Th2-like immunity, in contrast to other Th1 triterpene saponin adjuvants having imine-forming carbonyl functionalities (39).

Moreover, initial general assessment of saponin toxicity by monitoring mouse weight change at 0 h, 24 h and 48 h post-injection revealed negligible mouse weight loss (**Supplementary Figure S3**), as opposed to mice receiving QS-21 (20 µg dose), which as seen in several previous studies lost around 10% of their body mass on average (not shown). This together with the absence of signs of toxic side effects after vaccination indicates a good overall tolerability of these new saponin adjuvants, albeit further comprehensive toxicology studies are warranted to provide additional, detailed information on this front, especially in comparison to the known reactogenicity and dose-limiting toxicity of natural QS-21 alone.

## Conclusion

In conclusion, the superior antibody production elicited by these new oxime-containing variants compared to our previous lead compounds is of note, as it signals the discovery of two novel saponin adjuvants with further enhanced adjuvant potency in terms of significantly increased antibody response augmentation than that achieved by our established saponin leads. It will also be of interest to assess the *in vivo* effect of these novel adjuvants in antigen-presenting cell activation (*e.g.* co-stimulatory molecule expression), cytokine production and T-cell responses, as well as their adjuvant activity relative to QS-21 as a benchmark. Moreover, the fact that carbonyl-containing derivative **5** generates similar antibody levels than its echinocystic acid parent compound (**3**), but lower than the corresponding modified analogue in which the C3 ketone has been derivatized as an oxime (**6**), highlights the non-necessity of the triterpene carbonyl group for inducing potent humoral immunity by these saponin variants, casting doubt on the hypothesized key role of the C4-aldehyde substituent of QS-21 in adjuvant activity (19). Indeed, other triterpene glycoside saponins devoid of carbonyl groups have been reported to possess adjuvant activity (40, 41), suggesting the existence of additional mechanisms of action not directly involving T-cell interaction and co-stimulation (39). Overall, it is unlikely that a unique, general mechanism of action can be applicable to the wide variety of saponin adjuvant families that exist, some of them lacking imine-forming aldehyde/ketone functionalities (18). Notably, our data also reveal a previously unknown positive effect of triterpene oxime functionalization in enhancing antibody responses in the context of these streamlined saponins. In all, these newly identified oxime derivatives emerge as optimal synthetic alternatives for additional preclinical and mechanistic studies using well-known adjuvant controls (*i.e.*, QS-21 and AS01-like formulations) as well as for further development as promising immunopotentiators in new vaccine applications.

## Materials and Methods

### General Information

#### General Synthetic Methods

All commercially available materials were used without further purification except boron trifluoride diethyl etherate (BF_3_·Et_2_O) and trifluoromethanesulfonic anhydride (Tf_2_O), which were distilled from calcium hydride and phosphorus pentoxide, respectively, at 1 atm under N_2_. All manipulations with air-sensitive reagents and chemical reactions were carried out under a dry argon atmosphere using standard Schlenk techniques. Air- and moisture-sensitive liquids and solutions were transferred *via* syringe. The appropriate carbohydrate reagents were dried *via* azeotropic removal of water with toluene. Molecular sieves were activated at 350°C and were crushed immediately prior to use, then dried under vacuum. Organic solutions were concentrated under reduced pressure by rotary evaporation below 40°C. Column chromatography was performed employing 230–400 mesh silica gel. Analytical thin-layer chromatography (TLC) was performed using aluminum-backed sheets pre-coated with 230–400 mesh silica gel 60 containing fluorescent indicator (F254). Preparative TLC (Analtech Uniplates) was performed using glass-backed sheets pre-coated with 500-micron silica gel containing fluorescent indicator (F254). TLC plates were visualized under UV light (254 nm) and by staining with cerium ammonium molybdate (CAM) or 5% sulfuric acid in ethanol solutions.

#### Nuclear Magnetic Resonance (NMR)

^1^H, APT ^13^C, COSY and HSQC spectra were recorded on a Bruker Avance III instrument (^1^H NMR at 600 MHz and APT ^13^C NMR at 151 MHz) and Bruker AVANCE NEO spectrometer (^1^H NMR at 400 MHz and APT ^13^C NMR at 101 MHz), equipped with a SmartProbe and operating under TopSpin 4.1.1. Chemical shifts are expressed in parts per million (δ scale) downfield from tetramethylsilane and are referenced to residual proton in the NMR solvent (CDCl_3_: δ 7.26 for ^1^H NMR, δ 77.00 for ^13^C NMR; methanol-*d*_4_: δ 3.31 for ^1^H NMR, δ 49.15 for ^13^C NMR). Data are presented as follows: chemical shift, multiplicity (s = singlet, br s = broad singlet, d = doublet, t = triplet, q = quartet, m = multiplet and/or multiple resonances), coupling constant (J) in Hertz (Hz), integration, assignment.

#### RP-HPLC Purification and LC-MS

All reverse-phase RP-HPLC analyses/purifications were carried out on a Waters 1525 binary gradient system (Solv. A = 0.05% TFA in H_2_O; Solv. B = 0.05% TFA in CH_3_CN) equipped with a Waters 2998 photodiode array detector (PDA), and combined with a low-resolution single quadrupole (SQD2, Waters Corporation) mass spectrometer. Absorbances were monitored at wavelengths of 190–600 nm.

#### HR-ESI-MS

High resolution electrospray ionization mass spectrometry (HR-ESI-MS) was performed on a Waters LCT Premier XE (Waters, Milford, MA, USA) in W-optics positive ionization scan mode. Mass spectrometry parameters were optimized to achieve the best signal-to-noise ratio: capillary voltage 1 kV, sample cone voltage 100 V, desolvation gas flow 600 Lh^-1^, cone gas flow 50 Lh^-1^, desolvation temperature 350°C, source temperature 150°C. The instrument was calibrated over the range *m/z* 200-2000 before measurement using a standard NaI solution (1 µM). In order to minimize the accuracy in the measurements, Leucine-Enkephalin was used as a lockmass reference [2M+Na], *m/z* 1111.5459. Data analysis was performed with Masslynx software version 4.1 (Waters, Milford, MA, USA). Characterization by MS was corroborated after comparing the experimental isotopic pattern with the theoretical one.

#### MALDI-TOF-HR-MS

High resolution MALDI-TOF mass spectra analyses were performed on an UltrafleXtreme III MALDI-time-of-flight (TOF) mass spectrometer equipped with a pulsed Nd : YAG laser (355 nm) and controlled by FlexControl 3.3 software (Bruker Daltonics, Bremen, Germany). The acquisitions were carried out in positive reflector ion mode with pulse duration of 50 ns. Laser intensity was set marginally above the threshold of ionization to avoid fragmentation. The *m/z* range was chosen according to the mass of the sample. The acquired data was processed using the mMass software.

### Chemical Synthesis

#### Synthesis of (Oxime) Quillaic Acid Saponin (4)

A solution of NH_2_OH·HCl (5.76 mg, 0.08 mmol, 21.3 equiv) and NaOAc (10.26 mg, 0.13 mmol, 32.2 equiv) in acetonitrile/water (3:1, 2.4 mL) was added to quillaic acid saponin **2** (4.41 mg, 3.9 μmol, 1.0 equiv). To this mixture, an excess (100 μL) of NH_2_OH 50% aq. soln. was added and the reaction suspension was left stirring overnight at 45°C. The crude mixture was directly purified *via* RP-HPLC (< 0.3 mL per injection) on a XBridge Prep BEH300 C18 column (5 μm, 19 × 150 mm) using a linear gradient of 30–64% acetonitrile/water containing 0.05% trifluoroacetic acid over 12 min (after initial 5 min at starting conditions) at a flow rate of 17 mL/min. The fraction containing the major peak (t_R_ = 15.81 min) was collected and lyophilized to dryness to provide the desired (oxime)QA saponin **4** (4.12 mg, 92% yield) as a white foam.

HPLC: t_R_ = 25.49 min (gradient = 30-100% acetonitrile/water over 30 min), λ_max_ = 193.52 nm. ^1^H NMR (400 MHz, methanol-*d*_4_): *δ* 7.13 (s, 1H, C*H*NOH), 5.37 (d, *J* = 1.8 Hz, 1H, H-1 Rha), 5.34 (d, *J* = 7.2 Hz, 1H, H-1 N-Gal), 5.30 (t, *J* = 3.6 Hz, 1H, H-12 QA), 4.51 – 4.47 (m, 2H, H-1 Xyl, H-16 QA), 4.34 – 4.31 (m, 1H, H-4 N-Gal), 3.97 – 3.90 (m, 3H, H-3 & H-2 N-Gal, H-2 Rha), 3.89 – 3.76 (m, 3H, H-5a Xyl, H-5 & H-3 Rha), 3.69 (td, *J* = 6.6, 1.7 Hz, 1H, H-5 N-Gal), 3.59 – 3.46 (m, 4H, H-4 Rha, H-3 QA, H-4 Xyl, H-6a N-Gal), 3.45 – 3.38 (m, 1H, H-6b N-Gal), 3.36 – 3.11 (m, 3H, H-3 & H-2 Xyl, H-5b Xyl), 2.94 (dd, *J* = 14.3, 4.5 Hz, 1H, H-18 QA), 2.41 – 2.22 (m, 5H, H-19a QA, C*H*_2_(a)CONH & C*H*_2_(a’)CO_2_H acyl), 2.01 – 1.87 (m, 4H, H-11a,b, H-22a & H-21a QA), 1.87 – 1.74 (m, 1H, H-22b QA), 1.74 – 1.56 (m, 9H, H-2a,b, H-9, H-1a & H-15a QA, C*H*_2(b)_CH_2_CONH & C*H*_2(b’)_CH_2_CO_2_H acyl), 1.55 – 1.40 (m, 3H, H-7a, H-15b & H-6a QA), 1.39 – 1.24 [m, 20H, (1.39 s, 3H, CH_3_ C-27 QA), H-7b & H-6b QA, CH_3_ Rha, 6 × C*H*_2(c)_ internal acyl], 1.22 – 1.13 (m, 1H, H-21 QA), 1.12 – 0.98 [m, 9H, (1.03 s, 3H, CH_3_ C-24 QA), (1.01 s, 3H, CH_3_ C-25 QA), H-1b, H-19b & H-5 QA], 0.96 (s, 3H, CH_3_ C-30 QA), 0.88 (s, 3H, CH_3_ C-29 QA), 0.77 (s, 3H, CH_3_ C-26 QA). ^13^C NMR (101 MHz, methanol-*d*_4_): *δ* 178.5 (CONH acyl), 177.8 (CO_2_H acyl), 177.1 [CO (C-28) QA], 161.0 (CNOH), 144.8 (C-13 QA), 123.3 (C-12 QA), 107.0 (C-1 Xyl), 101.4 (C-1 Rha), 95.5 (C-1 N-Gal), 84.2 (C-4 Rha), 78.2 (C-3 Xyl), 76.5 (C-3 QA), 76.3 (C-5 N-Gal), 76.1 (C-2 Xyl), 74.8 (C-3 & C-2 N-Gal), 74.7 (C-16 QA), 72.2 (C-3 Rha), 71.9 (C-2 Rha), 71.0 (C-4 Xyl), 68.9 (C-5 Rha), 67.3 (C-5 Xyl), 61.7 (C-6 N-Gal), 52.9 (C-5 QA), 52.5 (C-4 N-Gal), 50.0 (C-17 QA), 48.2 (C-9 QA), 48.0 (C-19 QA), 47.4 (C-4 QA), 42.7 (C-14 QA), 42.3 (C-18 QA), 41.1 (C-8 QA), 39.7 (C-1 QA), 37.7 (C-10 QA), 36.8 (*C*H_2(a)_CONH acyl), 36.5 (C-15 & C-21 QA), 35.1 (*C*H_2(a’)_CO_2_H acyl QA), 33.8 (C-7 QA), 33.4 (CH_3_ C-29), 32.0 (C-22 QA), 31.3 (C-20 QA), 30.6, 30.5, 30.4, 30.34, 30.26 (6 × CH_2(c)_ internal acyl), 27.23 (*C*H_2(b)_CH_2_CONH acyl), 27.21 (CH_3_ C-27 QA), 27.0 (C-2 QA), 26.2 (*C*H_2(b’)_CO_2_H acyl), 24.9 (CH_3_ C-30 QA), 24.5 (C-11 QA), 20.9 (C-6 QA), 18.4 (CH_3_ Rha), 17.8 (CH_3_ C-26 QA), 16.6 (CH_3_ C-25 QA), 12.0 (CH_3_ C-24 QA). HRMS (ESI^–^) *m/z*: Calcd for [C_59_H_95_N_2_O_20_]**^–^** [M–H] **^–^** 1151.6476, found: 1151.6483.

#### Synthesis of (Keto) Echinocystic Acid Saponin (5)

To fully protected (keto)echinocystic acid carboxyacyl saponin **S5** (36.6 mg, 0.020 mmol, 1.0 equiv) dissolved in tetrahydrofuran/ethanol (1:1, 24.5 mL), 10% (dry basis) Pd/C 50% wet Degussa type E101 NE/W (214 mg, 0.10 mmol, 5.0 equiv) was added. The reaction mixture was stirred at rt under H_2_ atmosphere (1 atm, balloon) for 3 h. Direct infusion (MS) confirmed the absence of starting material or intermediates. The suspension was filtered through 0.45 µm PTFE filter disk, washed extensively with methanol (3 × 10 mL) and concentrated to dryness. The crude was dissolved in a precooled (0°C) solution of trifluoroacetic acid (TFA/H_2_O 2:1, 4.4 mL), stirred at 0°C for 0.5 h, and the solvent was then evaporated to dryness *in vacuo*. The final residue was dissolved in a mixture of acetonitrile/water (0.05% TFA) 1:1 (3 mL), filtered through 0.2 µm PTFE filter disk, and purified by RP-HPLC (< 0.3 mL per injection) on a XBridge Prep BEH300 C18 column (5 μm, 19 × 150 mm) using a linear gradient of 30–65% acetonitrile/water (0.05% TFA) over 12.5 min (after initial 5 min at starting conditions) at a flow rate of 17 mL/min. The fraction containing the major peak (t_R_ = 17.01 min) was collected and lyophilized to dryness to afford the final (keto)EA saponin **5** (17.3 mg, 77% yield) as a white powder.

HPLC: t_R_ = 21.1 min (gradient = 20–100% solv. B over 30 min), λ_max_ = 194.52 nm. ^1^H NMR (400 MHz, methanol-*d*_4_): *δ* 5.40 (d, J = 1.8 Hz, 1H, H-1 Rha), 5.36 (d, J = 7.9 Hz, 1H, H-1 N-Gal), 5.33 (t, J = 3.3 Hz, 1H, H-12 EA), 4.52 – 4.47 (m, 2H, H-1 Xyl, H-16 EA), 4.35 – 4.31 (m, 1H, H-4 N-Gal), 3.98 – 3.92 (m, 3H, H-3 & H-2 N-Gal, H-2 Rha), 3.91 – 3.77 (m, 3H, H-5a Xyl, H-5 & H-3 Rha), 3.70 (td, J = 6.5, 1.6 Hz, 1H, H-5 N-Gal), 3.60 – 3.38 (m, 4H, H-4 Rha, H-4 Xyl, H-6a,b N-Gal), 3.36 – 3.32 (m, 1H, H-3 Xyl), 3.28 – 3.17 (m, 2H, H-2 Xyl, H-5b Xyl), 2.95 (dd, J = 14.4, 4.5 Hz, 1H, H-18 EA), 2.56 (ddd, J = 15.9, 10.3, 7.5 Hz, 1H, H-2a EA), 2.46 – 2.24 (m, 6H, H-2b & H-19a EA, C*H*_2_(a)CONH & C*H*_2_(a’)CO_2_H acyl), 2.02 – 1.88 (m, 5H, H-11a,b, H-22a, H-21a & H-1a EA), 1.86 – 1.67 (m, 3H, H-22b, H-9 & H-15a EA), 1.67 – 1.44 (m, 10H, C*H*_2(b)_CH_2_CONH & C*H*_2(b’)_CH_2_CO_2_H acyl, H-7a,b, H-6a,b, H-15b & H-1b), 1.42 – 1.30 [m, 19H, H-5 EA, (1.40 s, 3H, CH_3_ C-27 EA), (1.35 (d, *J* = 6.1 Hz, 3H, CH_3_ Rha)], 6 × C*H*_2(c)_ internal acyl), 1.18 (d, J = 12.2 Hz, 1H, H-21b EA), 1.11 – 1.03 [m, 10H, H-19b EA, (1.09 s, 3H, CH_3_ C-23 EA), (1.08 s, 3H, CH_3_ C-25 EA), (1.06 s, 3H, CH_3_ C-24 EA)], 0.96 (s, 3H, CH_3_ C-30 EA), 0.88 (s, 3H, CH_3_ C-29 EA), 0.83 (s, 3H, CH_3_ C-26 EA). ^13^C NMR (101 MHz, methanol-*d*_4_): *δ* 220.7 (C-3 EA), 178.5 (CONH acyl), 177.9 (CO_2_H acyl), 177.0 (CO [C-28] EA), 144.8 (C-13 EA), 123.3 (C-12 EA), 107.1 (C-1 Xyl), 101.3 (C-1 Rha), 95.5 (C-1 N-Gal), 84.3 (C-4 Rha), 78.2 (C-3 Xyl), 76.3 (C-5 N-Gal), 76.2 (C-2 Xyl), 74.9 (C-3 N-Gal), 74.6 (C-16 EA), 74.5 (C-2 N-Gal), 72.2 (C-3 Rha), 71.9 (C-2 Rha), 71.1 (C-4 Xyl), 68.9 (C-5 Rha), 67.3 (C-5 Xyl), 61.7 (C-6 N-Gal), 56.6 (C-5 EA), 52.5 (C-4 N-Gal), 50.1 (C-17 EA), 48.5 (C-4 EA), 48.0 (C-19 EA), 47.3 (C-9 EA), 42.9 (C-14 EA), 42.5 (C-18 EA), 40.7 (C-8 EA), 40.4 (C-1 EA), 37.9 (C-10 EA), 36.8 (*C*H_2(a)_CONH acyl), 36.5 (C-15 & C-21 EA), 35.1 (*C*H_2(a’)_CO_2_H acyl & C-2 EA), 33.7 (C-7 EA), 33.4 (CH_3_ C-29 EA), 32.0 (C-22 EA), 31.3 (C-20 EA), 30.6, 30.5, 30.4, 30.34, 30.26 (6 × CH_2(c)_ internal acyl), 27.22 (*C*H_2(b)_CH_2_CONH acyl), 27.15 (CH_3_ C-23 EA), 27.1 (CH_3_ C-27 EA), 26.2 (*C*H_2(b’)_CO_2_H acyl), 24.9 (CH_3_ C-30 EA), 24.6 (C-11 EA), 21.9 (CH_3_ C-24 EA), 20.9 (C-6 EA), 18.4 (CH_3_ Rha), 17.7 (CH_3_ C-26 EA), 15.8 (CH_3_ C-25 EA). HRMS (MALDI) *m/z*: Calcd for [C_59_H_95_NO_19_Na]^+^ [M+Na]^+^ 1144.6387, found 1144.6315.

#### Synthesis of (Oxime) Echinocystic Acid Saponin (6)

(Keto)echinocystic acid saponin **5** (3.51 mg, 3.1 μmol, 1.0 equiv) was dissolved in acetonitrile/water (3:1, 1.2 mL) containing NH_2_OH·HCl (2.94 mg, 42.3 μmol, 13.5 equiv) and NaOAc (5.16 mg, 62.8 μmol, 20.1 equiv). To this mixture, an excess (60 μL) of NH_2_OH aq. 50% was added and the reaction was left stirring overnight at 45 °C. RP-HPLC purification (<0.3 mL per injection) was then performed on a XBridge Prep BEH300 C18 column (5 μm, 19 × 150 mm) using a linear gradient of 35–48% acetonitrile/water over 5 min (after initial 5 min at starting conditions) at a flow rate of 17 mL/min. The fraction containing the major peak (t_R_ = 6.36 min) was collected and lyophilized to dryness to afford the final (oxime)EA saponin **6** (3.15 mg, 88% yield) as a white powder.

HPLC: t_R_ = 28.22 min (gradient = 35-100% solv. B over 30 min), λ_max_ = 194.52 nm. ^1^H NMR (400 MHz, methanol-*d*_4_): *δ* 5.39 (d, *J* = 1.8 Hz, 1H, H-1 Rha), 5.35 (d, *J* = 7.9 Hz, 1H, H-1 N-Gal), 5.32 (t, *J* = 3.7 Hz, 1H, H-12 EA), 4.52 – 4.47 (m, 2H, H-1 Xyl, H-16 EA), 4.35 – 4.30 (m, 1H, H-4 N-Gal), 3.97 – 3.90 (m, 3H, H-3 & H-2 N-Gal, H-2 Rha), 3.90 – 3.77 (m, 3H, H-5a Xyl, H-5 & H-3 Rha), 3.69 (td, *J* = 6.5, 1.7 Hz, 1H, H-5 N-Gal), 3.60 – 3.38 (m, 4H, H-4 Rha, H-4 Xyl, H-6a,b N-Gal), 3.37 – 3.33 (m, 1H, H-3 Xyl), 3.28 – 3.16 (m, 2H, H-2 & H-5b Xyl), 3.03 – 2.91 (m, 2H, H-2a & H-18 EA), 2.40 – 2.28 (m, 3H, H-19a EA, C*H*_2_(a)CONH acyl), 2.28 – 2.21 (m, 1H, H-2b EA), 2.19 – 2.10 (m, 2H, C*H*_2_(a’)CO_2_H acyl), 1.97 – 1.90 (m, 4H, H-11a,b, H-22a & H-21a EA), 1.85 – 1.73 (m, 2H, H-22b & H-1a EA), 1.73 – 1.55 (m, 7H, H-15a, H-9 & H-6a EA, C*H*_2(b)_CH_2_CONH & C*H*_2(b’)_CH_2_CO_2_H acyl), 1.54 – 1.41 (m, 4H, H-6b, H-7a,b & H-15b EA), 1.41 – 1.26 [m, 18H, (1.38 s, 3H, CH_3_ C-27 EA), CH_3_ Rha, 6 × C*H*_2(c)_ internal acyl], 1.22 – 1.12 [m, 4H, (1.15 s, 3H, CH_3_ C-23 EA), H-21b EA], 1.14 – 1.02 [m, 9H, (1.06 s, 6H, CH_3_ C-25 & CH_3_ C-24 EA), H-1b, H-19b & H-5 EA], 0.95 (s, 3H, CH_3_ C-30 EA), 0.88 (s, 3H, CH_3_ C-29 EA), 0.81 (s, 3H, CH_3_ C-26 EA). ^13^C NMR (101 MHz, methanol-*d*_4_): *δ* 183.1 (CO_2_H acyl), 178.5 (CONH acyl), 177.1 [CO (C-28) EA], 167.3 (CNOH), 144.7 (C-13 EA), 123.4 (C-12 EA), 107.1 (C-1 Xyl), 101.4 (C-1 Rha), 95.6 (C-1 N-Gal), 84.3 (C-4 Rha), 78.2 (C-3 Xyl), 76.4 (C-5 N-Gal), 76.2 (C-2 Xyl), 74.8 (C-3 N-Gal), 74.7 (C-16 EA), 74.6 (C-2 N-Gal), 72.2 (C-3 Rha), 71.9 (C-2 Rha), 71.1 (C-4 Xyl), 68.9 (C-5 Rha), 67.3 (C-5 Xyl), 61.7 (C-6 N-Gal), 57.3 (C-5 EA), 52.5 (C-4 N-Gal), 50.1 (C-17 EA), 48.0 (C-19 EA), 47.6 (C-9 EA), 42.8 (C-14 EA), 42.4 (C-18 EA), 41.0 (C-4 EA), 40.8 (C-8 EA), 39.7 (C-1 EA), 39.3 (*C*H_2(a)_CONH acyl), 38.2 (C-10 EA), 36.8 (*C*H_2(a’)_CO_2_H acyl), 36.5 (C-15 & C-21 EA), 34.0 (C-7 EA), 33.4 (CH_3_ C-29 EA), 32.0 (C-22 EA), 31.3 (C-20 EA), 30.9, 30.7, 30.6, 30.5, 30.4 (6 × CH_2(c)_ internal acyl), 28.2 (CH_3_ C-23 EA), 27.8 (*C*H_2(b’)_CO_2_H acyl), 27.3 (*C*H_2(b)_CH_2_CONH acyl), 27.1 (CH_3_ C-27 EA), 24.9 (CH_3_ C-30 EA), 24.6 (C-11 EA), 23.9 (CH_3_ C-24 EA), 20.4 (C-6 EA), 18.4 (CH_3_ Rha), 17.9 (C-2 EA), 17.8 (CH_3_ C-26 EA), 15.7 (CH_3_ C-25 EA). HRMS (MALDI) *m/z*: Calcd for [C_59_H_96_N_2_O_19_Na]^+^ [M+Na]^+^ 1159.6496, found: 1159.6463.

See **Supplementary Material** for additional protocols, detailed experimental procedures and complete characterization data for early triterpene and saponin synthetic intermediates.

### Immunological Evaluation

#### Vaccination of Mice

Groups of five mice (C57BL/6, female, 6-8 weeks old) were vaccinated subcutaneously three times every 10 days (days 0, 11, and 21) with endotoxin-free OVA (EndoFit™ Ovalbumin, Invitrogen) (10 μg/mouse) in phosphate-buffered saline (PBS, 100 μL) either alone (no-adjuvant control group) or with the synthetic saponins (50 μg/mouse). To analyze the antibody responses over time, mice were bled *via* the submandibular vein at the indicated pre- (day –1) and post-vaccination timepoint (day 18), and by cardiac puncture at the experimental endpoint (day 28). Blood was collected in BD Microtainer^®^ tubes (Clot Activator/SST™ Gel) and centrifuged at 7500g for 10 min, after which serum was harvested and stored at –20°C until further analysis.

#### Evaluation of Immune Response

Analysis of the produced plasma antibodies specific against OVA were performed by an indirect enzyme-linked immunosorbent assay (ELISA). Briefly, Nunc MaxiSorp™ ELISA plates (Thermo Scientific) were coated with endotoxin-free OVA (EndoFit™ Ovalbumin; Invitrogen) at 0.05 μg/well in carbonate buffer (pH 9.5) and plates were incubated overnight at 4°C. After washing the wells (PBS, 10 mM, containing 0.05% Tween 20), plates were blocked with 10% of fetal calf serum (FCS, Biowest) in PBS buffer for 1 h. Serial dilutions of mouse sera in blocking buffer (10% FCS in PBS buffer) were added to wells with appropriate controls and incubated for 1 h at room temperature. After washing, goat anti-mouse total IgG (Jackson ImmunoResearch) or subclass-specific IgG1, IgG2b, and IgG2c (SouthernBiotech) antibodies conjugated to horseradish peroxidase (HRP) were added to each well. Antibodies were diluted as indicated by the manufacturer, i.e. IgG at 1/5000; IgG1 and IgG2b at 1/4000 dilution; and IgG2c at 1/10000. After 1 h incubation at room temperature, KPL SureBlue reserve™ commercial solution (100 μl/well, SeraCare) containing 3,3’,5,5’-tetramethylbenzidine (TMB) was added as peroxidase substrate. The reaction was stopped after 10 min incubation by adding 2 N H_2_SO_4_ (100 μl/well). For absorbance measurements, optical density (OD) at 450 nm was immediately determined using a BioTek^®^ Synergy HT multi-detection microplate reader. Antibody titer was defined as the highest serum dilution that showed an absorbance of 0.1 or greater over that of the pre-sera (26).

#### Initial Toxicity Assessment in Mice

As a standard initial overall assessment of the potential toxicity of the saponins, in addition to visual inspection for potential signs of discomfort, the weight loss of each group of mice was monitored before and after each immunization. The median percentage weight change at different timepoints post-injection was determined and analyzed.

#### Statistics

Two-way ANOVA Dunnett’s multiple comparisons test was used to assess statistical significance across the different antibody response (OD) curves at the various dilutions compared to saponin lead **3** EA, or to the no-adjuvant, OVA-alone control. Antibody titers data defined as previously reported (26) are represented for each individual mouse; black horizontal bars indicate median value of five mice. When comparing two experimental groups, statistical significance of each antibody response compared to the saponin leads **2** QA or **3** EA or, alternatively, to the no-adjuvant group was assessed in each case using a two-tailed unpaired Student’s *t*-test with a 95% confidence interval (GraphPad Prism, GraphPad Software, La Jolla, CA). * *p* ≤ 0.05, ** *p* ≤ 0.01, *** *p* ≤ 0.001, **** *p* ≤ 0.0001.

## Data Availability Statement

The original contributions presented in the study are included in the article/**Supplementary Material**. Further inquiries can be directed to the corresponding author.

## Ethics Statement

All experiments involving animals, including their housing and care, were performed following the guidelines of the European Union Council (Directive 2010/63/EU) and Spanish Government regulations (RD 53/2013), and with the approval of the ethics committee of CIC bioGUNE and the Competent Authority (Diputación de Bizkaia). The Animal Facility at CIC bioGUNE is accredited by AAALAC Intl.

## Author Contributions

RF carried out the chemical synthesis optimization, scale up and full characterization of the saponin variants. LA performed the vaccination and immunological experiments in mice with assistance of NS and analyzed the relevant data. CP contributed to synthetic design and performed the preliminary, small-scale synthesis of the initial batches of the constructs. AP was involved in building block preparation and in early synthetic routes. DC provided intellectual counseling in the conception of the project idea. JA contributed to the design and execution of the immunization plan and immunological studies. AF-T led and supervised the entire project and research team and wrote the manuscript. All authors approved the submitted version.

## Funding

Funding from the European Research Council (ERC-2016-STG-716878 to AF-T) and the Spanish Ministry of Science and Innovation MCIN/AEI (PID2020-117911RB-I00, CTQ2017-87530-R, RYC-2015-17888 to AF-T; RTI2018-096494-B-100 to JA) is gratefully acknowledged.

## Conflict of Interest

Authors AF-T, RF, LA, CP, and JA are inventors on patents and/or patent applications that include saponin molecules presented in this work.

The remaining authors declare that the research was conducted in the absence of any commercial or financial relationships that could be construed as a potential conflict of interest.

## Publisher’s Note

All claims expressed in this article are solely those of the authors and do not necessarily represent those of their affiliated organizations, or those of the publisher, the editors and the reviewers. Any product that may be evaluated in this article, or claim that may be made by its manufacturer, is not guaranteed or endorsed by the publisher.
