# Supplementary material for: Novel Oxime-Derivatized Synthetic Triterpene Glycosides as Potent Saponin Vaccine Adjuvants
Source: Front Immunol. 2022 May 6;13:865507. doi: 10.3389/fimmu.2022.865507 (PMC9121768; doi:10.3389/fimmu.2022.865507)
Supplement: Supplementary file 1 [file DataSheet_1.pdf]

SUPPLEMENTARY MATERIAL

Novel Oxime-derivatized Synthetic Triterpene Glycosides as Potent Saponin Vaccine Adjuvants

Roberto Fuentes,[1,+] Leire Aguinagalde,[1,+] Carlo Pifferi,[1] Adrián Plata,[1] Nagore Sacristán,[1] Donatello Castellana,[2] Juan Anguita[3,4] and Alberto Fernández-Tejada[1,4,*]

[1]Chemical Immunology Laboratory, [2]Research and Development, [3]Inflammation and Macrophage Plasticity Laboratory, CIC bioGUNE-BRTA (Basque Research and Technology Alliance), Biscay Science and Technology Park, Building 801A, Derio 48160, Biscay, [4]Ikerbasque, Basque Foundation for Science, Euskadi Plaza, 48009 Bilbao, Spain

[+]These authors have contributed equally to this work and share first authorship.

*E-mail: afernandeztejada@cicbiogune.es

TABLE OF CONTENTS

I. Supplementary Figures S1-S3 S2

II. General Information S4

III. Chemical Synthesis of Saponin Adjuvants S6

1. Synthesis of Oxime(Quillaic Acid) Saponin **4** [(oxime)QA] S6
2. Synthesis of Keto(Echinocystic Acid) Saponin **5** [(keto)EA] S8
3. Synthesis of Oxime(Echinocystic Acid) Saponin **6** [(oxime)EA] S17

IV. Immunological Evaluation in Mice S19

V. NMR Characterization: ^{1}H, APT ^{13}C, COSY, HSQC Spectra S21

VI. Supplementary Material References S62

I. SUPPLEMENTARY FIGURES S1-S3

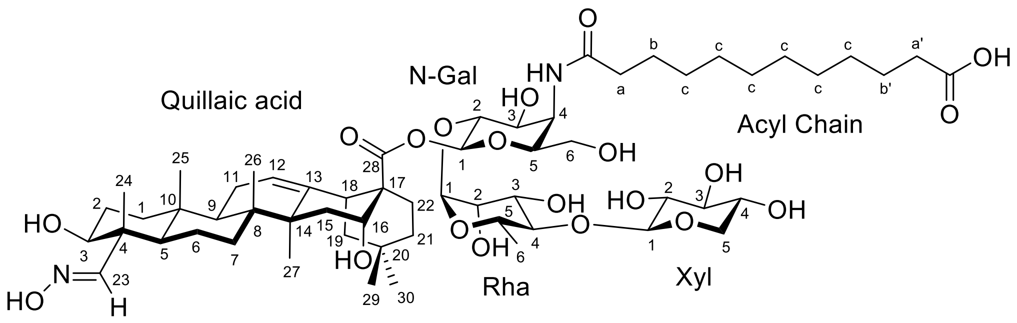

Supplementary Figure S1. Identification codes used in the NMR assignment of the different positions within the saponin structure (shown for quillaic acid-based oxime saponin **4**; analogous codes were used for the corresponding echinocystic acid-based variants **5** and **6**).

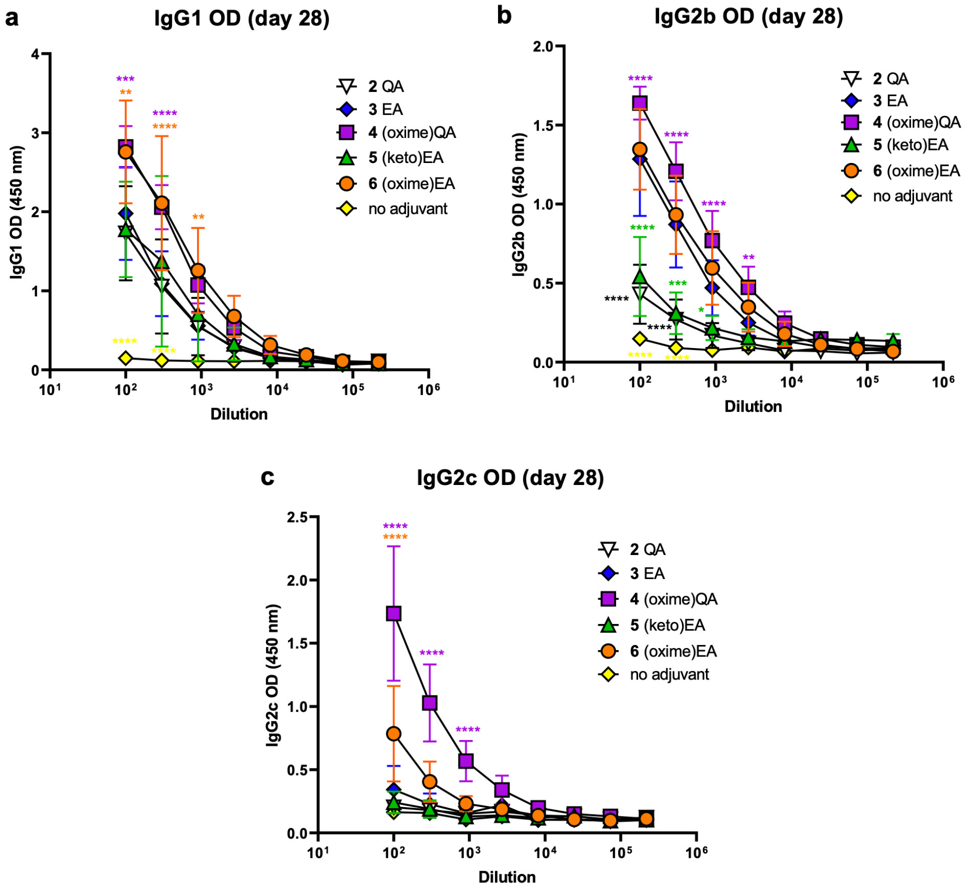

Supplementary Figure S2. ELISA standard curves showing anti-OVA IgG subype levels (represented as optical density [OD] measurements) of (a) IgG1, (b) IgG2b, and (c) IgG2c antibodies on day 28 after first immunization. Sera were measured using 3-fold serial dilutions starting at 1/100 serum dilution. Statistical significance across the different dose-response curves compared to saponin lead **3** EA group assessed using two-way ANOVA Dunnett's

multiple comparisons test at the various dilutions. * $p \leq 0.05$, ** $p \leq 0.01$, *** $p \leq 0.001$, **** $p \leq 0.0001$.

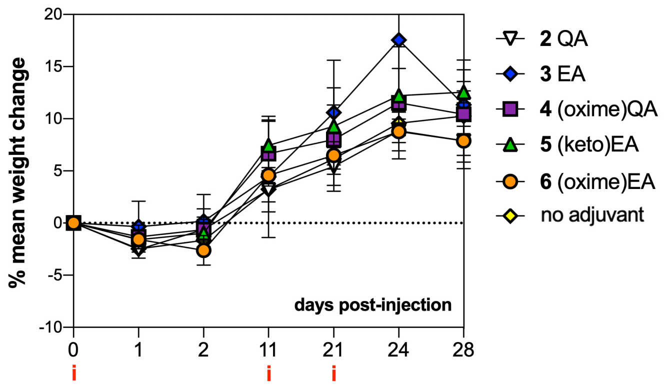

Supplementary Figure S3. Initial general toxicity assessment based on mouse weight change at various days post-injection, indicating negligible mouse weight loss of the saponin variants. Red “i” marks indicate immunization days (0, 11 and 21).

II. GENERAL INFORMATION

All commercially available materials were used without further purification except boron trifluoride diethyl etherate ($BF_3 \cdot Et_2O$) and trifluoromethanesulfonic anhydride (Tf_2O), which were distilled from calcium hydride and phosphorus pentoxide, respectively, at 1 atm under N_2. All manipulations with air-sensitive reagents and chemical reactions were carried out under a dry argon atmosphere using standard Schlenk techniques. Air- and moisture-sensitive liquids and solutions were transferred via syringe or cannula as stated. The appropriate carbohydrate reagents were dried via azeotropic removal of water with toluene. Molecular sieves were activated at 350 °C and were crushed immediately prior to use, then dried under vacuum. Organic solutions were concentrated under reduced pressure by rotary evaporation below 40 °C. Column chromatography was performed employing 230–400 mesh silica gel. Analytical thin-layer chromatography (TLC) was performed using aluminum-backed sheets pre-coated with 230–400 mesh silica gel 60 containing fluorescent indicator (F254). Preparative TLC (Analtech Uniplates) was performed using glass-backed sheets pre-coated with 500-micron silica gel containing fluorescent indicator (F254). TLC plates were visualized under UV light (254 nm) and by staining with cerium ammonium molybdate (CAM) or 5% sulfuric acid in ethanol solutions.

Nuclear magnetic resonance (NMR). ^1H, APT ^{13}C, COSY and HSQC spectra were recorded on a Bruker Avance III instrument (^1H NMR at 600 MHz and APT ^{13}C NMR at 151 MHz) and Bruker AVANCE NEO spectrometer (^1H NMR at 400 MHz and APT ^{13}C NMR at 101 MHz), equipped with a SmartProbe and operating under TopSpin 4.1.1. Chemical shifts are expressed in parts per million (δ scale) downfield from tetramethylsilane and are referenced to residual proton in the NMR solvent ($CDCl_3$: δ 7.26 for ^1H NMR, δ 77.00 for ^{13}C NMR; methanol-d_4: δ 3.31 for ^1H NMR, δ 49.15 for ^{13}C NMR). Data are presented as follows: chemical shift, multiplicity (s = singlet, br s = broad singlet, d = doublet, t = triplet, q = quartet, m = multiplet and/or multiple resonances), coupling constant (J) in Hertz (Hz), integration, assignment.

RP-HPLC purification and LC-MS. All reverse-phase RP-HPLC analyses/purifications were carried out on a Waters 1525 binary gradient system (Solv. A = 0.05% trifluoroacetic acid in water; Solv. B = 0.05% trifluoroacetic acid in acetonitrile) equipped with a Waters 2998 photodiode array detector (PDA), and combined with a low-resolution single quadrupole (SQD2, Waters Corporation) mass spectrometer. Absorbances were monitored at wavelengths of 190–600 nm.

HR-ESI-MS. High resolution electrospray ionization mass spectrometry (HR-ESI-MS) was performed on a Waters LCT Premier XE (Waters, Milford, MA, USA) in W-optics positive ionization scan mode. Mass spectrometry parameters were optimized to achieve the best signal-to-noise ratio: capillary voltage 1 kV, sample cone voltage 100 V, desolvation gas flow 600 Lh^{-1}, cone gas flow 50 Lh^{-1}, desolvation temperature 350 ^0C, source temperature 150 ^0C. The instrument was calibrated over the range *m/z* 200-2000 before measurement using a standard NaI solution (1 μM). In order to minimize the accuracy in the measurements, Leucine-Enkephalin was used as a lockmass reference [2M+Na], *m/z* 1111,5459. Data analysis was performed with Masslynx software version 4.1 (Waters, Milford, MA, USA). Characterization by MS was corroborated after comparing the experimental isotopic pattern with the theoretical one.

MALDI-TOF-HR-MS: High resolution MALDI-TOF mass spectra analyses were performed on an UltrafleXtreme III MALDI-time-of-flight (TOF) mass spectrometer equipped with a pulsed Nd:YAG laser (355 nm) and controlled by FlexControl 3.3 software (Bruker Daltonics, Bremen, Germany). The acquisitions were carried out in positive reflector ion mode with pulse duration of 50 ns. Laser intensity was set marginally above the threshold of ionization to avoid fragmentation. The *m/z* range was chosen according to the mass of the sample. The acquired data was processed using the mMass software.

III. CHEMICAL SYNTHESIS OF SAPONIN ADJUVANTS

1. Synthesis of (Oxime)Quillaic Acid Saponin 4 [(oxime)QA]

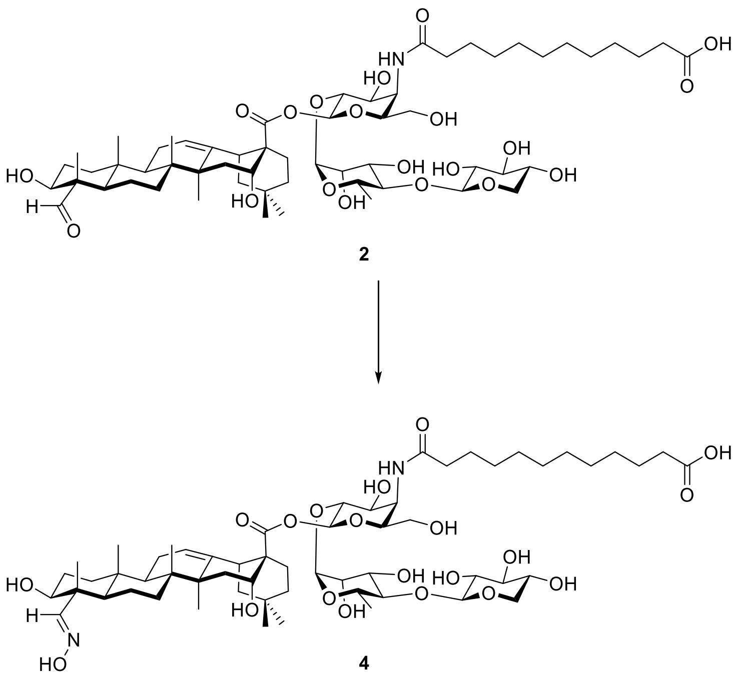

(Oxime)quillaic acid saponin 4 [(oxime)QA]

A solution of $NH_2OH \cdot HCl$ (5.76 mg, 0.08 mmol, 21.3 equiv) and NaOAc (10.26 mg, 0.13 mmol, 32.2 equiv) in acetonitrile/water (3:1, 2.4 mL) is added to quillaic acid saponin **2** (4.41 mg, 3.9 μmol, 1.0 equiv). To this mixture, an excess (100 μL) of NH_2OH 50% aq. soln. was added and the reaction suspension was left stirring overnight at 45 ºC. The crude mixture was directly purified via RP-HPLC (< 0.3 mL per injection) on a XBridge Prep BEH300 C18 column (5 μm, 19 × 150 mm) using a linear gradient of 30–64% acetonitrile/water containing 0.05% trifluoroacetic acid over 12 min (after initial 5 min at starting conditions) at a flow rate of 17 mL/min. The fraction containing the major peak (t_R = 15.81 min) was collected and lyophilized to dryness to provide the desired (oxime)QA saponin **4** (4.12 mg, 92% yield) as a white foam.

HPLC: t_R = 25.49 min (gradient = 30-100% acetonitrile/water over 30 min), λ_{max} = 193.52 nm. **^{1}H NMR** (400 MHz, methanol-d_4): δ 7.13 (s, 1H, C*H*NOH), 5.37 (d, *J* = 1.8 Hz, 1H, H-1 Rha), 5.34 (d, *J* = 7.2 Hz, 1H, H-1 N-Gal), 5.30 (t, *J* = 3.6 Hz, 1H, H-12 QA), 4.51 – 4.47 (m, 2H, H-1 Xyl, H-16 QA), 4.34 – 4.31 (m, 1H, H-4 N-Gal), 3.97 – 3.90 (m, 3H, H-3 & H-2 N-Gal, H-2 Rha), 3.89 – 3.76 (m, 3H, H-5a Xyl, H-5 & H-3 Rha), 3.69 (td, *J* = 6.6, 1.7 Hz, 1H, H-5 N-Gal), 3.59 – 3.46 (m, 4H, H-4 Rha, H-3 QA, H-4 Xyl, H-6a N-Gal), 3.45 – 3.38 (m, 1H, H-6b N-Gal), 3.36 – 3.11 (m, 3H, H-3 & H-2 Xyl, H-5b Xyl), 2.94 (dd, *J* = 14.3, 4.5 Hz, 1H, H-18 QA), 2.41 – 2.22 (m, 5H, H-19a QA, CH_2(a)CONH & CH_2(a')CO_2H acyl), 2.01 – 1.87 (m, 4H, H-11a,b, H-22a & H-21a QA), 1.87 – 1.74 (m, 1H, H-22b QA), 1.74 – 1.56 (m, 9H, H-2a,b, H-9, H-1a & H-15a QA, C$H_{2(b)}$$CH_2$CONH & C$H_{2(b')}$$CH_2CO_2H$ acyl), 1.55 – 1.40 (m, 3H, H-7a, H-15b & H-6a QA), 1.39 – 1.24 (m, 20H, [1.39 s, 3H, CH_3 C-27 QA], H-7b & H-6b QA, CH_3 Rha, 6 × C$H_{2(c)}$ internal acyl), 1.22 – 1.13 (m, 1H, H-21 QA), 1.12 – 0.98 (m, 9H, [1.03 s,

3H, CH_3 C-24 QA], [1.01 s, 3H, CH_3 C-25 QA], H-1b, H-19b & H-5 QA), 0.96 (s, 3H, CH_3 C-30 QA), 0.88 (s, 3H, CH_3 C-29 QA), 0.77 (s, 3H, CH_3 C-26 QA). **^{13}C NMR** (101 MHz, methanol-d_4): δ 178.5 (CONH acyl), 177.8 (CO_2H acyl), 177.1 (CO [C-28] QA), 161.0 (CNOH), 144.8 (C-13 QA), 123.3 (C-12 QA), 107.0 (C-1 Xyl), 101.4 (C-1 Rha), 95.5 (C-1 N-Gal), 84.2 (C-4 Rha), 78.2 (C-3 Xyl), 76.5 (C-3 QA), 76.3 (C-5 N-Gal), 76.1 (C-2 Xyl), 74.8 (C-3 & C-2 N-Gal), 74.7 (C-16 QA), 72.2 (C-3 Rha), 71.9 (C-2 Rha), 71.0 (C-4 Xyl), 68.9 (C-5 Rha), 67.3 (C-5 Xyl), 61.7 (C-6 N-Gal), 52.9 (C-5 QA), 52.5 (C-4 N-Gal), 50.0 (C-17 QA), 48.2 (C-9 QA), 48.0 (C-19 QA), 47.4 (C-4 QA), 42.7 (C-14 QA), 42.3 (C-18 QA), 41.1 (C-8 QA), 39.7 (C-1 QA), 37.7 (C-10 QA), 36.8 ($CH_{2(a)}$CONH acyl), 36.5 (C-15 & C-21 QA), 35.1 ($CH_{2(a')}CO_2H$ acyl QA), 33.8 (C-7 QA), 33.4 (CH_3 C-29), 32.0 (C-22 QA), 31.3 (C-20 QA), 30.6, 30.5, 30.4, 30.34, 30.26 (6 × $CH_{2(c)}$ internal acyl), 27.23 ($CH_{2(b)}CH_2$CONH acyl), 27.21 (CH_3 C-27 QA), 27.0 (C-2 QA), 26.2 ($CH_{2(b')}CO_2H$ acyl), 24.9 (CH_3 C-30 QA), 24.5 (C-11 QA), 20.9 (C-6 QA), 18.4 (CH_3 Rha), 17.8 (CH_3 C-26 QA), 16.6 (CH_3 C-25 QA), 12.0 (CH_3 C-24 QA). **HRMS (ESI$^-$)** *m/z*: Calcd for $[C_{59}H_{95}N_2O_{20}]^-$ $[M-H]^-$ 1151.6476, found 1151.6483.

2. Synthesis of (Keto)Echinocystic Acid Saponin 5 [(keto)EA]

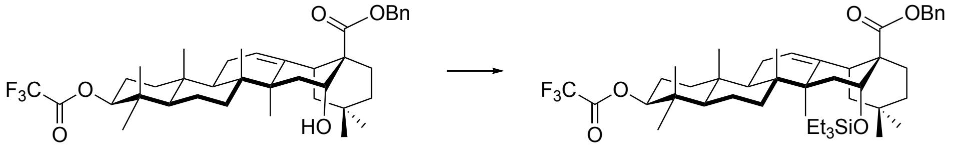

S1 **7**

C3-*O*-Trifluoroacetyl-C16-*O*-Triethylsilyl echinocystic acid benzyl ester (7)

A solution of trifluoroacetylated intermediate **S1**[1] (205 mg, 0.31 mmol) in dry DCM was cooled to 0ºC, then lutidine (290 µL, 2.48 mmol, 8.0 equiv) and TESOTf (422.2 uL, 1.86 mmol, 6 equiv.) were added dropwise and the mixture was stirred for 1 h while warming up to rt. $NaHCO_3$ saturated solution was added to quench the reaction and the crude was diluted with DCM and washed with $NaHCO_3$ (sat. solution). The organic layer was dried with $MgSO_4$, filtered, concentrated and azeotropically dried with toluene. Dry vacuum column chromatography (DCVC) from 100% hexane to 95:5 hexane/ethyl acetate afforded the purified protected intermediate **7** (225.41 mg, 93% yield) as a white foam.

TLC: R_f 0.41 (95:5 hexane/ EtOAc); **^{1}H NMR** (600 MHz, $CDCl_3$): δ 7.38 – 7.28 (m, 5H Ar-H), 5.32 (t, J = 3.7 Hz, 1H, H-12), 5.03 (s, 2H, CH_2Ph [OBn]), 4.68 (dd, J = 11.7, 4.8 Hz, 1H, H-3), 4.62 (s, 1H, H-16), 3.05 (dd, J = 14.5, 4.5 Hz, 1H, H-18), 2.23 (dd, J = 14.4, 12.7 Hz, 1H, H-19a), 1.89 – 1.66 (m, 8H, H-22a,b, H-11a,b, H-21a, H-2a,b, H-1a), 1.65 – 1.57 (m, 2H, H-15a, H-9), 1.55 – 1.43 (m, 2H, H-6a, H-7a), 1.39 – 1.30 (m, 4H, H-6b, [1.34 s, 3H, CH_3 C-27]), 1.29 – 1.20 (m, 2H, H-7b, H-15b), 1.15 – 1.03 (m, 3H, H-21b, H-1b, H-19b), 1.00 (t, J = 7.9 Hz, 9H, 3 × CH_3 [C16-OSiEt_3]), 0.95 (s, 3H, CH_3 C-30), 0.93 (s, 3H, CH_3 C-25), 0.90 (s, 6H, CH_3 C-24 & C-23), 0.88 (s, 3H, CH_3 C-29), 0.83 (d, J = 11.8 Hz, 1H, H-5), 0.73 – 0.62 (m, 6H, 3 × C$H_2$$CH_3$ [C16-OSiEt_3]), 0.54 (s, 3H, CH_3 C-26). **^{13}C NMR** (151 MHz, $CDCl_3$): δ 176.7 (CO_2Bn), 157.9, 157.7, 157.4, 157.1 (*C*O_2CF_3), 143.6 (C-13), 136.3 (ipso Bn), 128.6, 128.11, 128.07 (Ar), 122.2 (C-12), 115.8, 113.9 (*C*F_3CO), 86.4 (C-3), 75.3 (C-16), 66.3 (CH_2Ph [OBn]), 55.4 (C-5), 49.0 (C-17), 46.7 (C-9), 46.5 (C-19), 41.5 (C-14), 40.7 (C-18), 39.4 (C-8), 38.13 (C-1), 38.09 (C-4), 37.0 (C-10), 35.4 (C-21), 34.7 (C-15), 32.95 (C-7), 32.86 (CH_3 C-29), 31.6 (C-22), 30.7 (C-20), 28.0 (CH_3 C-23), 26.5 (CH_3 C-27), 24.5 (CH_3 C-30), 23.4 (C-11), 23.3 (C-2), 18.3 (C-6), 16.9 (CH_3 C-26), 16.6 (CH_3 C-24), 15.6 (CH_3 C-25), 7.3 (3 × CH_3 [C16-OSiEt_3]), 5.2 (3 × *C*$H_2$$CH_3$ [C16-OSiEt_3]). **HRMS (ESI$^+$):** *m/z* (monoisotopic) Calcd for $[C_{45}H_{67}F_3NaO_5Si]^+$ $[M+Na]^+$ 796.4608, found 796.4550.

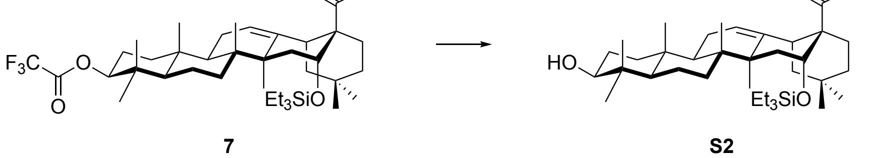

7 **S2**

C16-*O*-Triethylsilyl echinocystic acid benzyl ester (S2)

A suspension of protected echinocystic triterpene **7** (204.9 mg, 0.265 mmol) in THF/MeOH/H_2O (3:4:1) (16 mL) was treated with KOH (22.3 mg, 0.397 mmol, 1.5 equiv.), turning into a clear solution. After stirring for 0.5 h, the reaction mixture was concentrated,

diluted with ethyl acetate, and washed with water twice (aq. phase reextracted with EtOAc). The organic layers were combined and dried over $MgSO_4$, filtered and concentrated. Dry vacuum column chromatography (DCVC) in 9:1 hexane/ethyl acetate afforded the purified C3-deprotected triterpene **S2** (178.0 mg, 99% yield) as a white foam.

TLC: R_f 0.22 (9:1 hexane/ EtOAc); **^{1}H NMR** (600 MHz, $CDCl_3$): δ 7.37 – 7.28 (m, 5H, Ar-H), 5.32 (t, J = 3.7 Hz, 1H, H-12), 5.03 (s, 2H, C*H*$_2$Ph [OBn]), 4.63 – 4.60 (m, 1H, H-16), 3.20 (dd, J = 11.3, 4.4 Hz, 1H, H-3), 3.04 (dd, J = 14.4, 4.5 Hz, 1H, H-18), 2.23 (dd, J = 14.4, 12.7 Hz, 1H, H-19a), 1.90 – 1.78 (m, 4H, H-11a,b, H-22a, H-21a), 1.78 – 1.67 (m, 1H, H-22b), 1.66 – 1.41 (m, 7H, H-15a, H-1a, H-2a,b, H-9, H-6a, H-7a), 1.34 (s, 3H, CH_3 C-27), 1.32 – 1.18 (m, 3H, H-6b, H-15b, H-7b), 1.15 – 1.09 (m, 1H, H-21b), 1.08 – 1.02 (m, 1H, H-19b), 1.00 (t, J = 8.0 Hz, 9H, 3 × CH_3 [C16-OSiEt$_3$]), 0.98 (s, 3H, CH_3 C-23), 0.98 – 0.95 (m, 1H, H-1b), 0.94 (s, 3H, CH_3 C-30), 0.88 (s, 6H, CH_3 C-25 & C-29), 0.77 (s, 3H, CH_3 C-24), 0.73 – 0.61 (m, 7H, H-5, 3 × C*H*$_2$CH$_3$ [C16-OSiEt$_3$]), 0.54 (s, 3H, CH_3 C-26). **^{13}C NMR** (151 MHz, $CDCl_3$): δ 176.8 (CO_2Bn), 143.5 (C-13), 136.4 (ipso Bn), 128.6, 128.09, 128.05 (Ar), 122.5 (C-12), 79.2 (C-3), 75.4 (C-16), 66.3 (CH_2Ph [OBn]), 55.4 (C-5), 49.1 (C-17), 46.8 (C-9), 46.5 (C-19), 41.5 (C-14), 40.7 (C-18), 39.4 (C-8), 38.9 (C-4), 38.7 (C-1), 37.1 (C-10), 35.4 (C-21), 34.7 (C-15), 33.1 (C-7), 32.9 (CH_3 C-29), 31.6 (C-22), 30.7 (C-20), 28.3 (CH_3 C-23), 27.4 (C-2), 26.6 (CH_3 C-27), 24.5 (CH_3 C-30), 23.4 (C-11), 18.4 (C-6), 17.0 (CH_3 C-26), 15.7 (CH_3 C-24), 15.5 (CH_3 C-25), 7.3 (3 × CH_3 [C16-OSiEt$_3$]), 5.2 (3 × *C*H$_2$CH$_3$ [C16-OSiEt$_3$]). **HRMS (MALDI)** *m/z*: Calcd for $[C_{43}H_{68}O_4SiNa]^+$ $[M+Na]^+$ 699.4777, found 699.4762.

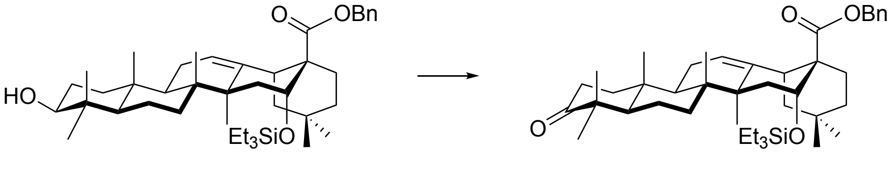

S2 **S3**

C3-Oxo-C16-*O*-triethylsilyl echinocystic acid benzyl ester (S3)

To a solution of azeotropically dried C3-alcohol **S2** (192 mg, 0.283 mmol) in dry DCM (16 mL), PCC (183 mg, 0.85 mmol, 3 equiv.) was added, and the orange mixture was stirred for 2 h at rt. After reaction completion, the mixture turned dark brown and was concentrated under vacuum. Purification by dry vacuum column chromatography (DCVC) from hexane to 95:5 hexane/EtOAc provided the C3-ketone triterpene **S3** (133 mg, 73% yield) as a white foam.

TLC: R_f 0.32 (95:5 hexane/ EtOAc); **^{1}H NMR** (600 MHz, $CDCl_3$): δ 7.37 – 7.28 (m, 5H, Ar-H), 5.34 (t, J = 3.7 Hz, 1H, H-12), 5.04 (s, 2H, C*H*$_2$Ph [OBn]), 4.62 (s, 1H, H-16), 3.05 (dd, J = 14.5, 4.5 Hz, 1H, H-18), 2.53 (ddd, J = 15.9, 10.9, 7.4 Hz, 1H, H-2a), 2.37 (ddd, J = 15.9, 6.9, 3.7 Hz, 1H, H-2b), 2.23 (dd, J = 14.4, 12.7 Hz, 1H, H-19a), 1.92 – 1.78 (m, 5H, H-11a,b, H-22a, H-21a, H-1a), 1.77 – 1.68 (m, 1H, H-22b), 1.67 – 1.59 (m, 2H, H-15a, H-9), 1.53 – 1.37 (m, 4H, H-6a,b, H-7a, H-1b), 1.35 (s, 3H, CH_3 C-27), 1.32 – 1.24 (m, 3H, H-7b, H-15b, H-5), 1.15 – 1.10 (m, 1H, H-21b), 1.08 (s, 3H, CH_3 C-23), 1.07 – 1.04 (m, 1H, H-19b), 1.03 (s, 3H, CH_3 C-24), 1.01 (s, 3H, CH_3 C-25), 1.00 (t, J = 7.9 Hz, 9H, 3 × CH_3 [C16-OSiEt$_3$]), 0.95 (s, 3H, CH_3 C-30), 0.88 (s, 3H, CH_3 C-29), 0.72 – 0.63 (m, 6H, 3 × C*H*$_2$CH$_3$ [C16-OSiEt$_3$]), 0.58 (s, 3H, CH_3 C-26). **^{13}C NMR** (151 MHz, $CDCl_3$): δ 218.0 (C-3), 176.7 (CO_2Bn), 143.6 (C-13), 136.3 (ipso Bn), 128.6, 128.12, 128.09 (Ar), 122.3 (C-12), 75.3 (C-16), 66.3 (CH_2Ph [OBn]), 55.4 (C-5), 49.1 (C-17), 47.5 (C-4), 46.5 (C-19), 46.0 (C-9), 41.6 (C-14), 40.8 (C-18), 39.4 (C-

8), 39.3 (C-1), 36.8 (C-10), 35.4 (C-21), 34.7 (C-15), 34.3 (C-7), 32.9 (CH_3 C-29), 32.6 (C-7), 31.6 (C-22), 30.7 (C-20), 26.7 (CH_3 C-23), 26.4 (CH_3 C-27), 24.4 (CH_3 C-30), 23.5 (C-11), 21.6 (CH_3 C-24), 19.7 (C-6), 16.9 (CH_3 C-26), 15.3 (CH_3 C-25), 7.3 (3 × CH_3 [C16-OSiEt3]), 5.2 (3 × *C*H_2CH_3 [C16-OSiEt3]). **HRMS (ESI$^+$)** *m/z*: Calcd for $[C_{43}H_{66}O_4SiNa]^+$ $[M+Na]^+$ 697.4628, found 697.4622.

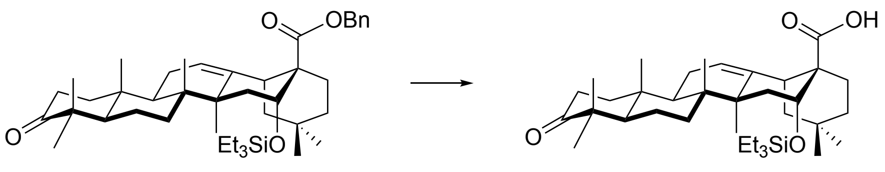

S3 **8**

C3-Oxo-C16-*O*-triethylsilyl echinocystic acid (8)

Benzyl ester triterpene **S3** (155.0 mg, 0.230 mmol) was dissolved in a mixture of THF/MeOH (1:1) (28 mL), 10% Pd/C (50% wet, 100 mg, 0.093 mmol, 0.4 equiv.) was added and a H_2 balloon was connected. After 3 purge cycles of vacuum and H_2, the reaction was stirred for 45 min. The reaction mixture was filtered through a plug of celite®, which was rinsed with MeOH (2 × 5mL) and EtOAc (1 × 5 mL) and concentrated. Flash column chromatography (from 9:1 to 8:2 hexane:EtOAc) provided the C3-ketone triterpene acid **8** (130 mg, 97% yield) as a white foam.

TLC: R_f 0.32 (95:5 hexane/ EtOAc); **^{1}H NMR** (600 MHz, $CDCl_3$) δ 5.35 (t, *J* = 3.7 Hz, 1H, H-12), 4.60 – 4.54 (m, 1H, H-16), 2.96 (dd, *J* = 14.5, 4.5 Hz, 1H, H-18), 2.54 (ddd, *J* = 15.9, 10.9, 7.3 Hz, 1H, H-2a), 2.38 (ddd, *J* = 15.9, 6.9, 3.7 Hz, 1H, H-2b), 2.21 (m, 1H, H-19a), 2.00 – 1.66 (m, 8H, H-11a,b, H-1a, H-22a,b, H-21a, H-15a, H-9), 1.55 – 1.39 (m, 4H, H-7a, H-6a,b, H-1b), 1.36 (s, 3H, CH_3 C-27), 1.35 – 1.29 (m, 3H, H-7b, H-15b, H-5), 1.17 – 1.12 (m, 1H, H-21b), 1.08 (s, 3H, CH_3 C-23), 1.07 – 1.05 (m, 1H, H-19b), 1.04 (s, 3H, CH_3 C-25), 1.02 (s, 3H, CH_3 C-24), 1.00 (t, *J* = 8.0 Hz, 9H, 3 × CH_3 [C16-OSiEt3]), 0.95 (s, 3H, CH_3 C-30), 0.88 (s, 3H, CH_3 C-29), 0.75 (s, 3H, CH_3 C-26), 0.72 – 0.62 (m, 6H, 3 × *CH*$_2$$CH_3$ [C16-OSiEt3]). **^{13}C NMR** (151 MHz, $CDCl_3$) δ 217.9 (C-3), 183.5 (CO_2H), 143.4 (C-13), 122.4 (C-12), 75.0 (C-16), 55.4 (C-5), 48.9 (C-17), 47.5 (C-4), 46.4 (C-19), 46.0 (C-9), 41.5 (C-14), 40.3 (C-18), 39.4 (C-8), 39.3 (C-1), 36.9 (C-10), 35.3 (C-21), 34.8 (C-15), 34.3 (C-2), 32.8 (CH_3 C-29), 32.5 (C-7), 31.7 (C-22), 30.6 (C-20), 26.7 (CH_3 C-23), 26.5 (CH_3 C-27), 24.4 (CH_3 C-30), 23.5 (C-11), 21.5 (CH_3 C-24), 19.7 (C-6), 17.1 (CH_3 C-26), 15.2 (CH_3 C-25), 7.3 (3 × CH_3 [C16-OSiEt3]), 5.1 (3 × *C*H_2CH_3 [C16-OSiEt3]). **HRMS (ESI$^+$)** *m/z*: Calcd for $[C_{36}H_{60}O_4SiNa]^+$ $[M+Na]^+$ 607.4158, found 607.4139.

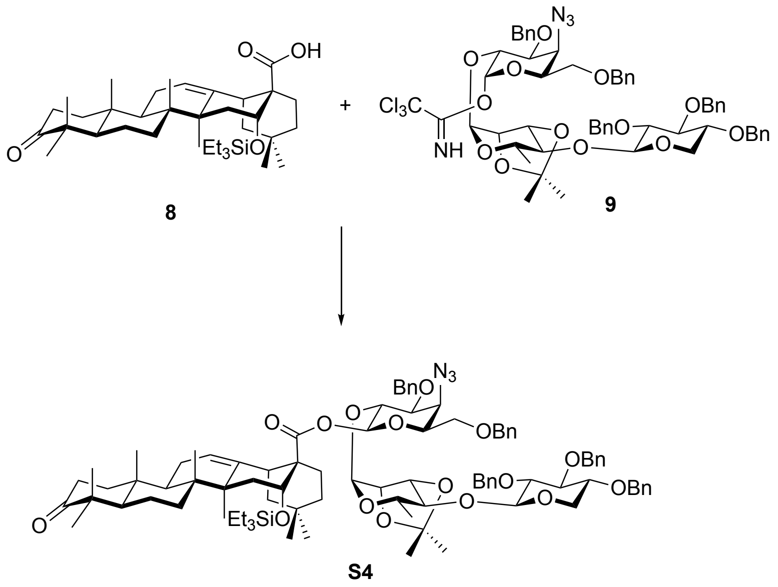

Protected (keto)echinocystic acid saponin azide (S4)

A solution of azeotropically dried imidate **9**[2] (93 mg, 0.083 mmol), (keto)triterpene acid **8** (63.2 mg, 0.108 mmol, 1.3 equiv.) and powdered 4 Å molecular sieves (90 mg) in DCM (4.5 mL) was cooled to –78 ºC. To this suspension, boron trifluoride etherate ($BF_3 \cdot OEt_2$) (10.5 µL, 83.1 µmol, 1 equiv.) was added dropwise, and after stirring for 10 min the reaction mixture was warmed up to –48 ºC and stirred for additional 15 min. After quenching with triethylamine (0.5 mL), the crude was filtered through a celite pad, concentrated, and purified by column chromatography (9:1 hexane/EtOAc to 85:15 hexane/EtOAc), affording the C3-keto saponin azide **S4** (77.16 mg, 60% yield).

TLC: R_f 0.40 (8:2 hexane/ EtOAc+1% Et_3N); **^{1}H NMR** (600 MHz, $CDCl_3$) δ 7.39 – 7.22 (m, 25H, Ar-H), 5.38 – 5.34 (m, 2H, H-1 N-Gal, H-12 EA), 5.22 (d, *J* = 2.2 Hz, 1H, H-1 Rha), 4.93 – 4.80 (m, 4H, CH*Ha*Ph [C2-OBn Xyl], C*H*2Ph [C3-OBn Xyl], H-1 Xyl), 4.75 – 4.57 (m, 5H, C*H*2Ph [C4-OBn Xyl], C*H*2Ph [C3-OBn N-Gal], CH*Hb*Ph [C2-OBn Xyl],), 4.54 – 4.49 (m, 3H, C*H*2Ph [C6-OBn N-Gal], H-16 EA), 4.19 – 4.15 (m, 1H, H-2 Rha), 4.15 – 4.11 (m, 1H, H-3 Rha), 4.05 – 4.03 (m, 1H, H-4 N-Gal), 3.96 – 3.90 (m, 2H, H-5a Xyl, H-2 N-Gal), 3.72 – 3.64 (m, 3H, H-5 Rha, H-3 & H-5 N-Gal), 3.64 – 3.59 (m, 2H, H-4 & H-3 Xyl), 3.58 – 3.51 (m, 3H, H-6a,b N-Gal, H-4 Rha), 3.34 – 3.28 (m, 1H, H-2 Xyl), 3.24 – 3.18 (m, 1H, H-5b Xyl), 2.91 (dd, *J* = 14.4, 4.4 Hz, 1H, H-18 EA), 2.53 (ddd, *J* = 15.9, 10.7, 7.4 Hz, 1H, H-2a EA), 2.39 (ddd, *J* = 15.9, 7.0, 3.8 Hz, 1H, H-2b EA), 2.26 – 2.18 (m, 1H, H-19a EA), 1.99 – 1.76 (m, 6H, H-11a,b, H-22a,b, H-21a, H-1a EA), 1.72 – 1.63 (m, 2H, H-15a, H-9 EA), 1.57 – 1.48 (m, 1H, H-7a EA), 1.45 (s, 3H, $CH_{3(a)}$ isopr Rha), 1.44 – 1.40 (m, 3H, H-6a,b, H-1b EA), 1.36 (s, 3H, CH_3 C-27 EA), 1.34 – 1.28 (m, 3H, H-7b, H-15b, H-5 EA), 1.28 (s, 3H, $CH_{3(b)}$ isopr Rha), 1.21 (d, *J* = 5.3 Hz, 3H, CH_3 Rha), 1.15 – 1.10 (m, 1H, H-21b EA), 1.08 (s, 3H, CH_3 C-23 EA), 1.06 – 1.04 (m, 1H, H-19b EA), 1.03 (s, 3H, CH_3 C-24 EA), 1.01 (s, 3H, CH_3 C-25 EA), 0.98 (t, *J* = 8.0 Hz, 9H, 3 × CH_3 [C16-OSiEt3 EA]), 0.93 (s, 3H, CH_3 C-30 EA), 0.87 (s, 3H, CH_3 C-29 EA), 0.78 (s, 3H, CH_3 C-26 EA), 0.73 – 0.60 (m, 6H, 3 × C*H*2CH3 [C16-OSiEt3 EA]). **^{13}C NMR** (151 MHz, $CDCl_3$) δ 218.0 (C-3 EA), 175.4 (CO [C-28] EA) , 143.3 (C-13 EA), 138.9, 138.8, 138.4, 137.6, 137.1 (ipso Bn), 128.7, 128.6, 128.6, 128.4, 128.4, 128.3, 128.1, 128.05, 128.02, 127.98, 127.93, 127.90, 127.7 (Ar), 122.0 (C-12 EA), 109.6 (C isopr Rha), 102.5 (C-1 Xyl), 98.2 (C-1 Rha), 94.0 (C-1 N-Gal), 83.9 (C-3 Xyl), 82.1 (C-2 Xyl), 80.8 (C-3 N-Gal), 78.7 (C-4 Rha), 78.4 (C-3 Rha), 78.1 (C-4 Xyl), 76.0 (C-2 Rha), 75.7 (CH_2Ph

[C3-OBn Xyl]), 75.2 (C-16 EA), 74.85 (C-2 N-Gal), 74.78 (CH_2Ph [C2-OBn Xyl]), 73.7 (CH_2Ph [C6-OBn N-Gal]), 73.3 (CH_2Ph [C4-OBn Xyl]), 72.7 (CH_2Ph [C3-OBn N-Gal]), 72.1 (C-5 N-Gal), 67.8 (C-6 N-Gal), 67.2 (C-5 Rha), 63.9 (C-5 Xyl), 59.1 (C-4 N-Gal), 55.4 (C-5 EA), 49.2 (C-17 EA), 47.5 (C-4 EA), 46.8 (C-19 EA), 46.0 (C-9 EA), 41.7 (C-14 EA), 40.9 (C-18 EA), 39.6 (C-8 EA), 39.4 (C-1 EA), 36.8 (C-10 EA), 35.3 (C-21 EA), 34.9 (C-15 EA), 34.3 (C-2 EA), 32.8 (CH_3 C-29 EA), 32.7 (C-7 EA), 30.9 (C-22 EA), 30.6 (C-20 EA), 27.7 ($CH_{3(a)}$ isopr Rha), 26.7 (CH_3 C-23 EA), 26.3 (CH_3 C-27 EA), 26.0 ($CH_{3(b)}$ isopr Rha), 24.4 (CH_3 C-30 EA), 23.6 (C-11 EA), 21.6 (CH_3 C-24 EA), 19.7 (C-6 EA), 17.9 (CH_3 Rha), 17.1 (CH_3 C-26 EA), 15.4 (CH_3 C-25 EA), 7.3 (3 × CH_3 [C16-OSiEt$_3$ EA]), 5.1 (3 × *C*H_2CH_3 [C16-OSiEt$_3$ EA]). **HRMS (MALDI)** m/z: Calcd for $[C_{91}H_{121}N_3O_{16}SiNa]^+$ $[M+Na]^+$ 1562.8405, found 1562.8497.

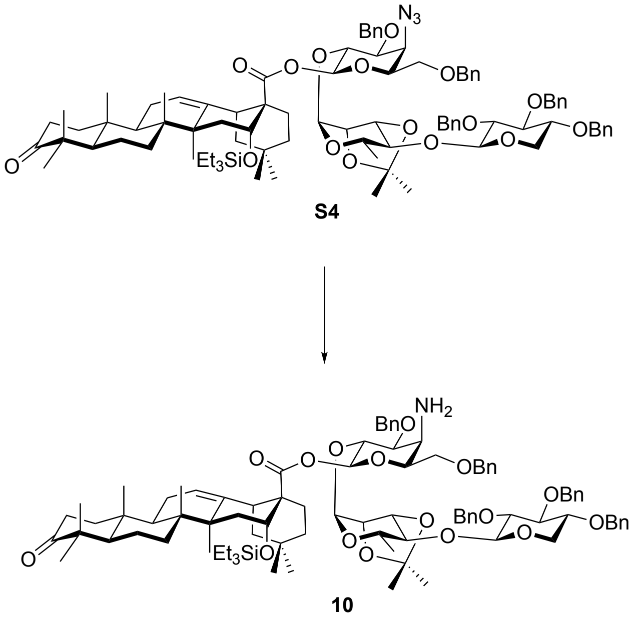

Protected (keto)echinocystic acid saponin amine (10)

A stirred solution of diphenyl diselenide $(PhSe)_2$ (156 mg, 0.5 mmol, 10 equiv) in tetrahydrofuran (5 mL) was treated with H_3PO_4 50% aq. solution (0.59 mL, 5.5 mmol, 110 equiv) and the mixture was stirred at 45 °C for 1 h. Toluene (5 mL) and H_2O (5 mL) were added and after vigorous stirring, the lower aqueous layer was removed via syringe and the organic phase was dried *in situ* with $MgSO_4$, providing a freshly prepared solution of benzeneselenol.

Keto saponin azide **S4** (77.16 mg, 0.050 mmol, 1.0 equiv) was dissolved in anhydrous triethylamine (27.3 mL) and a solution of freshly prepared benzeneselenol (20 equiv, see above) in tetrahydrofuran/toluene (1:1, 10 mL) was added via cannula. After being stirred at 45 ºC for 2 h, the resulting yellow mixture was concentrated and the residue was purified by silica gel column chromatography (toluene/ethyl acetate 10:0 to 8:2 containing 1% of triethylamine, v/v), providing the C3-keto saponin amine **10** (70.22 mg, 92% yield) as a glassy film.

TLC: R_f 0.30 (8:2 Toluene/ EtOAc); **^{1}H NMR** (600 MHz, $CDCl_3$): δ 7.36 – 7.21 (m, 25H, Ar-H), 5.40 (d, J = 7.9 Hz, 1H, H-1 N-Gal), 5.35 (t, J = 3.7 Hz, 1H, H-12 EA), 5.25 (d, J = 1.6

Hz, 1H, H-1 Rha), 4.93 – 4.81 (m, 4H, CH*Ha*Ph [C2-OBn Xyl], CH_2Ph [C3-OBn Xyl], H-1 Xyl), 4.74 – 4.60 (m, 4H, CH_2Ph [C4-OBn Xyl], CH*Ha*Ph [C3-OBn N-Gal], CH*Hb*Ph [C2-OBn Xyl]), 4.57 – 4.49 (m, 4H, CH_2Ph [C6-OBn N-Gal], CH*Hb*Ph [C3-OBn N-Gal], H-16 EA), 4.20 – 4.13 (m, 2H, H-2 & H-3 Rha), 3.96 – 3.91 (m, 1H, H-5a Xyl), 3.84 (dd, *J* = 9.2, 7.9 Hz, 1H, H-2 N-Gal), 3.73 – 3.64 (m, 3H, H-5 Rha, H-5 & H-6a N-Gal), 3.63 – 3.50 (m, 5H, H-4 & H-3 Xyl, H-4 Rha, H-6b & H-3 N-Gal), 3.35 (dd, *J* = 4.1, 1.5 Hz, 1H, H-4 N-Gal), 3.33 – 3.29 (m, 1H, H-2 Xyl), 3.24 – 3.18 (m, 1H, H-5b Xyl), 2.92 (dd, *J* = 14.3, 4.5 Hz, 1H, H-18 EA), 2.53 (ddd, *J* = 15.8, 10.7, 7.4 Hz, 1H, H-2a EA), 2.38 (ddd, *J* = 15.9, 7.0, 3.8 Hz, 1H, H-2b EA), 2.22 (dd, *J* = 14.4, 12.8 Hz, 1H, H-19a EA), 1.96 – 1.77 (m, 6H, H-11a,b, H-22a,b, H-1a, H-21a EA), 1.75 – 1.62 (m, 2H, H-15a, H-9 EA), 1.56 – 1.47 (m, 1H, H-7a EA), 1.45 (s, 3H, $CH_{3(a)}$ isopr Rha), 1.44 – 1.37 (m, 3H, H-6a,b, H-1b EA), 1.36 (s, 3H, CH_3 C-27 EA), 1.35 – 1.28 (m, 3H, H-7b, H-15b, H-5 EA), 1.28 (s, 3H, $CH_{3(b)}$ isopr Rha), 1.21 (d, *J* = 6.2 Hz, 3H, CH_3 Rha), 1.15 – 1.10 (m, 1H, H-21b EA), 1.07 (s, 3H, CH_3 C-23 EA), 1.07 – 1.0 (m, 1H, H-19b EA), 1.02 (s, 3H, CH_3 C-24 EA), 0.99 (s, 3H, CH_3 C-25 EA), 0.98 (t, *J* = 8.0 Hz, 9H, 3 × CH_3 [C16-OSiEt_3 EA]), 0.93 (s, 3H, CH_3 C-30 EA), 0.87 (s, 3H, CH_3 C-29 EA), 0.80 (s, 3H, CH_3 C-26 EA), 0.71 – 0.62 (m, 6H, 3 × C$H_2$$CH_3$ [C16-OSiEt_3 EA]). **^{13}C NMR** (151 MHz, $CDCl_3$): δ 218.0 (C-3 EA), 175.4 (CO [C-28] EA), 143.6 (C-13 EA), 138.9, 138.8, 138.4, 138.1, 137.6 (ipso Bn), 128.7, 128.6, 128.4, 128.4, 128.1, 128.1, 128.1, 127.9, 127.9, 127.9, 127.7 (Ar), 121.9 (C-12 EA), 109.5 (C isopr Rha), 102.5 (C-1 Xyl), 98.1 (C-1 Rha), 94.5 (C-1 N-Gal), 84.0 (C-3 Xyl), 82.2 (C-2 Xyl), 81.4 (C-3 N-Gal), 78.7 (C-4 Rha), 78.4 (C-3 Rha), 78.1 (C-4 Xyl), 76.1 (C-2 Rha), 75.7 (CH_2Ph [C3-OBn Xyl]), 75.3 (C-16 EA), 74.9 (C-2 N-Gal), 74.8 (CH_2Ph [C2-OBn Xyl]), 73.9 (C-5 N-Gal), 73.5 (CH_2Ph [C6-OBn N-Gal]), 73.3 (CH_2Ph [C4-OBn Xyl]), 71.8 (CH_2Ph [C3-OBn N-Gal]), 68.4 (C-6 N-Gal), 66.8 (C-5 Rha), 63.9 (C-5 Xyl), 55.4 (C-5 EA), 49.3 (C-17 EA), 48.8 (C-4 N-Gal), 47.5 (C-4 EA), 46.9 (C-19 EA), 46.0 (C-9 EA), 41.7 (C-14 EA), 40.9 (C-18 EA), 39.6 (C-8 EA), 39.4 (C-1 EA), 36.9 (C-10 EA),-35.4 (C-21 EA), 34.9 (C-15 EA), 34.3 (C-2 EA), 32.8 (CH_3 C-29 EA), 32.6 (C-7 EA), 31.0 (C-22 EA), 30.6 (C-20 EA), 27.8 ($CH_{3(a)}$ isopr Rha), 26.7 (CH_3 C-23 EA), 26.3 (CH_3 C-27 EA), 26.2 ($CH_{3(b)}$ isopr Rha), 24.5 (CH_3 C-30 EA), 23.6 (C-11 EA), 21.6 (CH_3 C-24 EA), 19.7 (C-6 EA), 18.0 (CH_3 Rha), 17.2 (CH_3 C-26 EA), 15.4 (CH_3 C-25 EA), 7.3 (3 × CH_3 [C16-OSiEt_3 EA]), 5.1 (3 × *C*$H_2$$CH_3$ [C16-OSiEt_3 EA]). **HRMS (MALDI)** m/z: Calcd for $[C_{92}H_{123}NO_{16}SiNa]^+$ $[M+Na]^+$ 1536.8501, found 1536.8573.

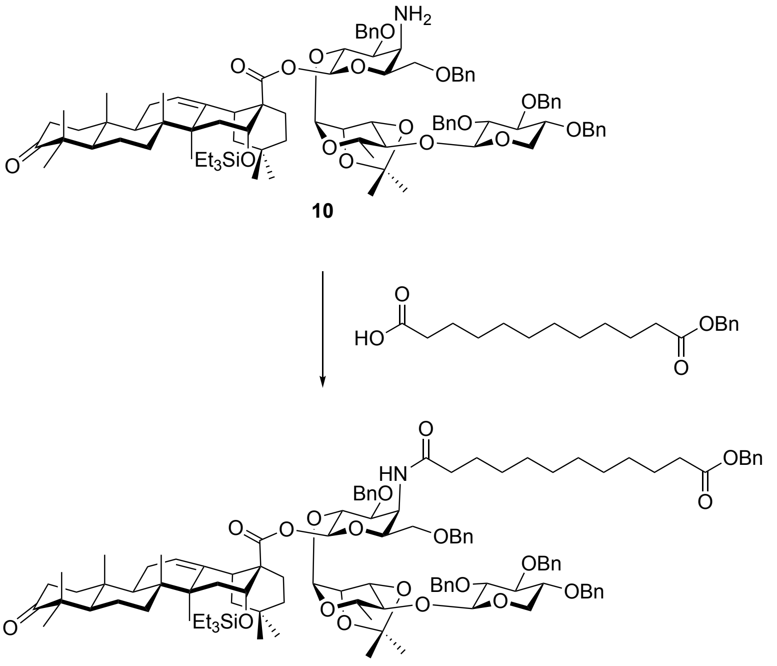

Fully protected (keto)echinocystic acid carboxyacyl saponin (S5)

Triethylamine (0.35 mL, 2.5 mmol, 90.0 equiv) was added to a solution of azeotropically dried protected dodecanedioic acid mono-benzyl ester[2] (103 mg, 0.32 mmol, 11.5 equiv) in dry tetrahydrofuran (4.0 mL), and the reaction schlenk was cooled to 0 °C. Ethyl chloroformate (26 µL, 30.3 mmol, 10.0 equiv) was added at 0 °C via syringe and the resulting white suspension was stirred at 0 °C for 0.5 h. The activated acid was then transferred via cannula to a solution of the azeotropically dried saponin amine **10** (42.4 mg, 28 µmol, 1.0 equiv) in dry tetrahydrofurane (0.3 mL) at 0 °C. The reaction mixture was stirred at this temperature for 5 min and then allowed to reach room temperature for another 0.5 h. At this point, it was quenched with anhydrous MeOH (1 mL) and then concentrated. The residue was purified by column chromatography on silica gel (hexane/ethyl acetate 3:1) to afford the fully protected C3-keto saponin **S5** (36.6 mg, 71% yield) as a glassy solid.

TLC: R_f 0.29 (2:1 Hexane/ EtOAc); **^{1}H NMR** (400 MHz, $CDCl_3$): *δ* 7.42 – 7.17 (m, 30H, Ar-H), 5.64 (d, J = 9.9 Hz, 1H, NH [C4-N-Gal]), 5.43 (d, J = 7.0 Hz, 1H, H-1 N-Gal), 5.34 (t, J = 3.4 Hz, 1H, H-12 EA), 5.24 (d, J = 1.6 Hz, 1H, H-1 Rha), 5.12 (s, 2H, CH_2Ph [OBn Acyl]), 4.95 – 4.76 (m, 6H, CH*Ha*Ph [C2-OBn Xyl], CH_2Ph [C3-OBn Xyl], H-1 Xyl, H-4 N-Gal, CH*Ha*Ph [C3-OBn N-Gal]), 4.75 – 4.57 (m, 3H, CH_2Ph [C4-OBn Xyl], CH*Hb*Ph [C2-OBn Xyl]), 4.55 – 4.41 (m, 4H, CH_2Ph [C6-OBn N-Gal], CH*Hb*Ph [C3-OBn N-Gal], H-16 EA), 4.19 – 4.12 (m, 2H, H-2 & H-3 Rha), 3.93 (dd, J = 11.6, 4.2 Hz, 1H, H-5a Xyl), 3.83 – 3.77 (m, 1H, H-5 N-Gal), 3.74 – 3.47 (m, 8H, H-2, H-3 & H-6 N-Gal, H-5 & H-4 Rha, H-3 & H-4 Xyl), 3.35 – 3.28 (m, 1H, H-2 Xyl), 3.25 – 3.17 (m, 1H, H-5a Xyl), 2.91 (dd, J = 14.2, 4.3 Hz, 1H, H-18 EA), 2.53 (ddd, J = 15.8, 10.7, 7.3 Hz, 1H, H-2a EA), 2.43 – 2.31 (m, 3H, H-2b EA, $CH_{2(a')}CO_2$Bn acyl), 2.26 – 2.12 (m, 3H, H-19a EA, $CH_{2(a)}$CONH acyl), 1.87 (m, 6H, H-11a,b, H-22a,b, H-1a & H-21a EA), 1.74 – 1.55 (m, 6H, H-9 & H-15a EA, $CH_{2(b')}CO_2$Bn acyl, $CH_{2(b)}$CONH acyl), 1.55 – 1.49 (m, 1H, H-7a EA), 1.47 (s, 3H, $CH_{3(a)}$ isopr Rha), 1.45 – 1.39 (m, 3H, H-1b & H-6a,b EA), 1.38 (s, 3H, CH_3 C-27 EA), 1.36 – 1.20 (m, 18H, H-15b, H-7b & H-5 EA, 6 × $CH_{2(c)}$ internal acyl, [1.44, s, 3H, $CH_{3(b)}$ isopr Rha), 1.18 (d, J = 6.2 Hz, 3H, CH_3

Rha), 1.16 – 1.10 (m, 1H, H-21b EA), 1.08 (s, 3H, CH_3 C-23 EA), 1.08 – 1.04 (m, 1H, H-19b EA), 1.03 (s, 3H, CH_3 C-24 EA), 1.00 (s, 3H, CH_3 C-25 EA), 0.99 (t, J = 7.9 Hz, 9H, 3 × CH_3 [C16-$OSiEt_3$ EA]), 0.94 (s, 3H, CH_3 C-30 EA), 0.87 (s, 3H, CH_3 C-29 EA), 0.80 (s, 3H, CH_3 C-26 EA), 0.74 – 0.60 (m, 6H, 3 × CH_2CH_3 [C16-$OSiEt_3$ EA]). **^{13}C NMR** (101 MHz, $CDCl_3$): δ 217.7 (C-3 EA), 175.3 (CO [C-28] EA), 173.8 (CO [CO_2Bn]), 173.2 (CONH acyl), 143.8 (C-13 EA), 138.9, 138.8, 138.4, 137.8, 137.6, 136.3 (ipso Bn), 128.7, 128.6, 128.5, 128.42, 128.40, 128.3, 128.11, 128.06, 128.0, 127.9, 127.9, 127.7, 127.7 (Ar), 121.7 (C-12 EA), 109.5 (C isopr Rha), 102.4 (C-1 Xyl), 98.0 (C-1 Rha), 94.8 (C-1 N-Gal), 84.0 (C-3 Xyl), 82.2 (C-2 Xyl), 79.1 (C-3 N-Gal), 78.5 (C-4 Rha), 78.3 (C-3 Rha), 78.1 (C-4 Xyl), 76.3 (C-2 Rha), 75.7 (CH_2Ph [C3-OBn Xyl]), 75.4 (C-16 EA), 75.3 (C-2 N-Gal), 74.9 (CH_2Ph [C2-OBn Xyl]), 73.6 (CH_2Ph [C6-OBn N-Gal]), 73.3 (CH_2Ph [C4-OBn Xyl]), 73.1 (C-5 N-Gal), 71.7 (CH_2Ph [C3-OBn N-Gal]), 68.6 (C-6 N-Gal), 66.7 (C-5 Rha), 66.2 (CH_2Ph [OBn Acyl]), 63.9 (C-5 Xyl), 55.3 (C-5 EA), 49.3 (C-17 EA), 47.5 (C-4 EA), 46.9 (C-19 EA), 46.1 (C-4 N-Gal), 45.9 (C-9 EA), 41.7 (C-14 EA), 40.7 (C-18 EA), 39.6 (C-8 EA), 39.4 (C-1 EA), 37.1 ($CH_{2(a)}$CONH acyl), 36.8 (C-10 EA), 35.3 (C-21 EA), 34.7 (C-15 EA), 34.4 ($CH_{2(a')}CO_2Bn$ acyl), 34.2 (C-2 EA), 32.8 (CH_3 C-29 EA), 32.6 (C-7 EA), 31.0 (C-22 EA), 30.6 (C-20 EA), 29.58, 29.56, 29.5, 29.44, 29.37, 29.3 (6 × $CH_{2(c)}$ internal acyl), 27.7 ($CH_{3(a)}$ isopr Rha), 26.7 (CH_3 C-23 EA), 26.3 (CH_3 C-27 EA), 26.2 ($CH_{3(b)}$ isopr Rha), 25.9 ($CH_{2(b)}$CONH acyl), 25.1 ($CH_{2(b')}CO_2Bn$ acyl), 24.4 (CH_3 C-30 EA), 23.6 (C-11 EA), 21.6 (CH_3 C-24 EA), 19.7 (C-6 EA), 17.9 (CH_3 Rha), 17.2 (CH_3 C-26 EA), 15.4 (CH_3 C-25 EA), 7.3 (3 × CH_3 [C16-$OSiEt_3$ EA]), 5.1 (3 × CH_2CH_3 [C16-$OSiEt_3$ EA]). **HRMS (MALDI)** *m/z*: Calcd for $[C_{110}H_{149}NO_{19}SiNa]^+$ $[M+Na]^+$ 1839.0382, found 1839.0261.

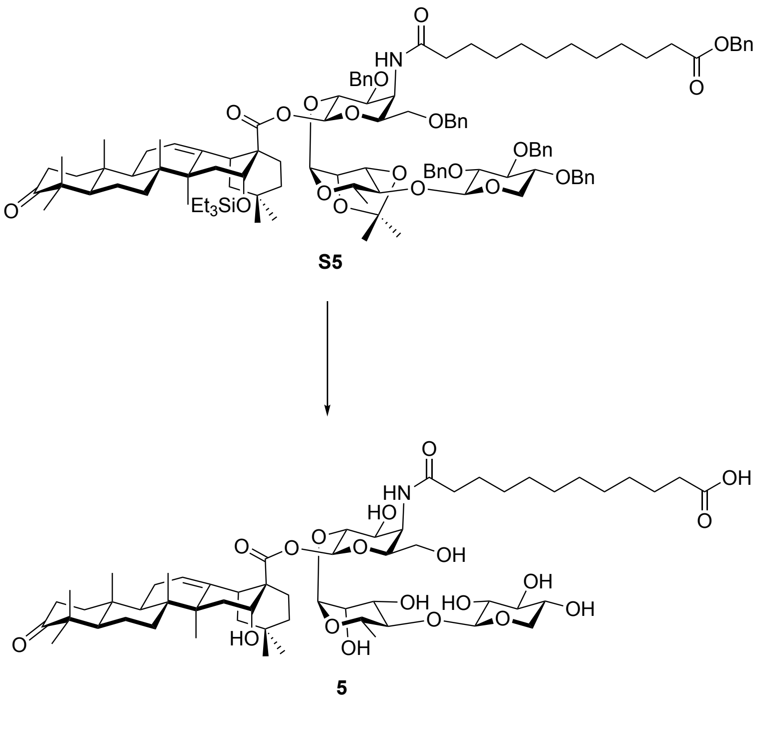

(Keto)echinocystic acid saponin (5).

To fully protected (keto)echinocystic acid carboxyacyl saponin **S5** (36.6 mg, 0.020 mmol, 1.0 equiv) dissolved in tetrahydrofuran/ethanol (1:1, 24.5 mL), 10% (dry basis) Pd/C 50% wet Degussa type E101 NE/W (214 mg, 0.10 mmol, 5.0 equiv) was added. The reaction mixture was stirred at rt under H_2 atmosphere (1 atm, balloon) for 3 h. Direct infusion (MS) confirmed the absence of starting material or intermediates. The suspension was filtered through 0.45 μm PTFE filter disk, washed extensively with methanol (3 × 10 mL) and concentrated to dryness. The crude was dissolved in a precooled (0 °C) solution of trifluoroacetic acid (TFA/H_2O 2:1, 4.4 mL), stirred at 0 °C for 0.5 h, and the solvent was then evaporated to dryness *in vacuo*. The final residue was dissolved in a mixture of acetonitrile/water (0.05% TFA) 1:1 (3 mL), filtered through 0.2 μm PTFE filter disk, and purified by RP-HPLC (< 0.3 mL per injection) on a XBridge Prep BEH300 C18 column (5 μm, 19 × 150 mm) using a linear gradient of 30–65% acetonitrile/water (0.05% TFA) over 12.5 min (after initial 5 min at starting conditions) at a flow rate of 17 mL/min. The fraction containing the major peak (t_R = 17.01 min) was collected and lyophilized to dryness to afford the final (keto)EA saponin **5** (17.3 mg, 77% yield) as a white powder.

HPLC: t_R = 21.1 min (gradient = 20–100% solv. B over 30 min), λ_{max} = 194.52 nm. **^{1}H NMR** (400 MHz, methanol-d_4): δ 5.40 (d, J = 1.8 Hz, 1H, H-1 Rha), 5.36 (d, J = 7.9 Hz, 1H, H-1 N-Gal), 5.33 (t, J = 3.3 Hz, 1H, H-12 EA), 4.52 – 4.47 (m, 2H, H-1 Xyl, H-16 EA), 4.35 – 4.31 (m, 1H, H-4 N-Gal), 3.98 – 3.92 (m, 3H, H-3 & H-2 N-Gal, H-2 Rha), 3.91 – 3.77 (m, 3H, H-5a Xyl, H-5 & H-3 Rha), 3.70 (td, J = 6.5, 1.6 Hz, 1H, H-5 N-Gal), 3.60 – 3.38 (m, 4H, H-4 Rha, H-4 Xyl, H-6a,b N-Gal), 3.36 – 3.32 (m, 1H, H-3 Xyl), 3.28 – 3.17 (m, 2H, H-2 Xyl, H-5b Xyl), 2.95 (dd, J = 14.4, 4.5 Hz, 1H, H-18 EA), 2.56 (ddd, J = 15.9, 10.3, 7.5 Hz, 1H, H-2a EA), 2.46 – 2.24 (m, 6H, H-2b & H-19a EA, CH_2(a)CONH & CH_2(a')CO_2H acyl), 2.02 – 1.88 (m, 5H, H-11a,b, H-22a, H-21a & H-1a EA), 1.86 – 1.67 (m, 3H, H-22b, H-9 & H-15a EA), 1.67 – 1.44 (m, 10H, $CH_{2(b)}CH_2$CONH & $CH_{2(b')}CH_2CO_2$H acyl, H-7a,b, H-6a,b, H-15b & H-1b), 1.42 – 1.30 (m, 19H, H-5 EA, [1.40 s, 3H, CH_3 C-27 EA], [1.35 (d, *J* = 6.1 Hz, 3H, CH_3 Rha)], 6 × $CH_{2(c)}$ internal acyl), 1.18 (d, J = 12.2 Hz, 1H, H-21b EA), 1.11 – 1.03 (m, 10H, H-19b EA, [1.09 s, 3H, CH_3 C-23 EA], [1.08 s, 3H, CH_3 C-25 EA], [1.06 s, 3H, CH_3 C-24 EA]), 0.96 (s, 3H, CH_3 C-30 EA), 0.88 (s, 3H, CH_3 C-29 EA), 0.83 (s, 3H, CH_3 C-26 EA). **^{13}C NMR** (101 MHz, methanol-d_4): δ 220.7 (C-3 EA), 178.5 (CONH acyl), 177.9 (CO_2H acyl), 177.0 (CO [C-28] EA), 144.8 (C-13 EA), 123.3 (C-12 EA), 107.1 (C-1 Xyl), 101.3 (C-1 Rha), 95.5 (C-1 N-Gal), 84.3 (C-4 Rha), 78.2 (C-3 Xyl), 76.3 (C-5 N-Gal), 76.2 (C-2 Xyl), 74.9 (C-3 N-Gal), 74.6 (C-16 EA), 74.5 (C-2 N-Gal), 72.2 (C-3 Rha), 71.9 (C-2 Rha), 71.1 (C-4 Xyl), 68.9 (C-5 Rha), 67.3 (C-5 Xyl), 61.7 (C-6 N-Gal), 56.6 (C-5 EA), 52.5 (C-4 N-Gal), 50.1 (C-17 EA), 48.5 (C-4 EA), 48.0 (C-19 EA), 47.3 (C-9 EA), 42.9 (C-14 EA), 42.5 (C-18 EA), 40.7 (C-8 EA), 40.4 (C-1 EA), 37.9 (C-10 EA), 36.8 ($CH_{2(a)}$CONH acyl), 36.5 (C-15 & C-21 EA), 35.1 ($CH_{2(a')}CO_2$H acyl & C-2 EA), 33.7 (C-7 EA), 33.4 (CH_3 C-29 EA), 32.0 (C-22 EA), 31.3 (C-20 EA), 30.6, 30.5, 30.4, 30.34, 30.26 (6 × $CH_{2(c)}$ internal acyl), 27.22 ($CH_{2(b)}CH_2$CONH acyl), 27.15 (CH_3 C-23 EA), 27.1 (CH_3 C-27 EA), 26.2 ($CH_{2(b')}CO_2$H acyl), 24.9 (CH_3 C-30 EA), 24.6 (C-11 EA), 21.9 (CH_3 C-24 EA), 20.9 (C-6 EA), 18.4 (CH_3 Rha), 17.7 (CH_3 C-26 EA), 15.8 (CH_3 C-25 EA). **HRMS (MALDI)** *m/z*: Calcd for $[C_{59}H_{95}NO_{19}Na]^+$ $[M+Na]^+$ 1144.6387, found 1144.6315.

3. Synthesis of (Oxime)Echinocystic Acid Saponin 6 [(oxime)EA]

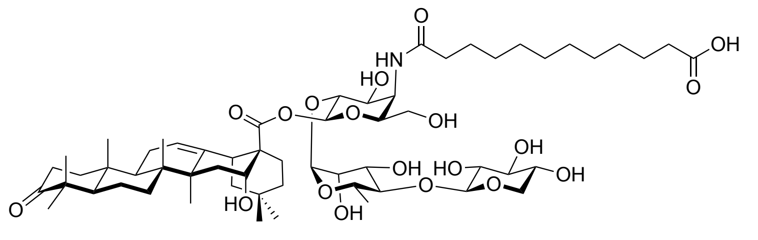

5

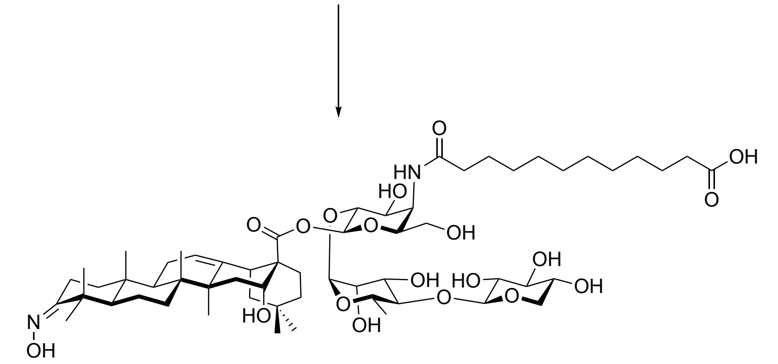

6

(Oxime)echinocystc acid saponin 6 [(oxime)EA]

(Keto)echinocystic acid saponin **5** (3.51 mg, 3.1 µmol, 1.0 equiv) was dissolved in acetonitrile/water (3:1, 1.2 mL) containing $NH_2OH{\cdot}HCl$ (2.94 mg, 42.3 µmol, 13.5 equiv) and NaOAc (5.16 mg, 62.8 µmol, 20.1 equiv). To this mixture, an excess (60 µL) of NH_2OH aq. 50% was added and the reaction was left stirring overnight at 45 ºC. RP-HPLC purification (<0.3 mL per injection) was then performed on a XBridge Prep BEH300 C18 column (5 µm, 19 × 150 mm) using a linear gradient of 35–48% acetonitrile/water over 5 min (after initial 5 min at starting conditions) at a flow rate of 17 mL/min. The fraction containing the major peak (t_R = 6.36 min) was collected and lyophilized to dryness to afford the final (oxime)EA saponin **6** (3.15 mg, 88% yield) as a white powder.

HPLC: t_R = 28.22 min (gradient = 35-100% solv. B over 30 min), λ_{max} = 194.52 nm. **^{1}H NMR** (400 MHz, methanol-d_4): δ 5.39 (d, J = 1.8 Hz, 1H, H-1 Rha), 5.35 (d, J = 7.9 Hz, 1H, H-1 N-Gal), 5.32 (t, J = 3.7 Hz, 1H, H-12 EA), 4.52 – 4.47 (m, 2H, H-1 Xyl, H-16 EA), 4.35 – 4.30 (m, 1H, H-4 N-Gal), 3.97 – 3.90 (m, 3H, H-3 & H-2 N-Gal, H-2 Rha), 3.90 – 3.77 (m, 3H, H-5a Xyl, H-5 & H-3 Rha), 3.69 (td, J = 6.5, 1.7 Hz, 1H, H-5 N-Gal), 3.60 – 3.38 (m, 4H, H-4 Rha, H-4 Xyl, H-6a,b N-Gal), 3.37 – 3.33 (m, 1H, H-3 Xyl), 3.28 – 3.16 (m, 2H, H-2 & H-5b Xyl), 3.03 – 2.91 (m, 2H, H-2a & H-18 EA), 2.40 – 2.28 (m, 3H, H-19a EA, CH_2(a)CONH acyl), 2.28 – 2.21 (m, 1H, H-2b EA), 2.19 – 2.10 (m, 2H, CH_2(a')CO_2H acyl), 1.97 – 1.90 (m, 4H, H-11a,b, H-22a & H-21a EA), 1.85 – 1.73 (m, 2H, H-22b & H-1a EA), 1.73 – 1.55 (m, 7H, H-15a, H-9 & H-6a EA, $CH_{2(b)}CH_2CONH$ & $CH_{2(b')}CH_2CO_2H$ acyl), 1.54 – 1.41 (m, 4H, H-6b, H-7a,b & H-15b EA), 1.41 – 1.26 (m, 18H, [1.38 s, 3H, CH_3 C-27 EA], CH_3 Rha, 6 × $CH_{2(c)}$ internal acyl), 1.22 – 1.12 (m, 4H, [1.15 s, 3H, CH_3 C-23 EA], H-21b EA), 1.14 – 1.02 (m, 9H, [1.06 s, 6H, CH_3 C-25 & CH_3 C-24 EA], H-1b, H-19b & H-5 EA), 0.95 (s, 3H, CH_3 C-

30 EA), 0.88 (s, 3H, CH_3 C-29 EA), 0.81 (s, 3H, CH_3 C-26 EA). **^{13}C NMR** (101 MHz, methanol-d_4): δ 183.1 (CO_2H acyl), 178.5 (CONH acyl), 177.1 (CO [C-28] EA), 167.3 (CNOH), 144.7 (C-13 EA), 123.4 (C-12 EA), 107.1 (C-1 Xyl), 101.4 (C-1 Rha), 95.6 (C-1 N-Gal), 84.3 (C-4 Rha), 78.2 (C-3 Xyl), 76.4 (C-5 N-Gal), 76.2 (C-2 Xyl), 74.8 (C-3 N-Gal), 74.7 (C-16 EA), 74.6 (C-2 N-Gal), 72.2 (C-3 Rha), 71.9 (C-2 Rha), 71.1 (C-4 Xyl), 68.9 (C-5 Rha), 67.3 (C-5 Xyl), 61.7 (C-6 N-Gal), 57.3 (C-5 EA), 52.5 (C-4 N-Gal), 50.1 (C-17 EA), 48.0 (C-19 EA), 47.6 (C-9 EA), 42.8 (C-14 EA), 42.4 (C-18 EA), 41.0 (C-4 EA), 40.8 (C-8 EA), 39.7 (C-1 EA), 39.3 ($\mathit{C}H_{2(a)}$CONH acyl), 38.2 (C-10 EA), 36.8 ($\mathit{C}H_{2(a')}CO_2H$ acyl), 36.5 (C-15 & C-21 EA), 34.0 (C-7 EA), 33.4 (CH_3 C-29 EA), 32.0 (C-22 EA), 31.3 (C-20 EA), 30.9, 30.7, 30.6, 30.5, 30.4 (6 × $CH_{2(c)}$ internal acyl), 28.2 (CH_3 C-23 EA), 27.8 ($\mathit{C}H_{2(b')}CO_2H$ acyl), 27.3 ($\mathit{C}H_{2(b)}CH_2$CONH acyl), 27.1 (CH_3 C-27 EA), 24.9 (CH_3 C-30 EA), 24.6 (C-11 EA), 23.9 (CH_3 C-24 EA), 20.4 (C-6 EA), 18.4 (CH_3 Rha), 17.9 (C-2 EA), 17.8 (CH_3 C-26 EA), 15.7 (CH_3 C-25 EA). **HRMS (MALDI)** *m/z*: Calcd for $[C_{59}H_{96}N_2O_{19}Na]^+$ $[M+Na]^+$ 1159.6496, found 1159.6463.

IV. IMMUNOLOGICAL EVALUATION IN MICE

Animals. Animals were cared for and handled in compliance with the Guidelines for Accommodation and Care of Animals (European Convention for the Protection of Vertebrate Animals Used for Experimental and Other Scientific Purposes) and internal guidelines. Mice were housed in ventilated cages and fed on a standard diet *ad libitum*. All the experimental procedures were approved by the appropriate local authorities. The CIC bioGUNE animal facility is fully accredited by AAALAC International.

Vaccination of mice. Groups of five mice (C57BL/6, female, 6-8 weeks old) were vaccinated subcutaneously three times every 10 days (days 0, 11, and 21) with endotoxin-free OVA (EndoFit™ Ovalbumin, Invitrogen) (10 µg/mouse) in phosphate-buffered saline (PBS, 100 µL) either alone (without adjuvant) or with the synthetic saponins (50 µg/mouse). To analyze the antibody responses over time, mice were bled via the submandibular vein at the indicated pre- (day –1) and post-vaccination timepoint (day 18) and by cardiac puncture at the experimental endpoint (day 28). Blood was collected in BD Microtainer® tubes (Clot Activator/SST™ Gel) and centrifuged at 7500*g* for 10 min, after which serum was harvested and stored at –20 °C until further analysis.

Evaluation of immune response augmentation. Analysis of the produced plasma antibodies specific against OVA was performed by an indirect enzyme-linked immunosorbent assay (ELISA). Briefly, ELISA plates (Thermo Scientific) were coated with endotoxin-free OVA (EndoFit™ Ovalbumin; Invitrogen) at 0.05 µg/well in carbonate buffer (pH 9.5) and plates were incubated overnight at 4 °C. After washing the wells (PBS, 10 mM, containing 0.05% Tween 20), plates were blocked with 10% of fetal calf serum (FCS, Biowest) in PBS buffer for 1 h. Serial dilutions of mouse sera in blocking buffer (10% FCS in PBS buffer) were added to wells with appropriate controls and incubated for 1 h at room temperature. After washing, goat anti-mouse total IgG (Jackson ImmunoResearch) or subclass-specific IgG1, IgG2b, and IgG2c (SouthernBiotech) antibodies conjugated to horseradish peroxidase (HRP) were added to each well. Antibodies were diluted as indicated by the manufacturer, i.e. IgG at 1/5000; IgG1 and IgG2b at 1/4000 dilution; and IgG2c at 1/10000. After 1 h incubation at room temperature, KPL SureBlue reserve™ commercial solution (100 µL/well, SeraCare) containing 3,3',5,5'-tetramethylbenzidine (TMB) was added as peroxidase substrate. The reaction was stopped after 10 min incubation by adding 2N H_2SO_4 (100 µL/well). For absorbance measurments, optical density (OD) at 450 nm was immediately determined using a BioTek® Synergy HT multi-detection microplate reader. Antibody endpoint titres were calculated as the highest serum dilution that showed an absorbance of 0.1 or greater over that of the pre-sera, as previously reported.[3]

Initial toxicity assessment in mice. As a standard initial overall assessment of the potential toxicity of the saponins, in addition to visual inspection for potential signs of discomfort, the weight loss of each group of mice was monitored before and after each immunization. The median percentage weight change at different timepoints post-injection was determined and analyzed.

Statistics. Two-way ANOVA Dunnett's multiple comparisons test was used to assess statistical significance across the different dose-response curves at the relevant dilutions compared to saponin lead **3** EA, and to the no-adjuvant OVA-alone control, respectively. Antibody titers values defined as previously reported[2] are presented as median of five mice as black horizontal bars. When comparing two experimental groups, statistical significance of

each antibody response compared to the saponin lead **3** EA or, alternatively, to the no-adjuvant group was assessed in each case using a two-tailed unpaired Student's *t*-test with a confidence interval CI = 95% (GraphPad Prism, GraphPad Software, La Jolla, CA). * $p \leq 0.05$, ** $p \leq 0.01$, *** $p \leq 0.001$, **** $p \leq 0.0001$.

V. NMR CHARACTERIZATION SPECTRA: ^{1}H, APT ^{13}C, COSY, HSQC

1. (OXIME)QUILLAIC ACID SAPONIN **4** [(OXIME)QA] S22
2. (KETO)ECHINOCYSTIC ACID SAPONIN **5** [(KETO)EA] S26
3. (OXIME)ECHINOCYSTIC ACID SAPONIN **6** [(OXIME)EA] S58

4 ^{1}H-NMR (400 MHz, methanol-d_4)

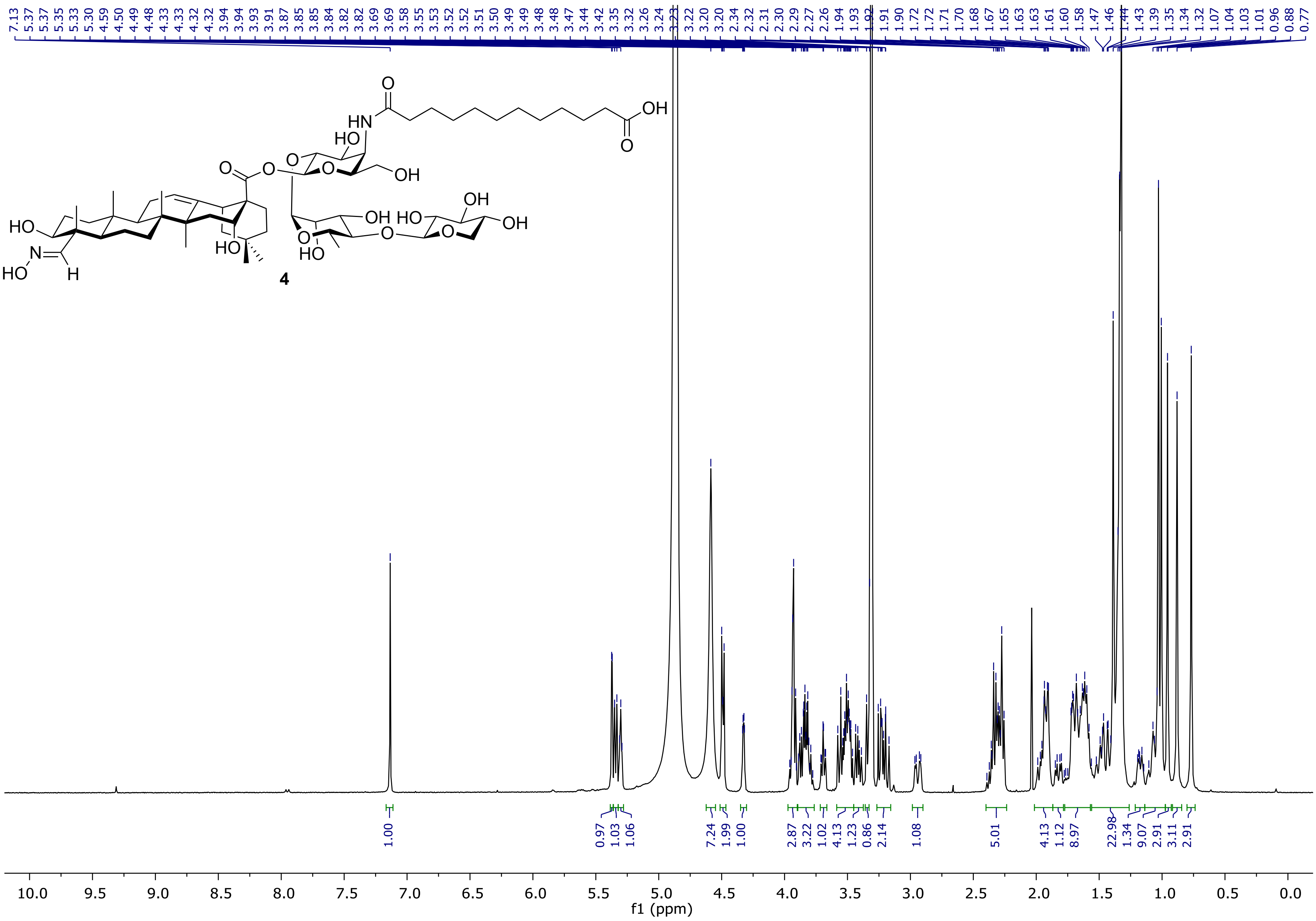

4 APT ^{13}C-NMR (101 MHz, methanol-d_4).

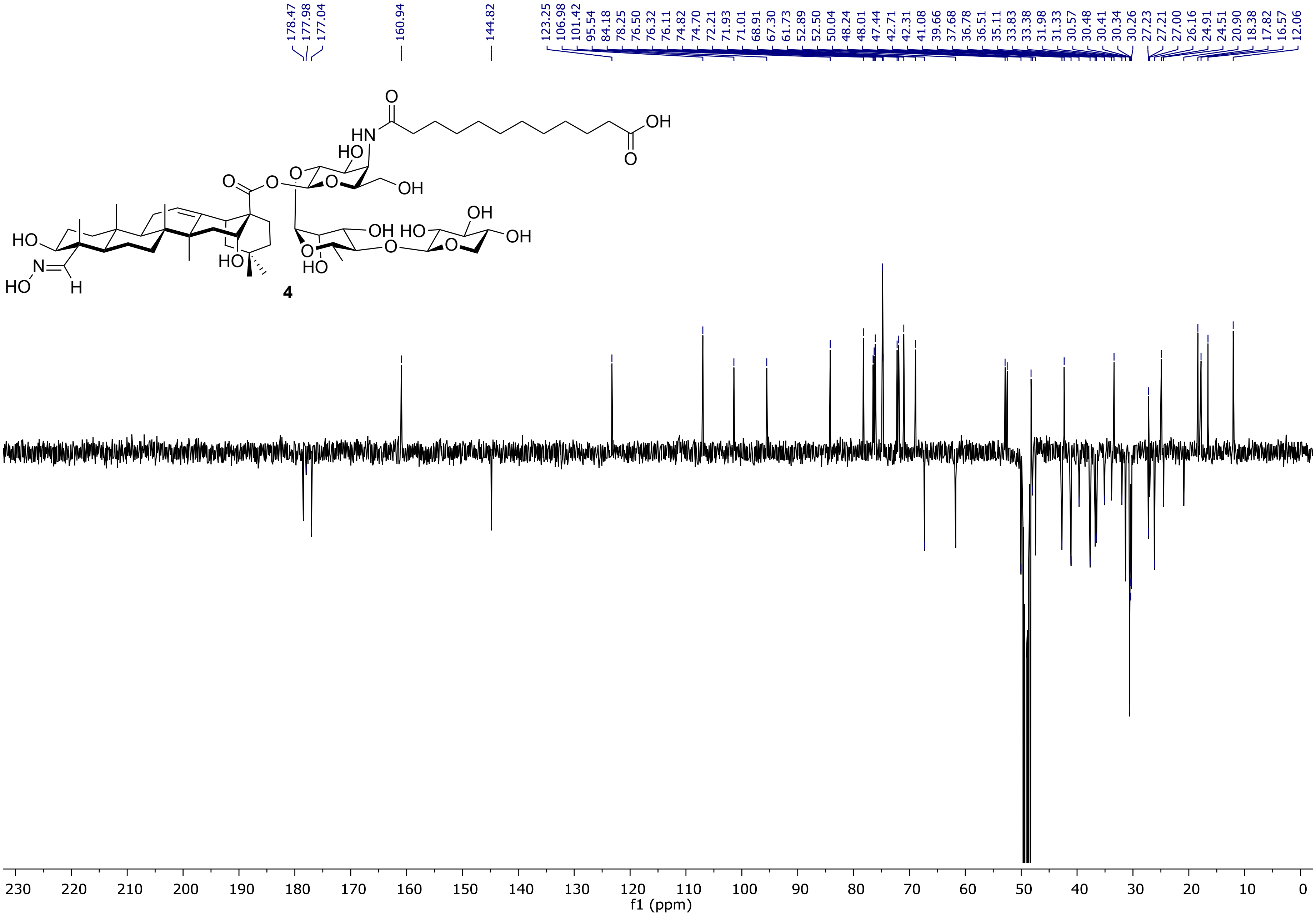

4

4 ^1H-^1H COSY NMR (400 MHz, methanol-d_4).

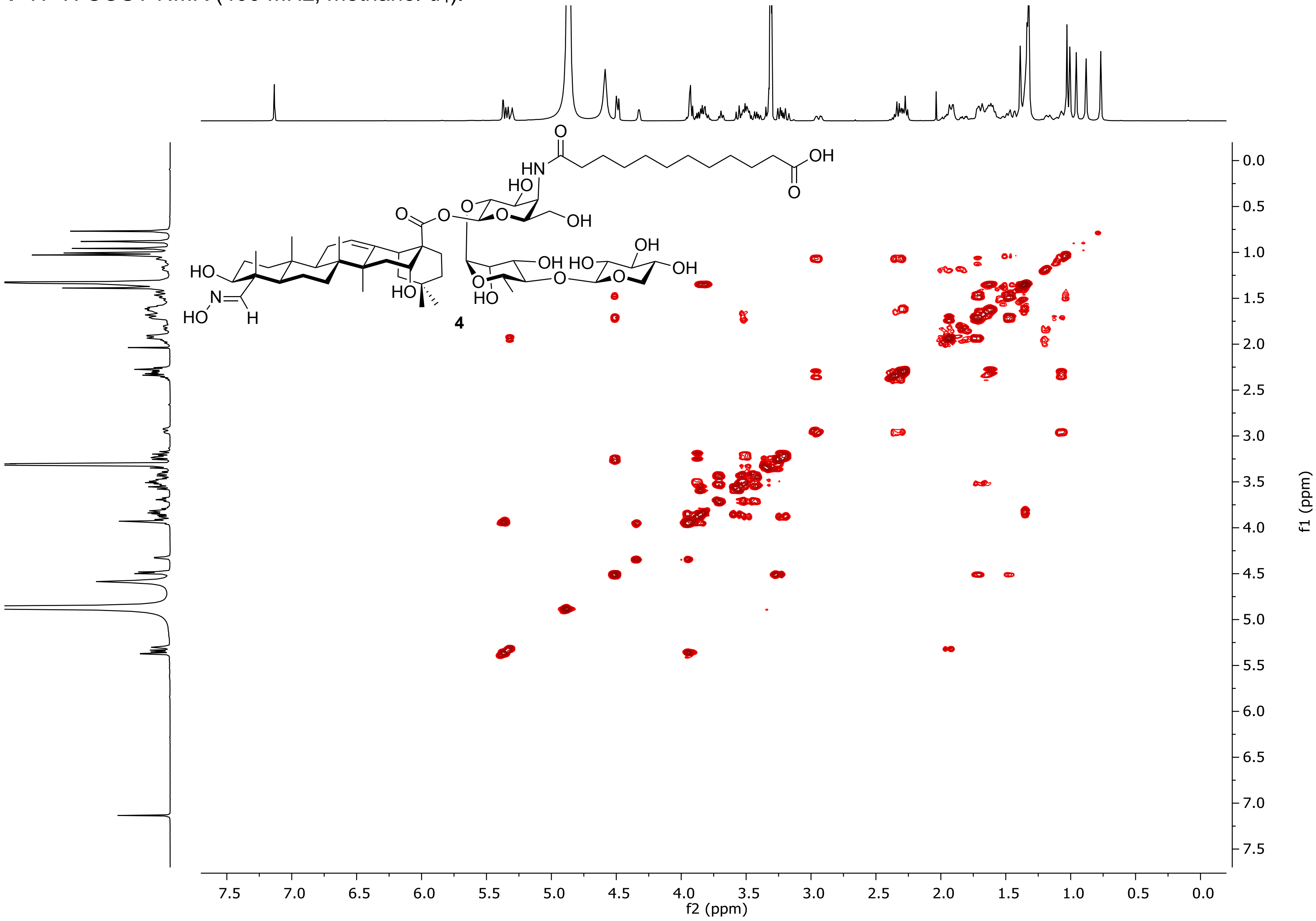

4 ^{1}H-^{13}C HSQC NMR (400 MHz, methanol-d_4)

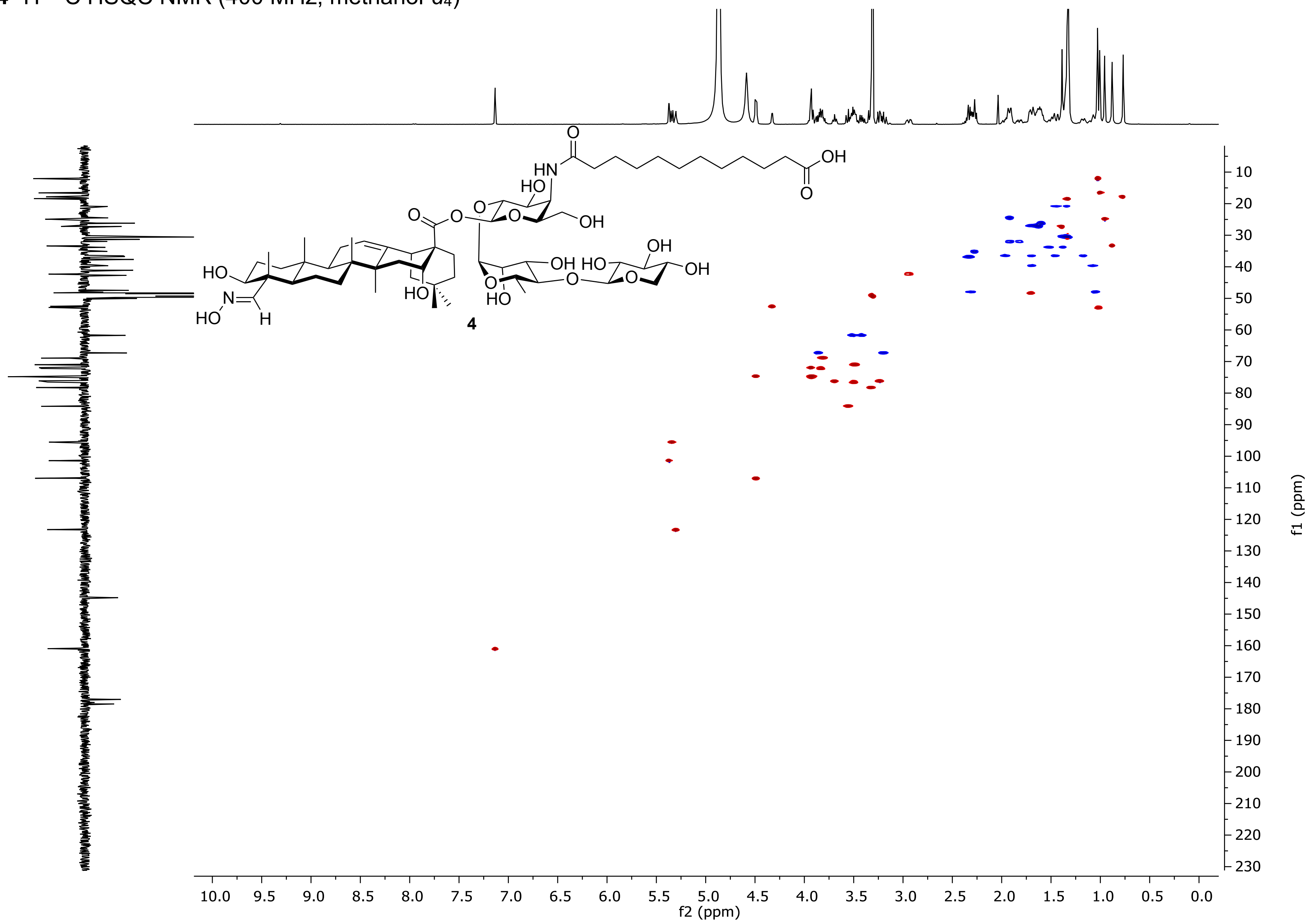

7 ^{1}H-NMR (600 MHz, $CDCl_3$)

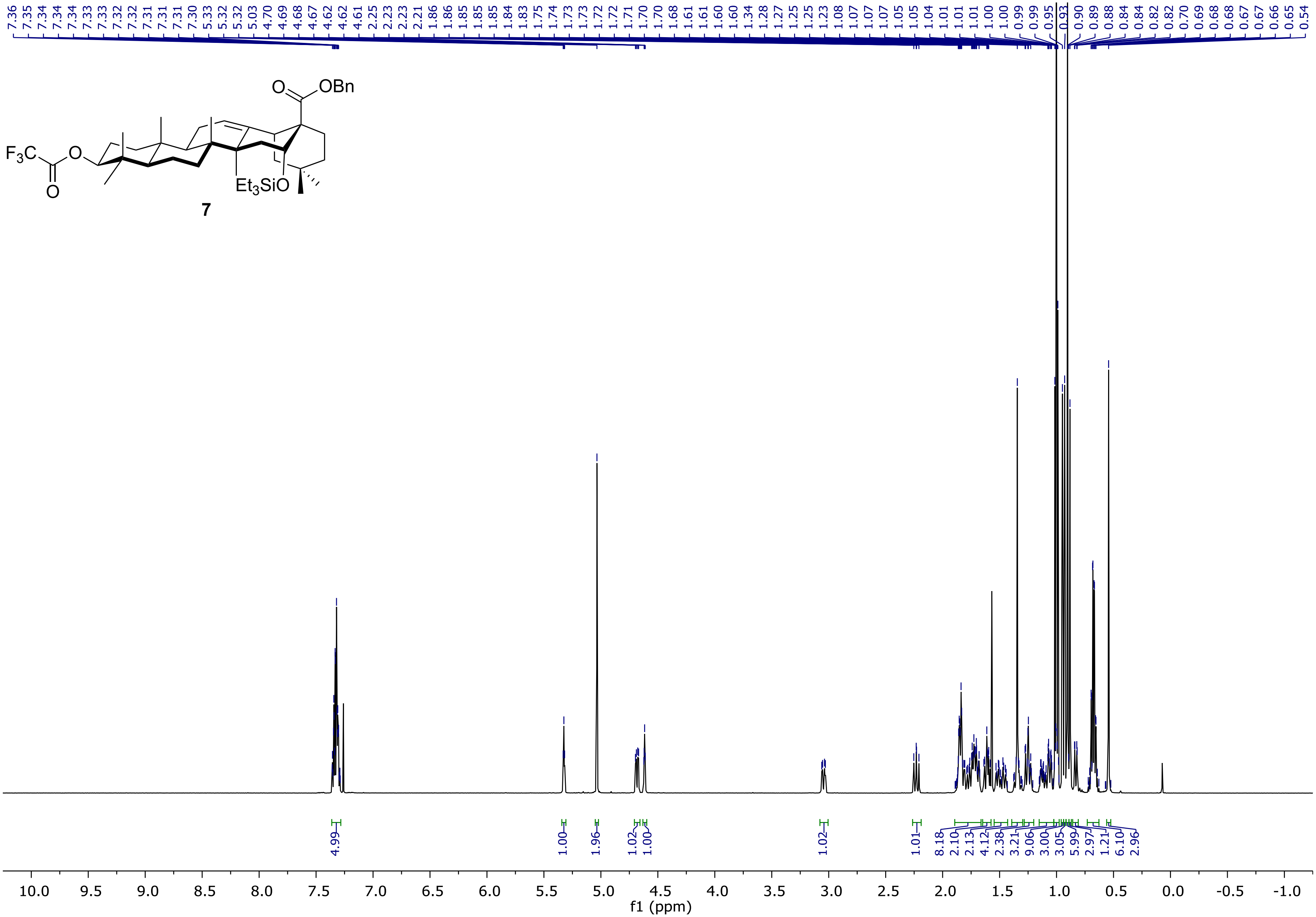

7 APT ^{13}C-NMR (151 MHz, $CDCl_3$)

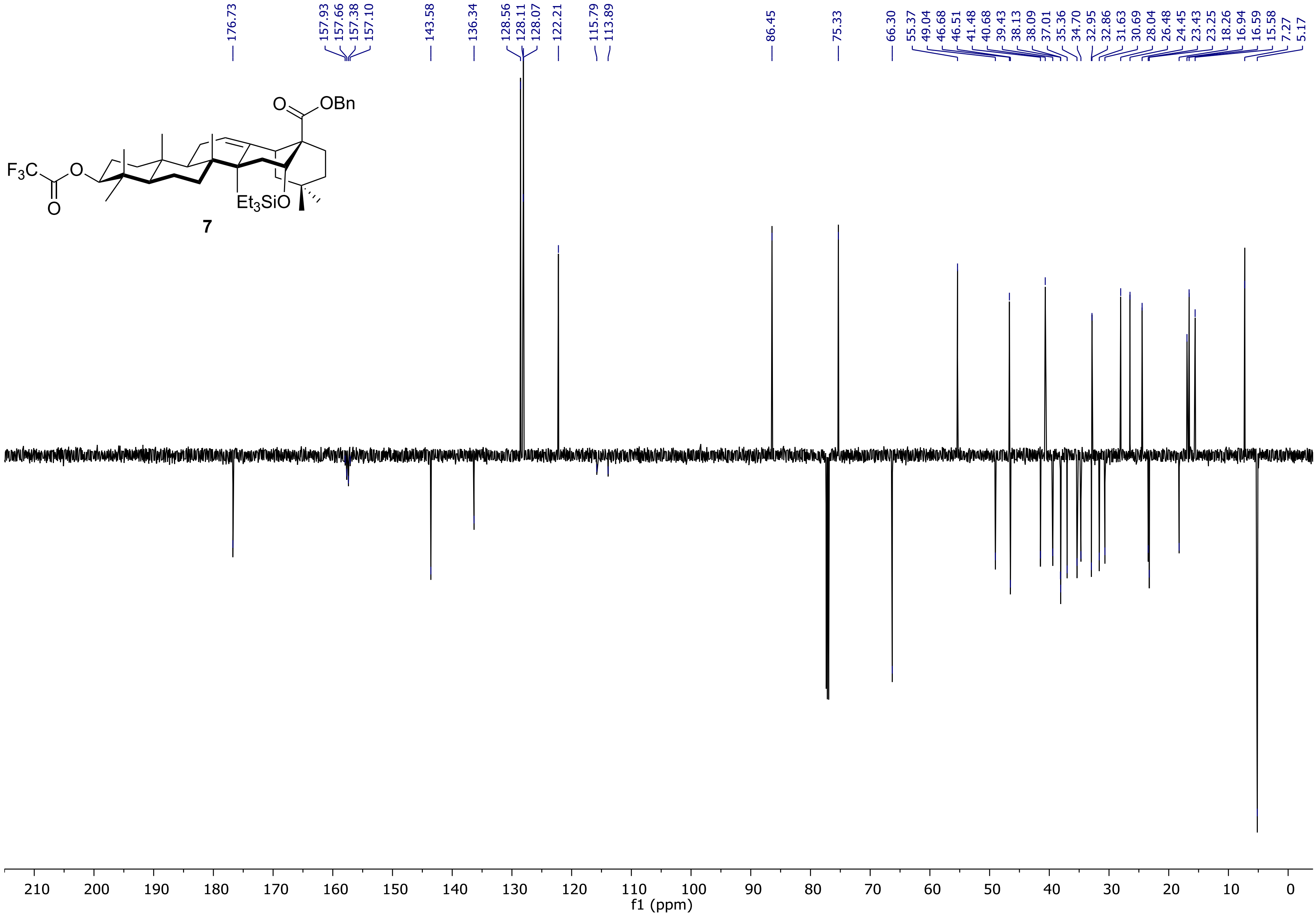

7 ^{1}H-^{1}H COSY (600 MHz, $CDCl_3$)

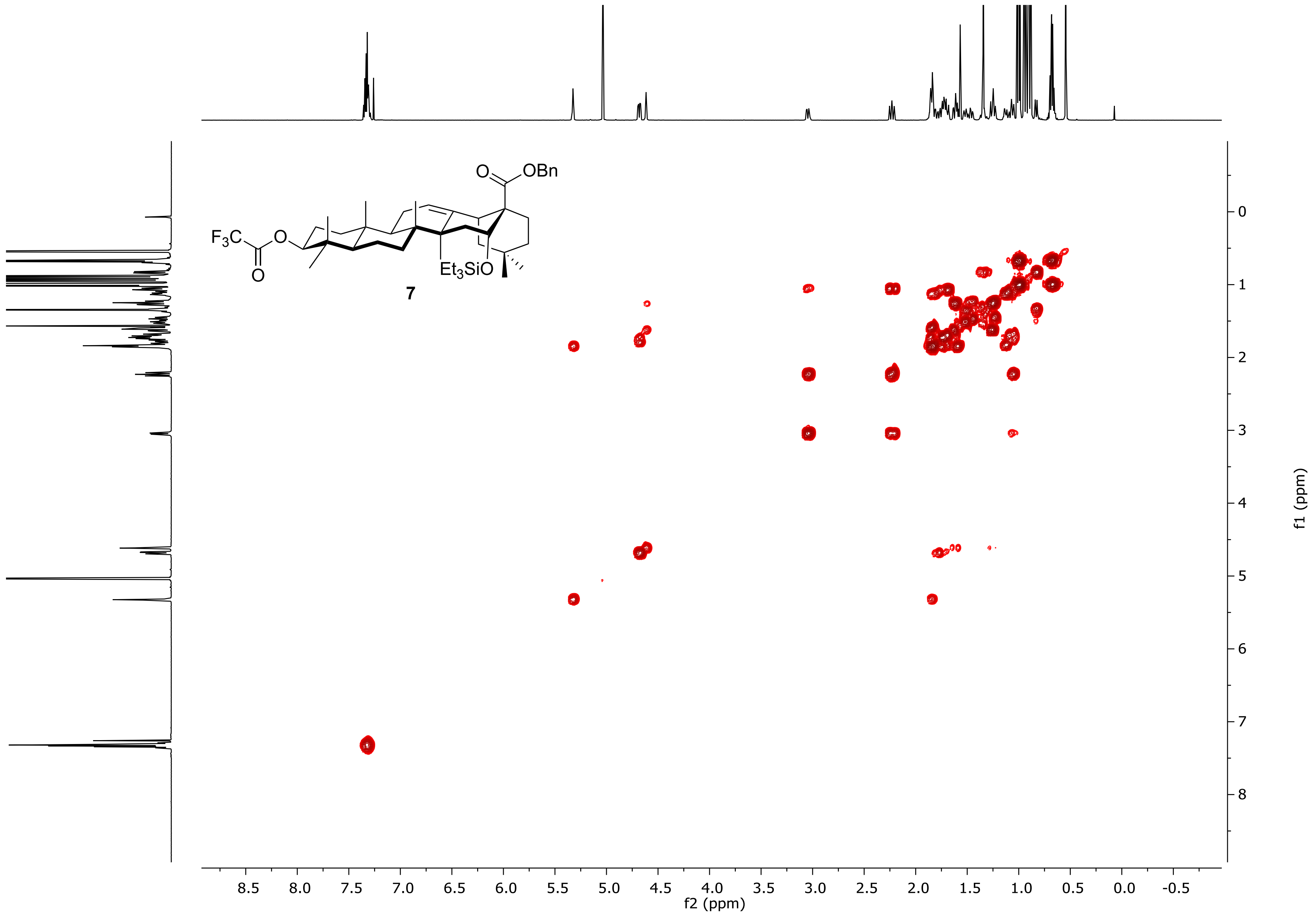

7 ^{1}H-^{13}C HSQC (600 MHz, $CDCl_3$)

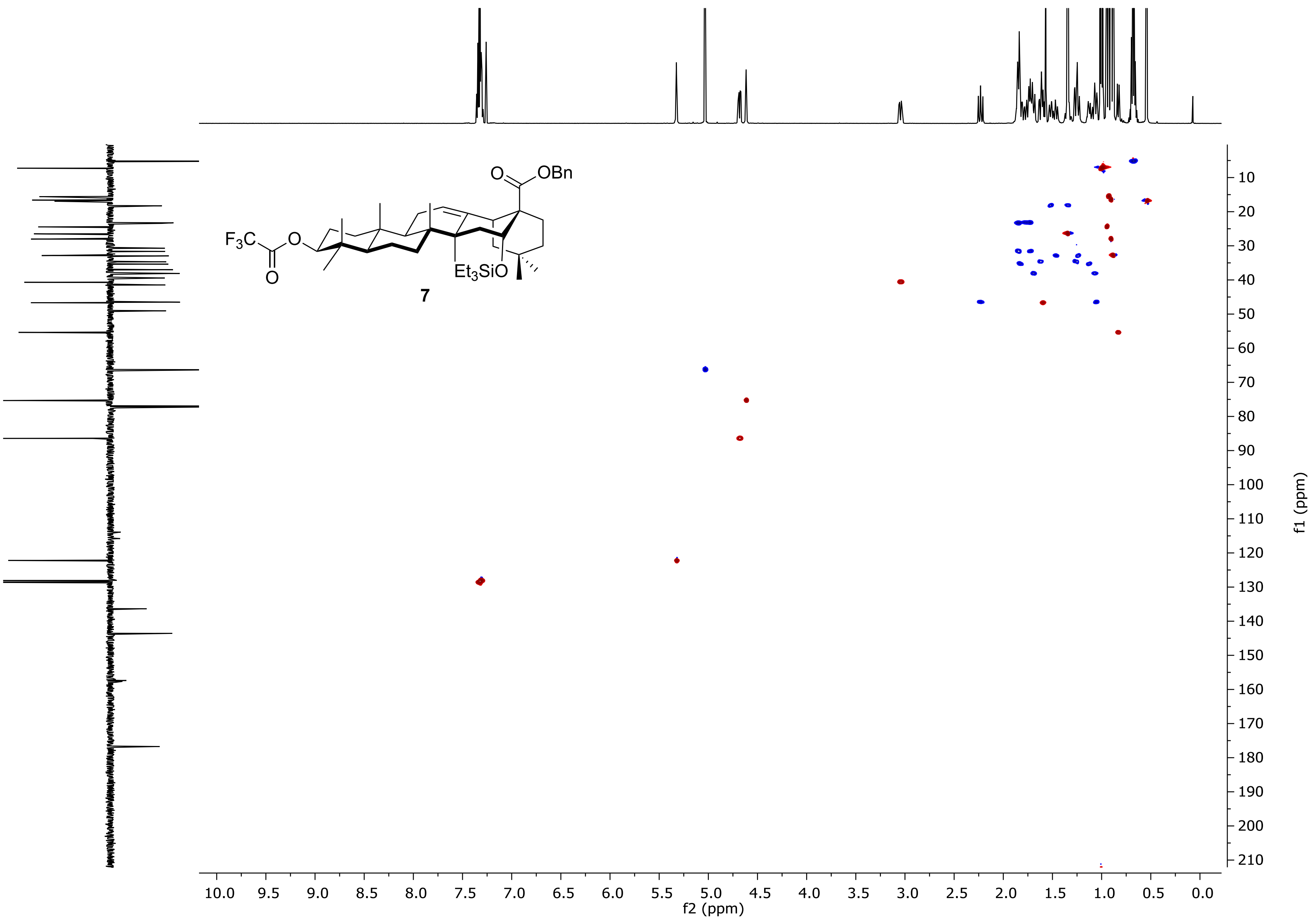

S2 ^{1}H-NMR (600 MHz, $CDCl_3$)

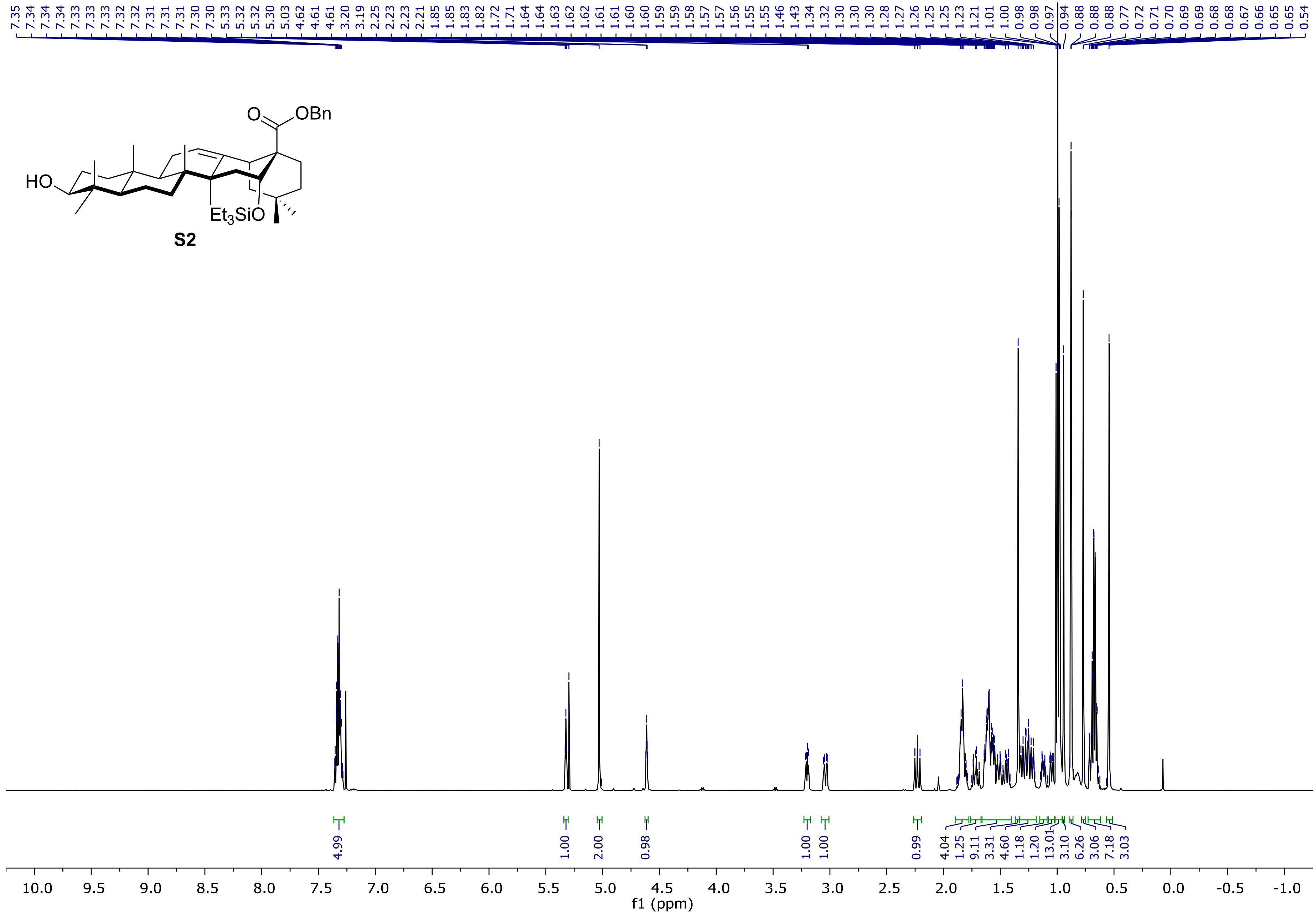

S2 APT ^{13}C-NMR (151 MHz, $CDCl_3$)

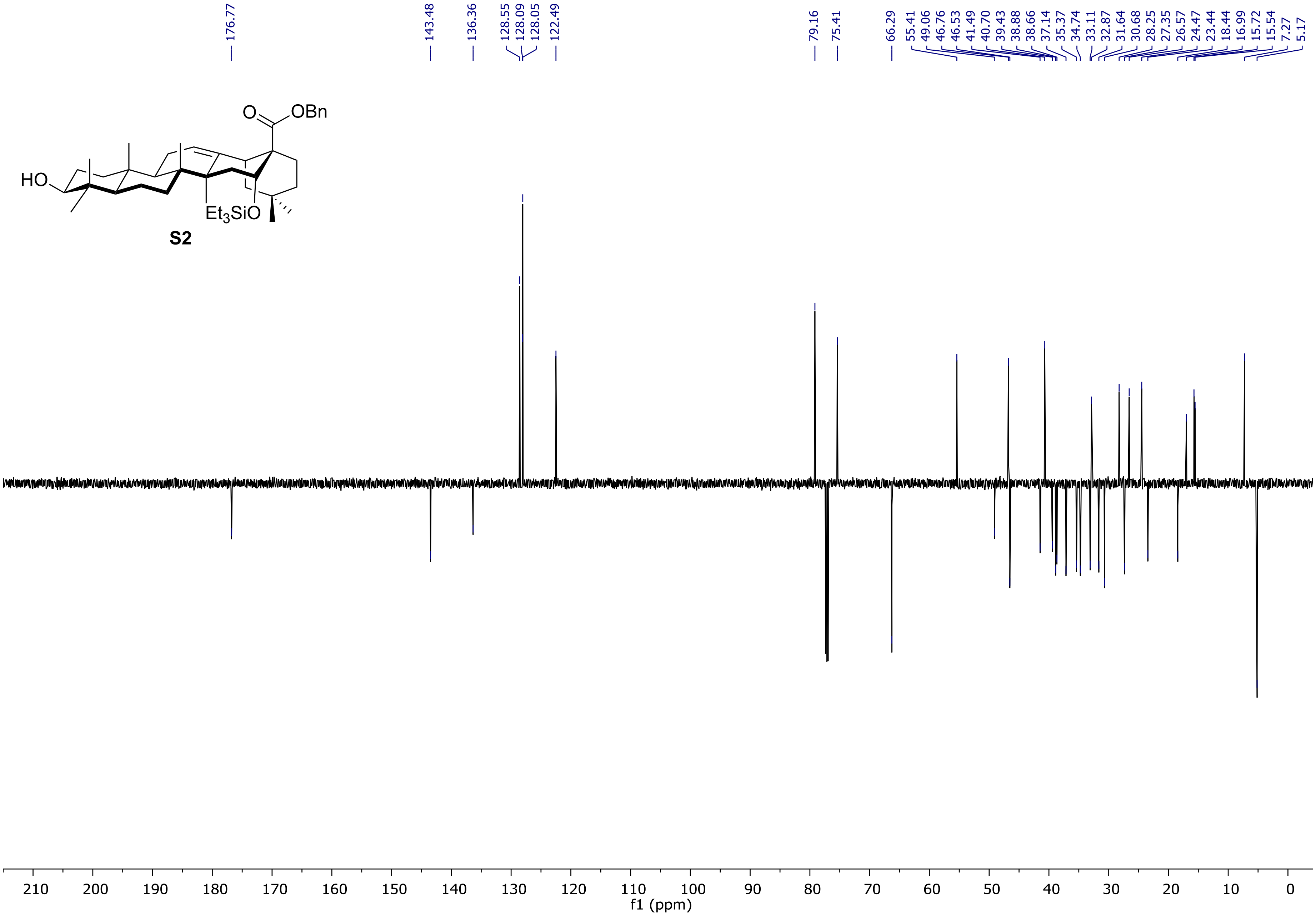

S2 ^{1}H-^{1}H COSY (600 MHz, $CDCl_3$)

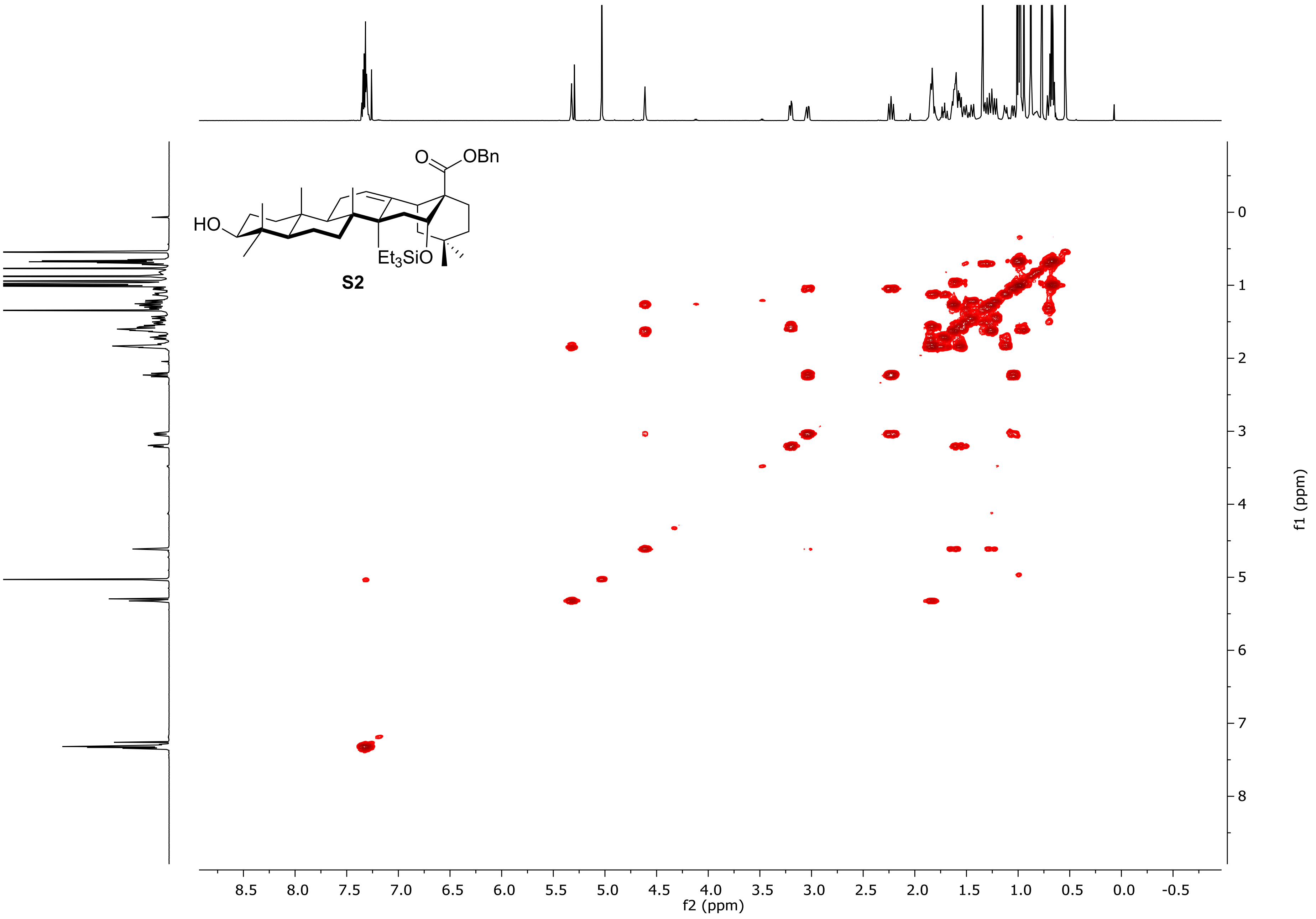

S2 ^{1}H-^{13}C HSQC (600 MHz, $CDCl_3$)

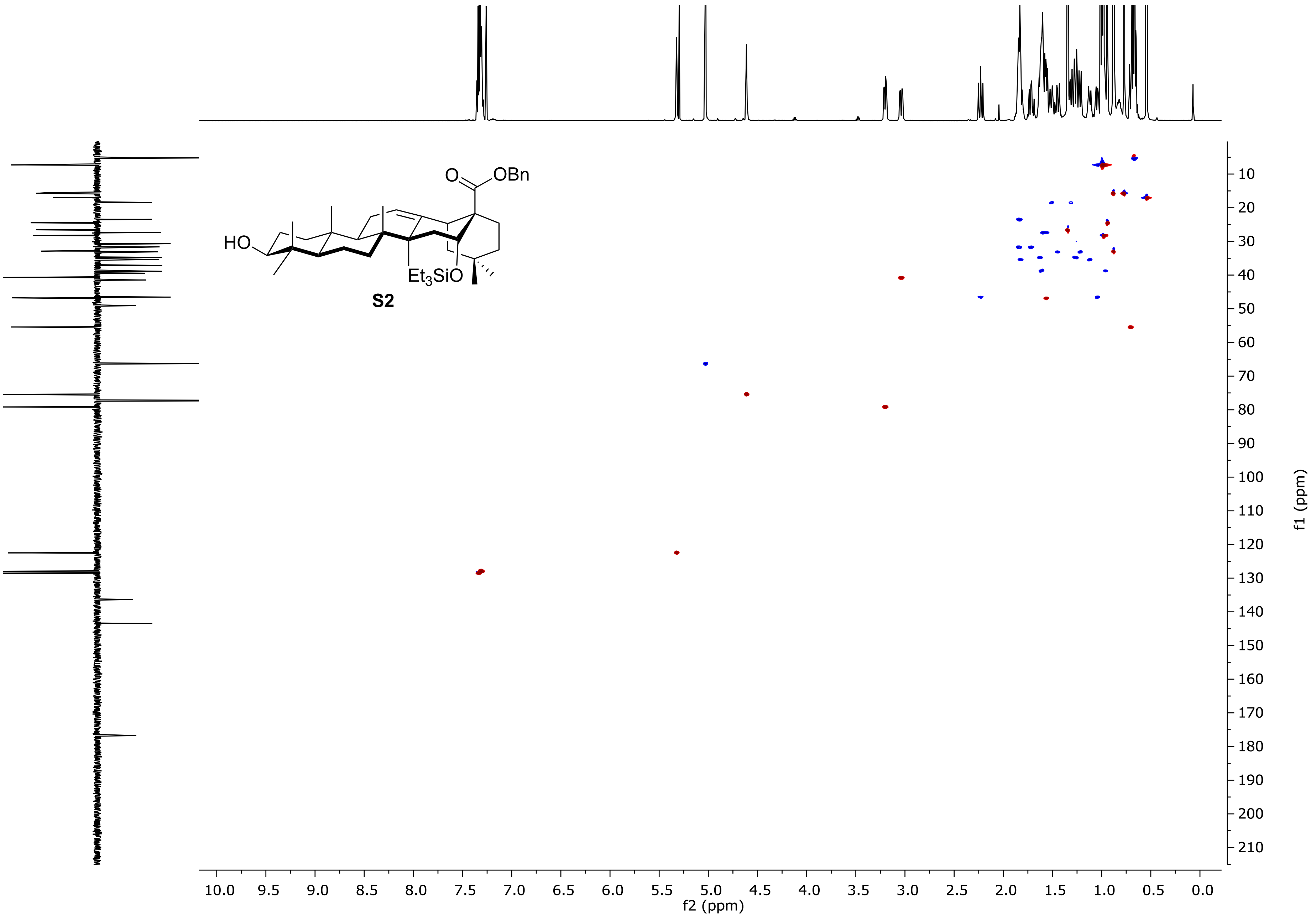

S3 ^{1}H-NMR (600 MHz, $CDCl_3$)

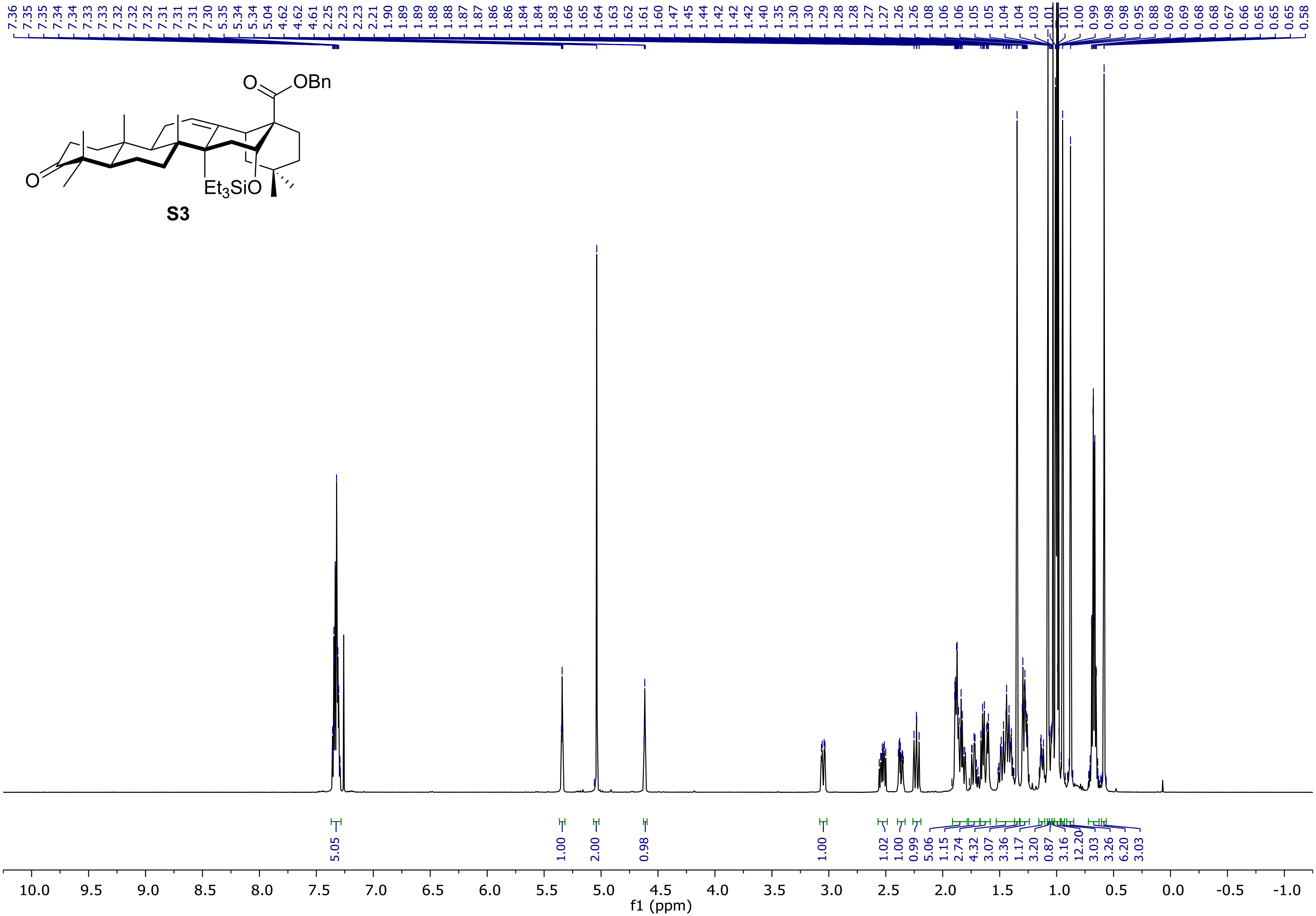

S3 APT ^{13}C-NMR (151 MHz, $CDCl_3$)

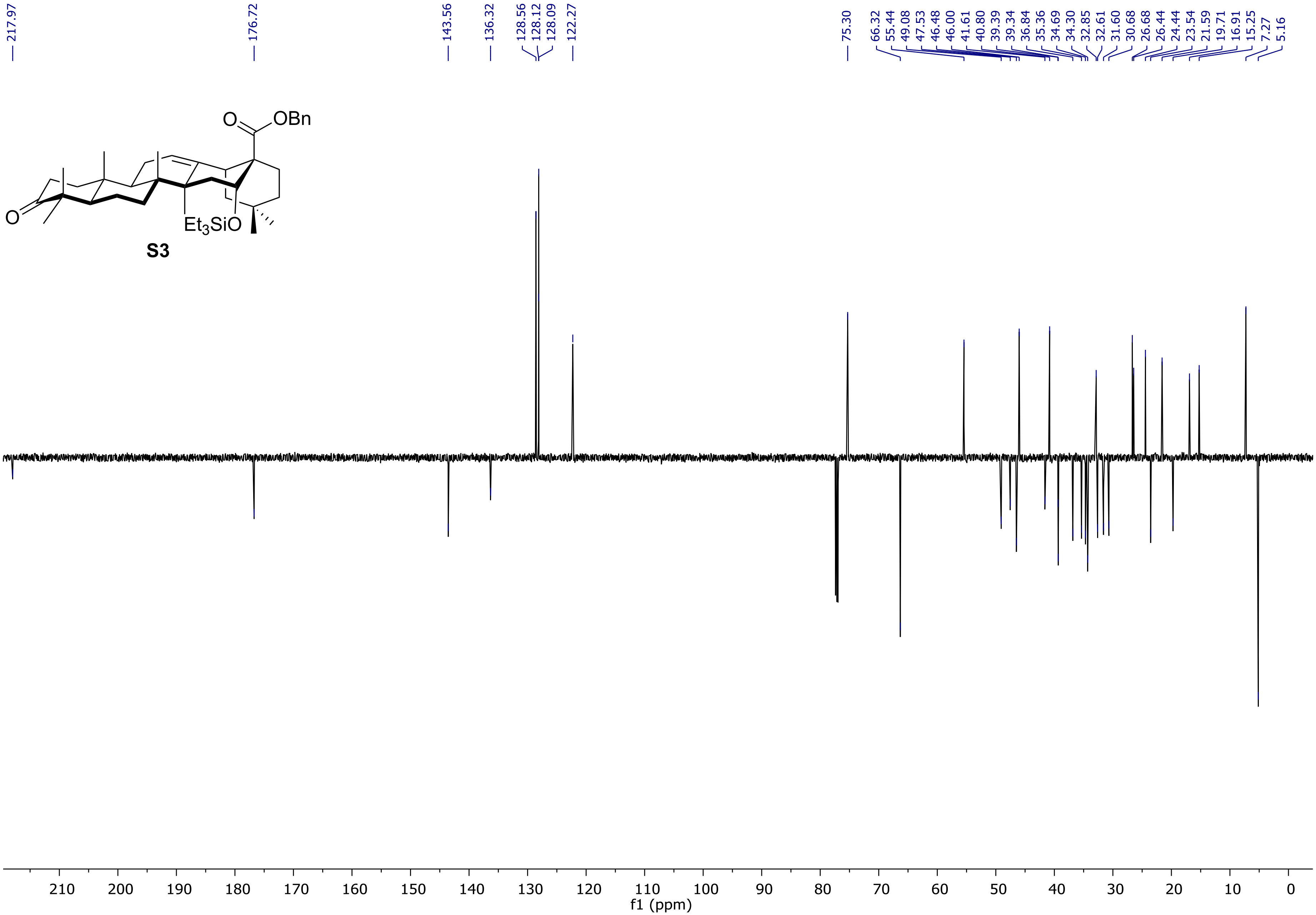

S3 ^{1}H-^{1}H COSY (600 MHz, $CDCl_3$)

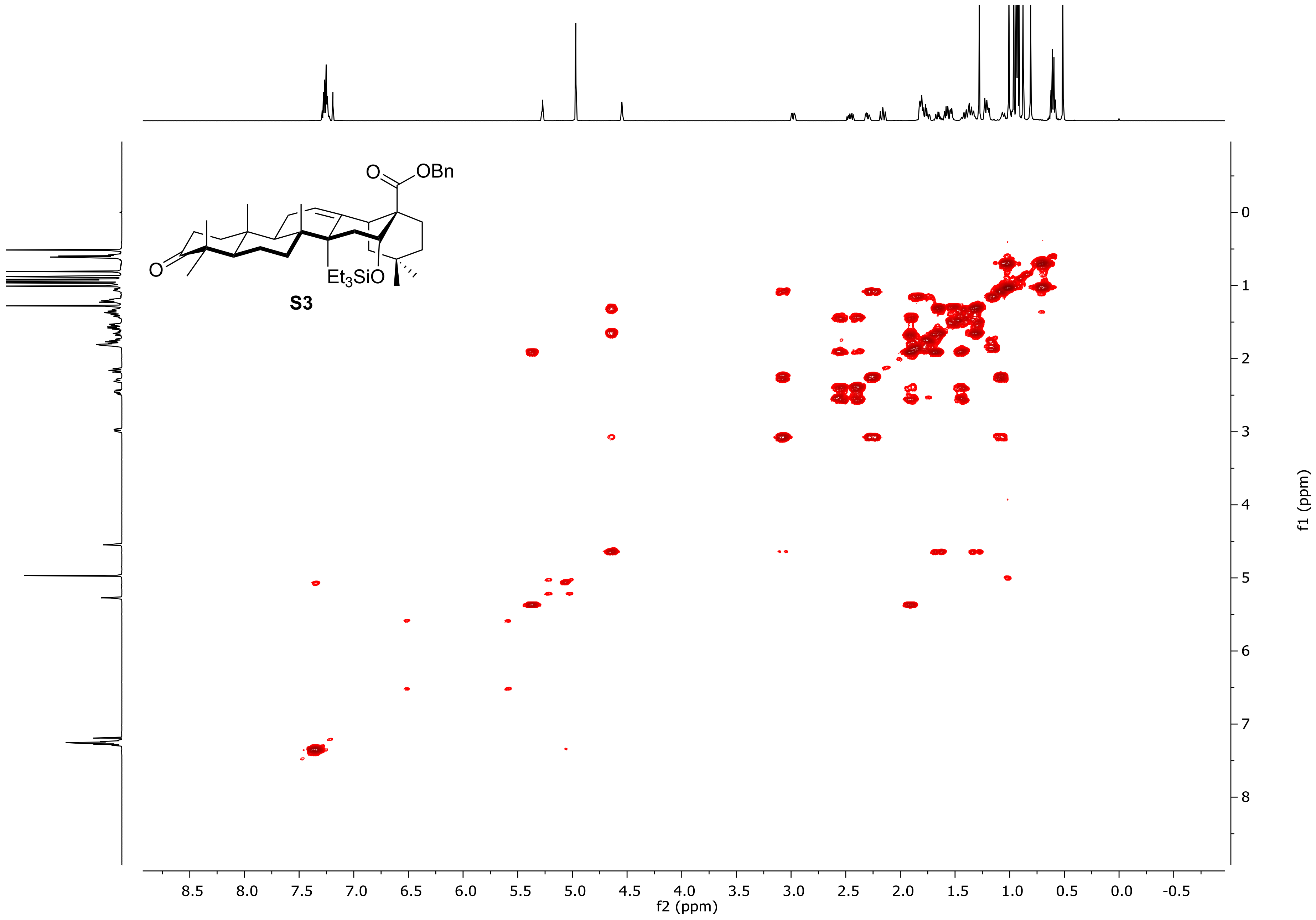

S3 ^{1}H-^{13}C HSQC (600 MHz, $CDCl_3$)

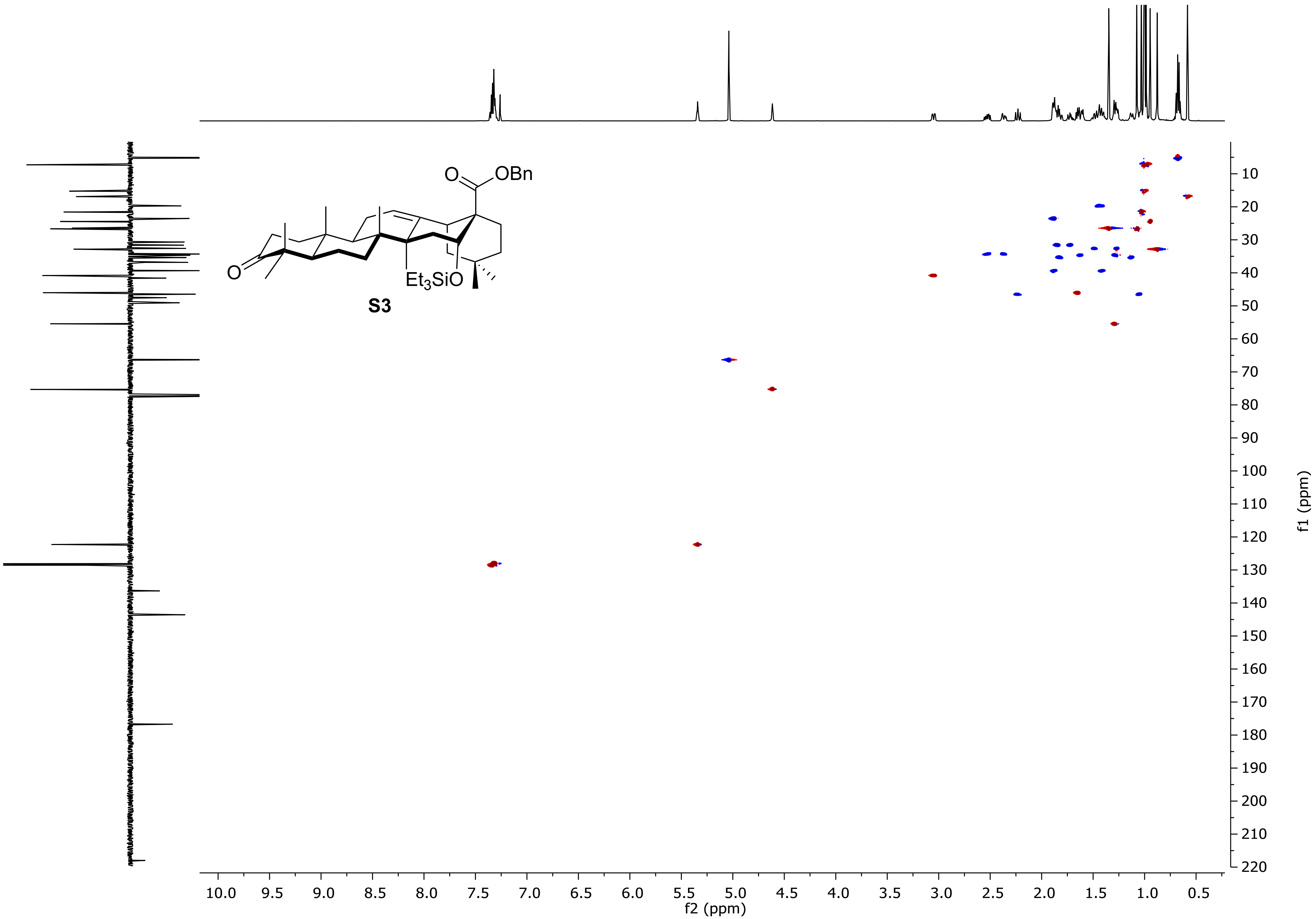

8 ^{1}H-NMR (600 MHz, $CDCl_3$)

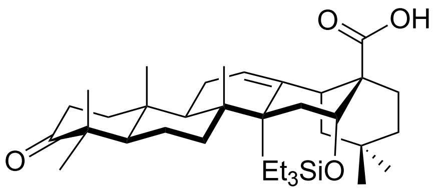

8

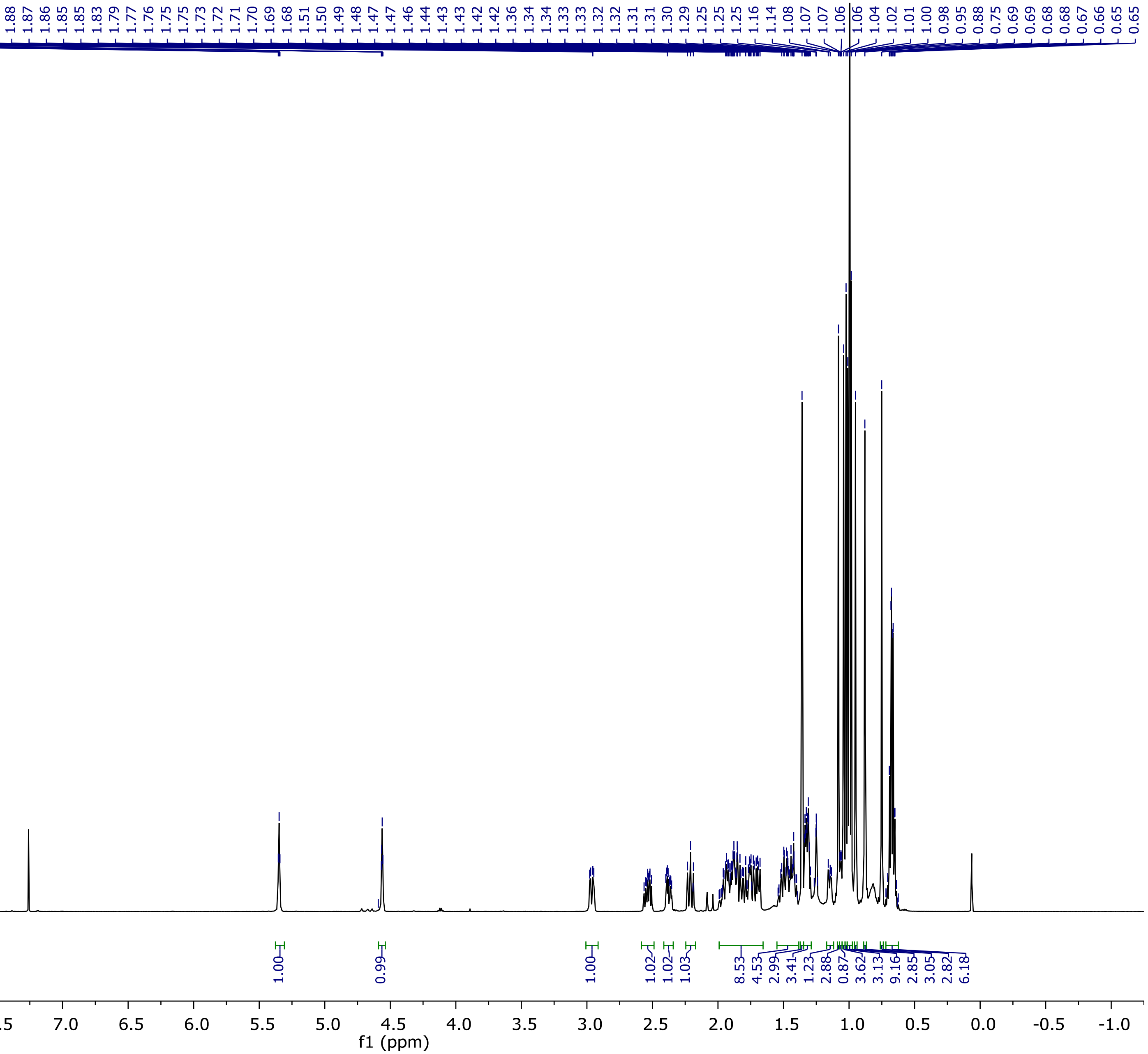

8 APT ^{13}C-NMR (151 MHz, $CDCl_3$)

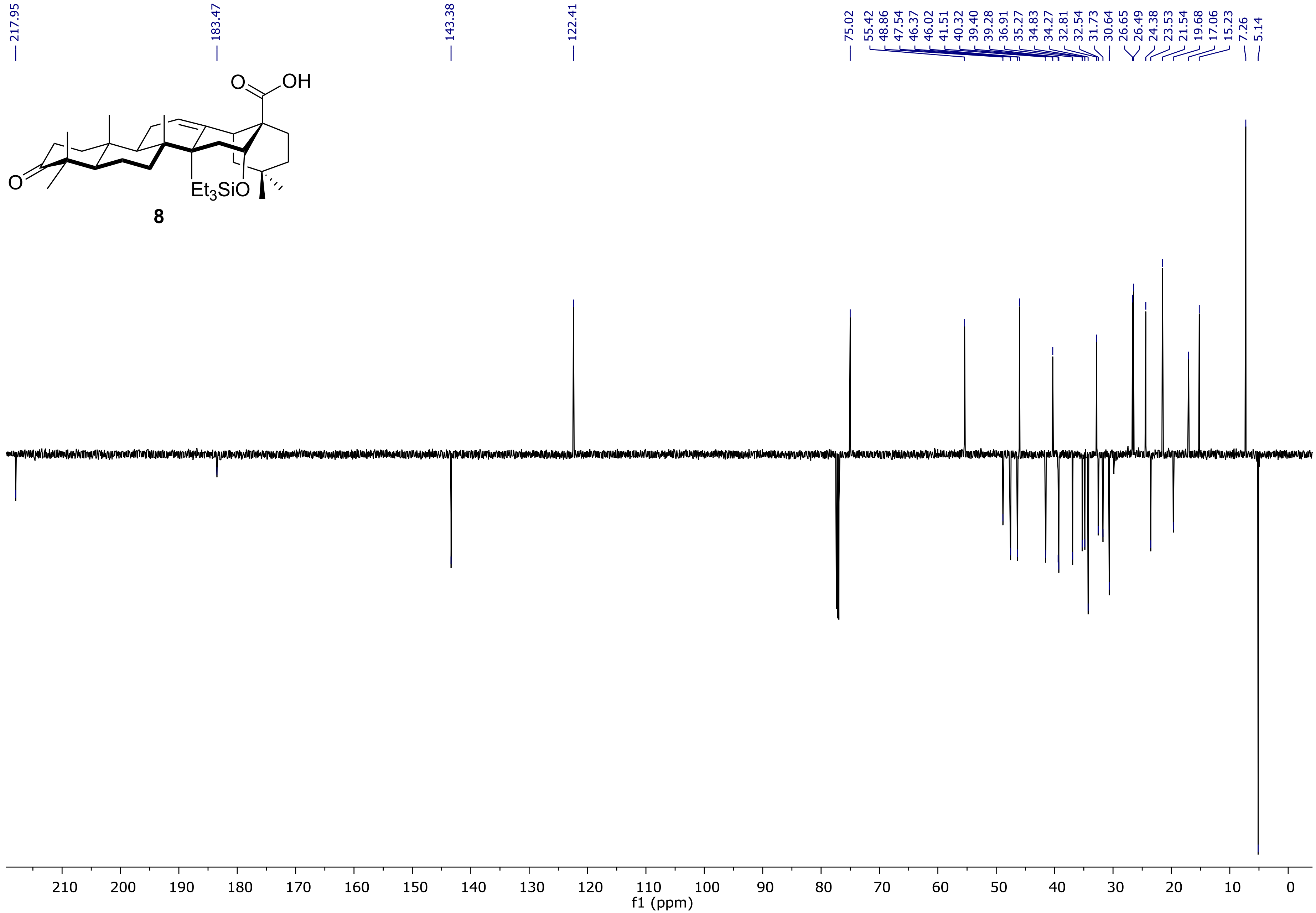

8 ^{1}H-^{1}H COSY (600 MHz, $CDCl_3$)

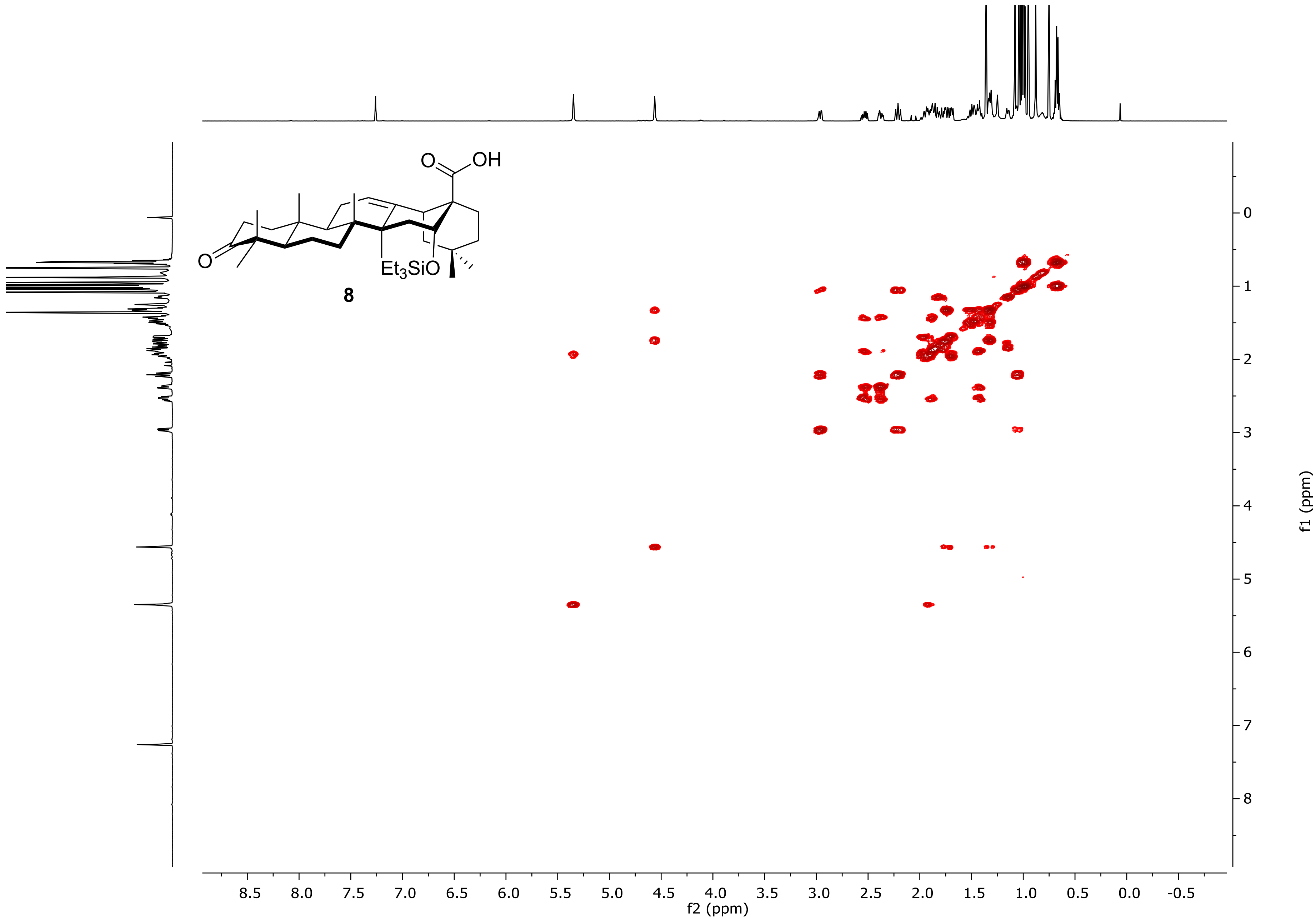

8 ^1H-^{13}C HSQC (600 MHz, $CDCl_3$)

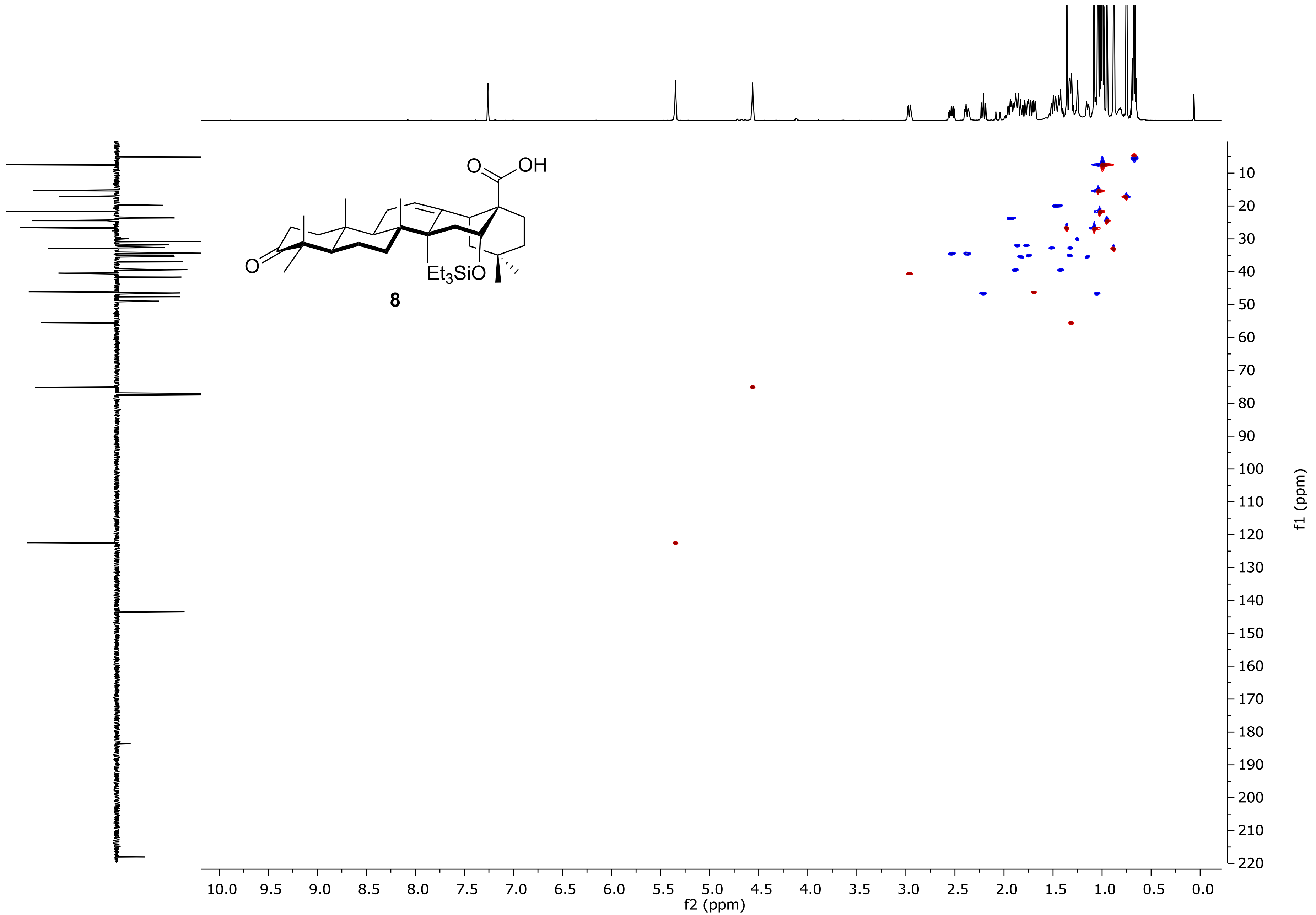

S4 ^{1}H-NMR (600 MHz, $CDCl_3$)

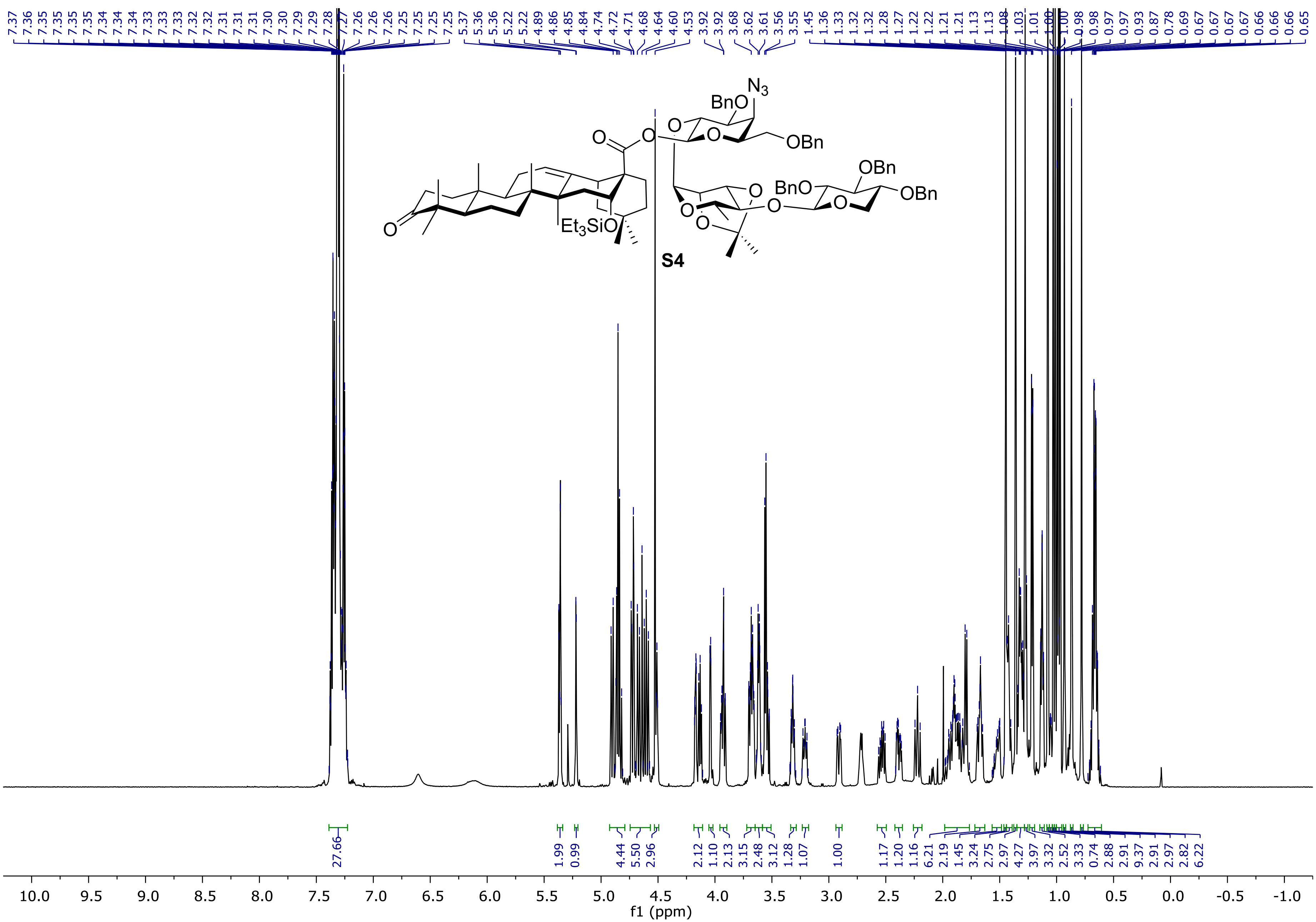

S4 APT ^{13}C-NMR (151 MHz, $CDCl_3$)

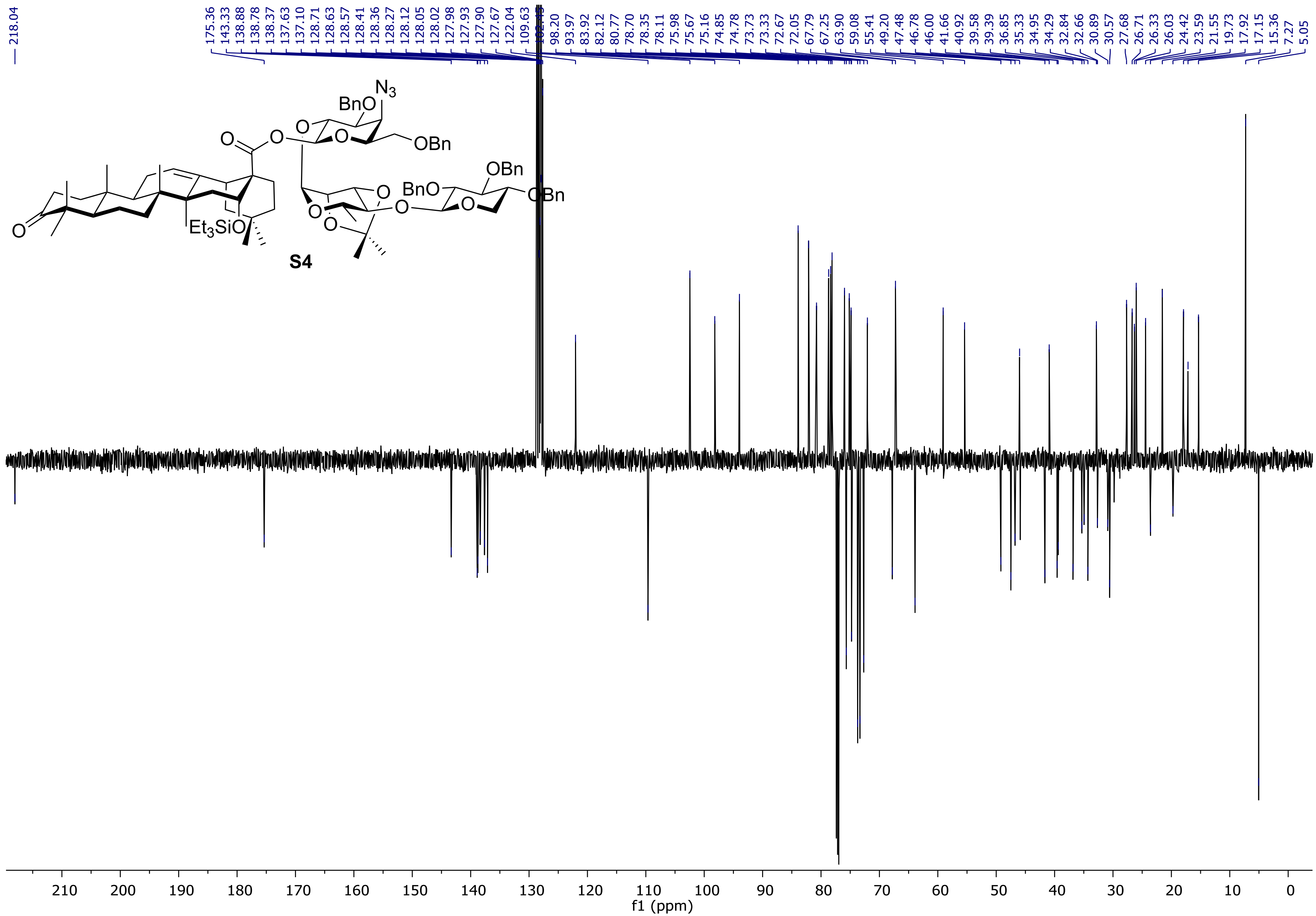

S4 ^1H-^1H COSY (600 MHz, $CDCl_3$)

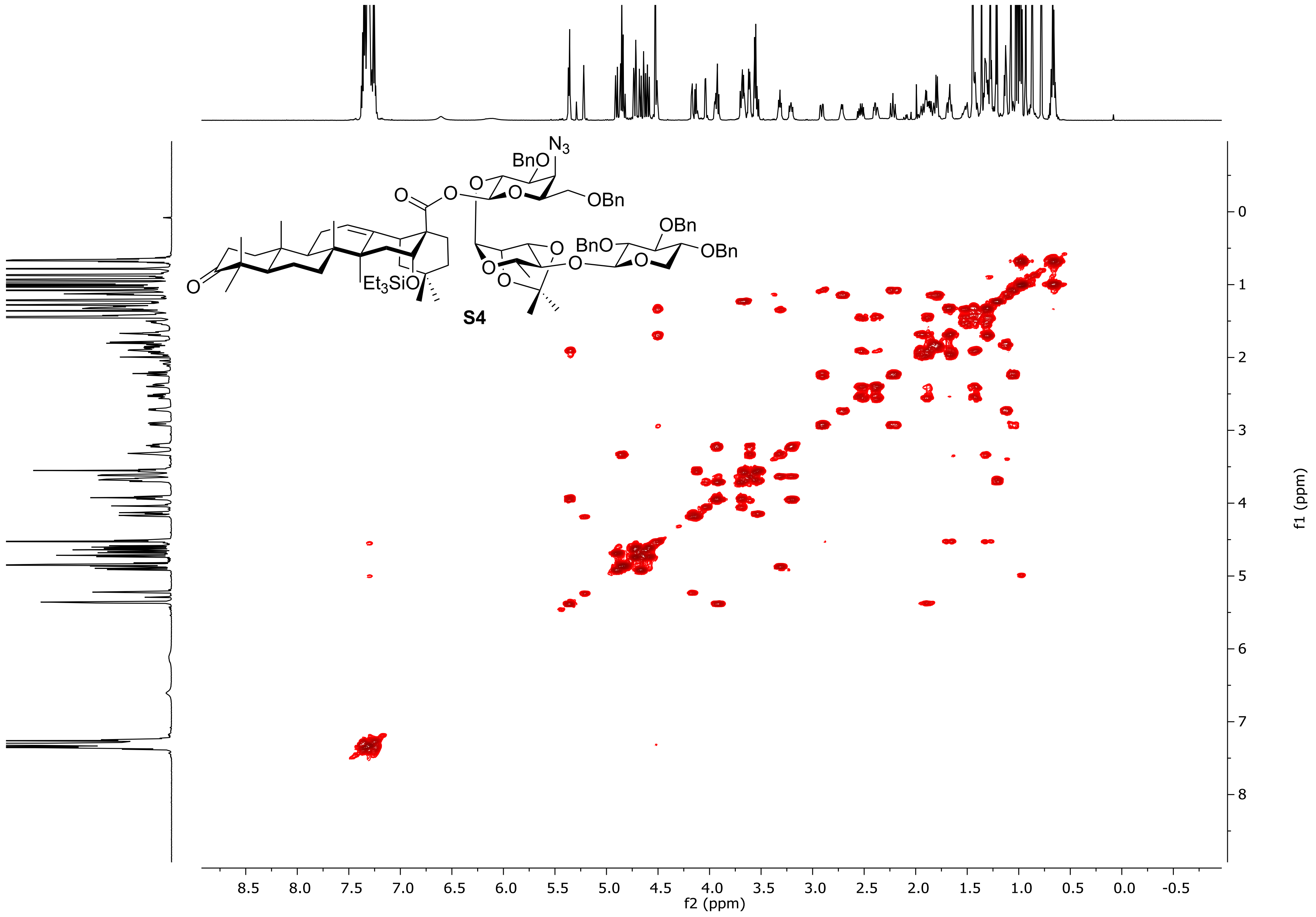

S4 ^1H-^{13}C HSQC (600 MHz, $CDCl_3$)

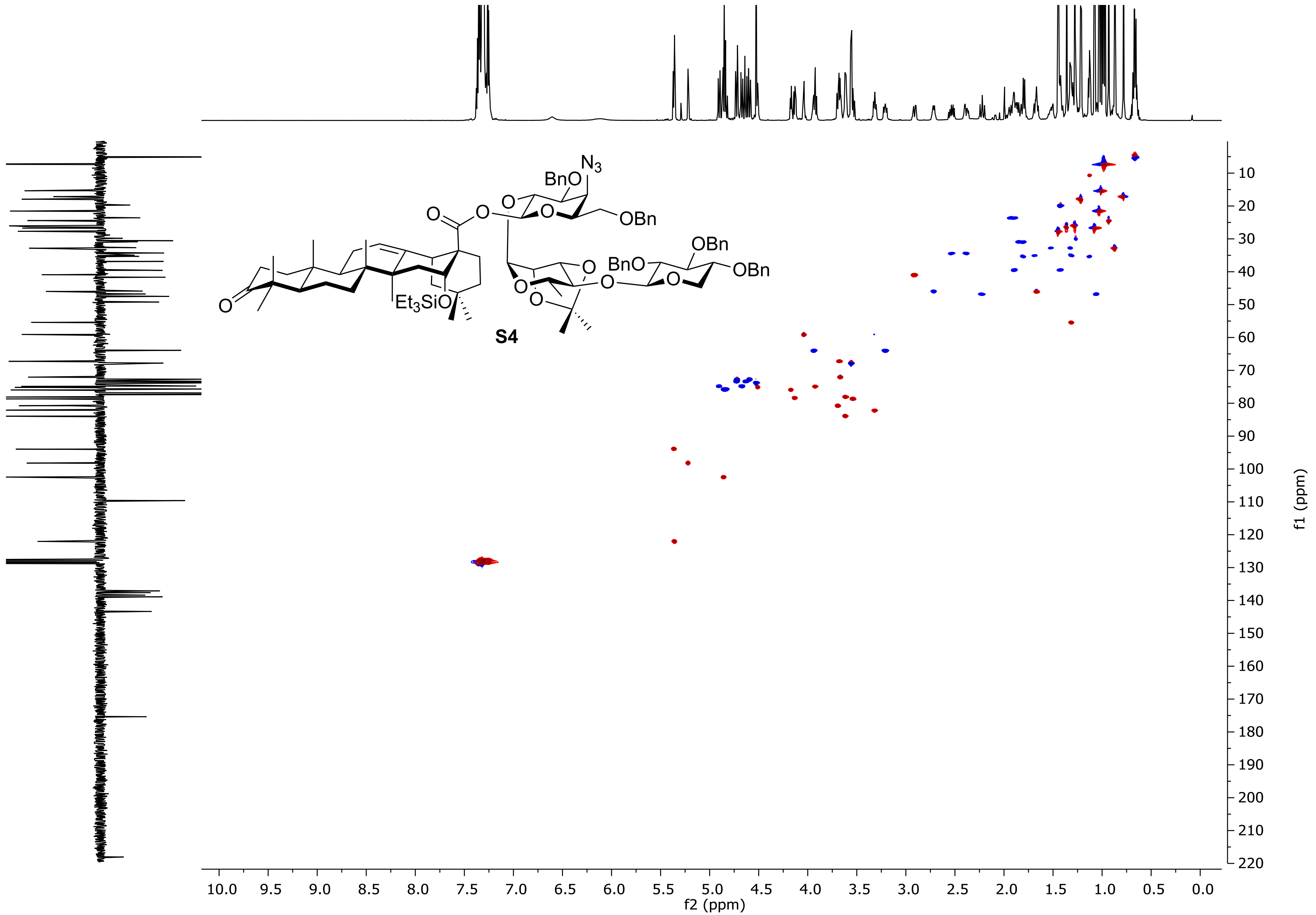

10 ^{1}H-NMR (600 MHz, $CDCl_3$)

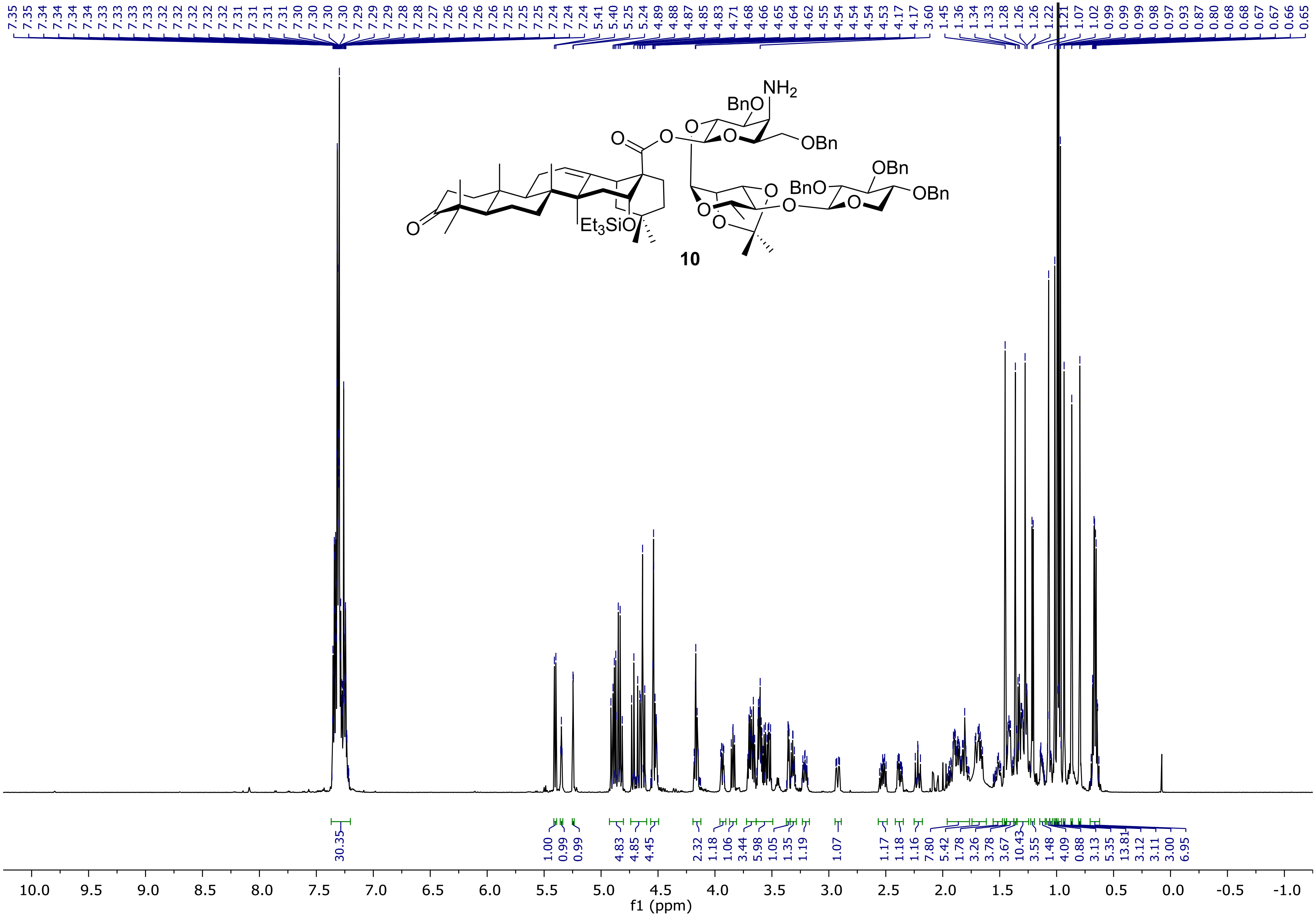

10 APT ^{13}C-NMR (151 MHz, $CDCl_3$)

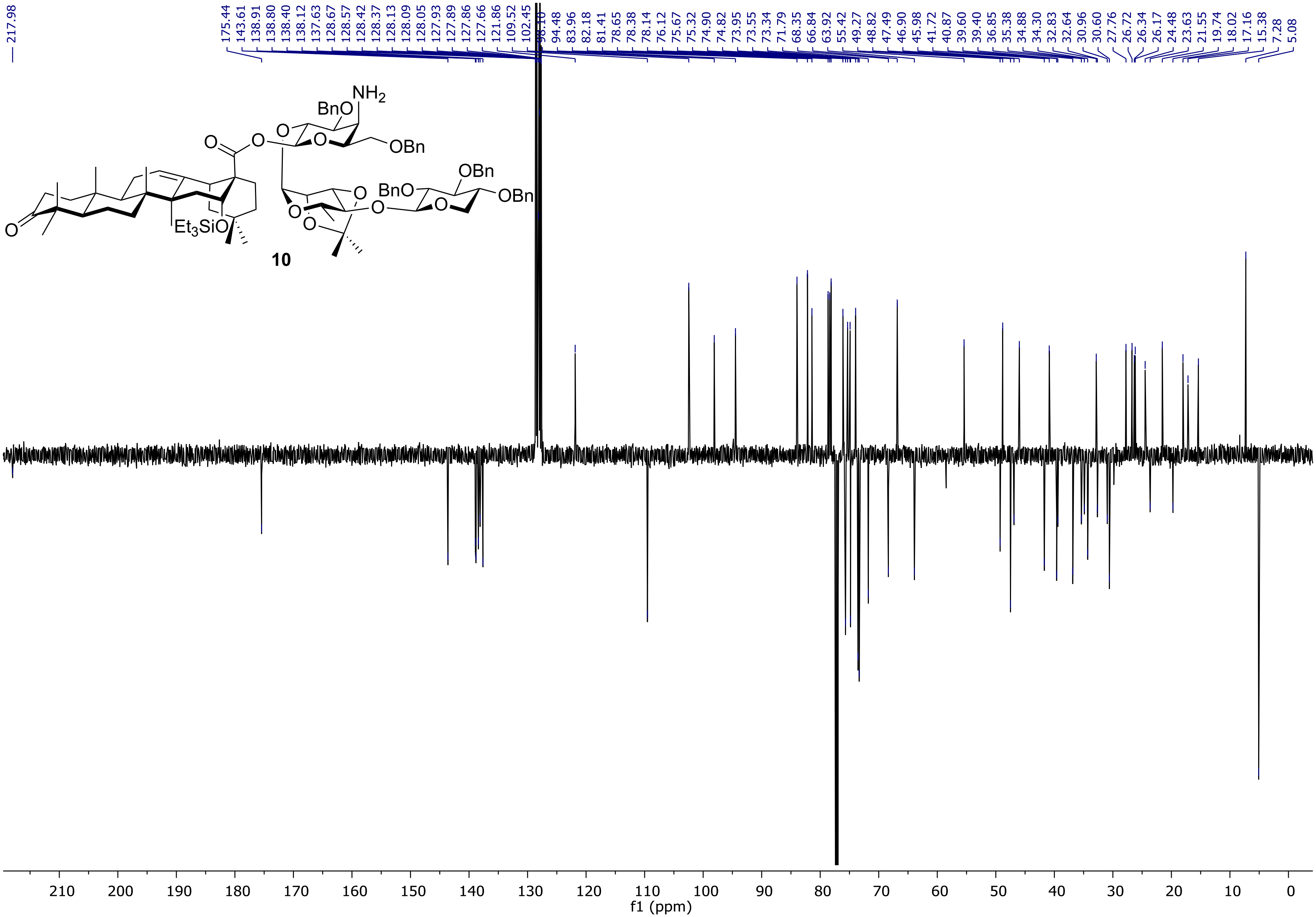

10 ^{1}H-^{1}H COSY (600 MHz, $CDCl_3$)

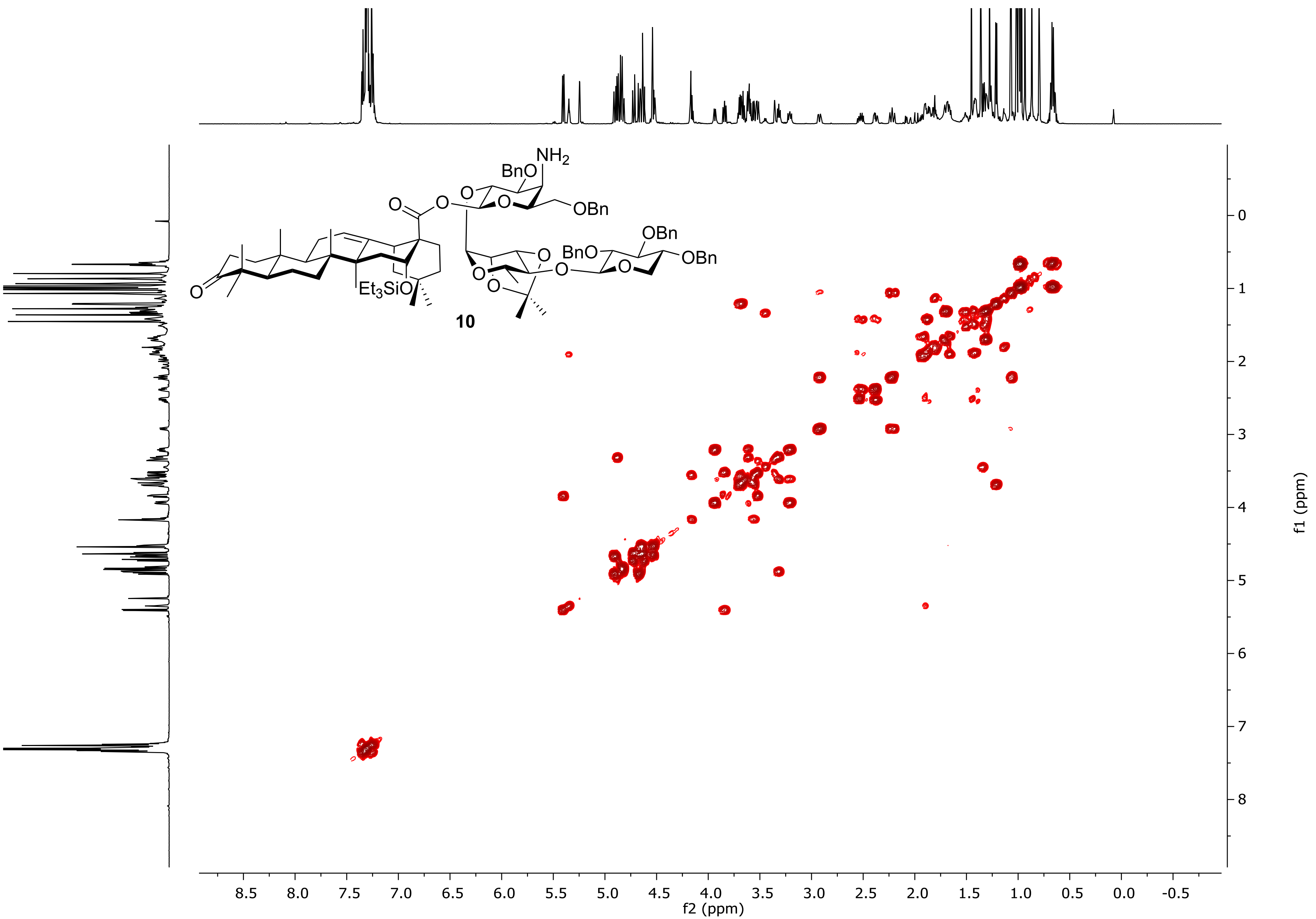

10 ^{1}H-^{13}C HSQC (600 MHz, $CDCl_3$)

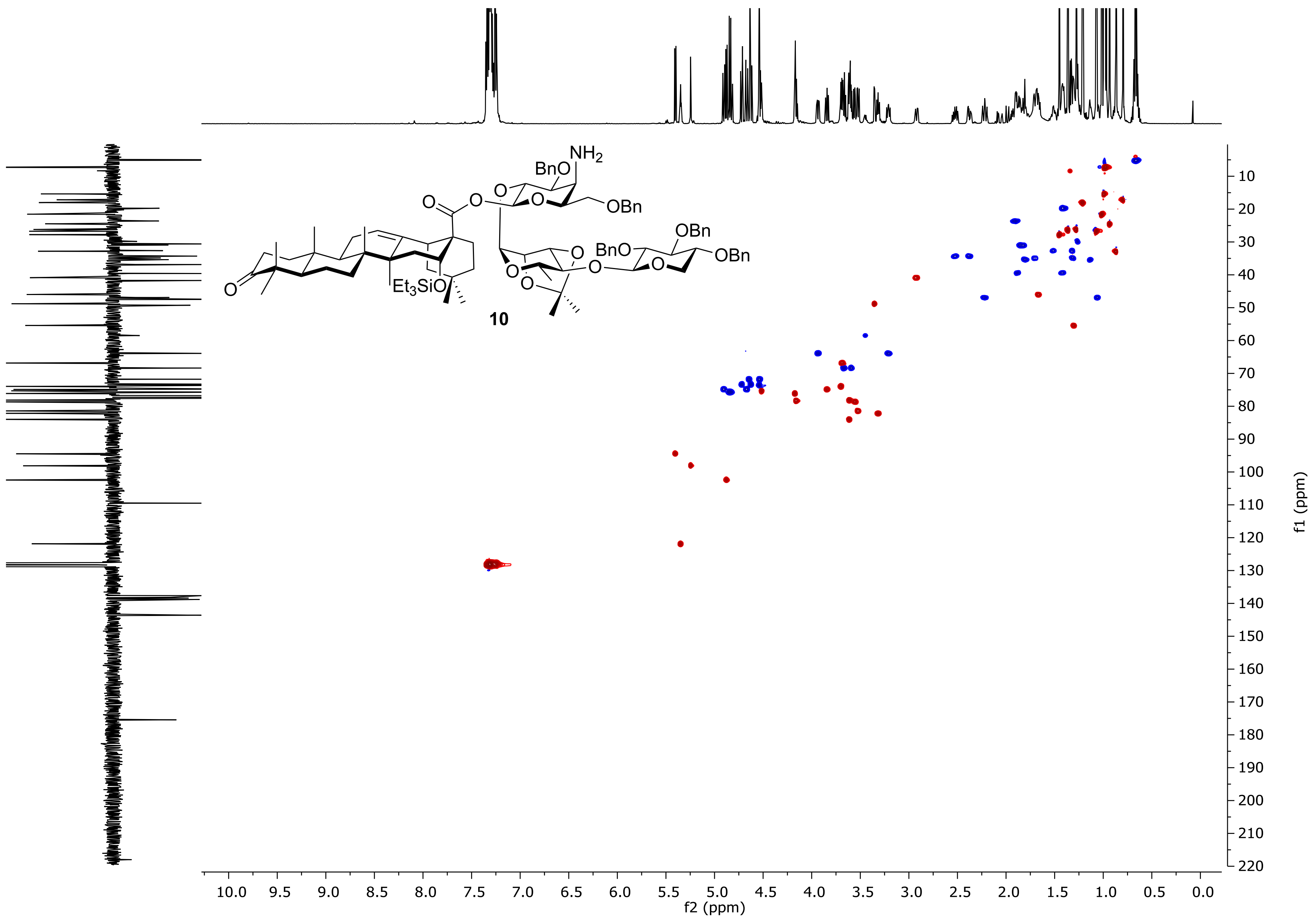

S5 ^{1}H-NMR (400 MHz, $CDCl_3$)

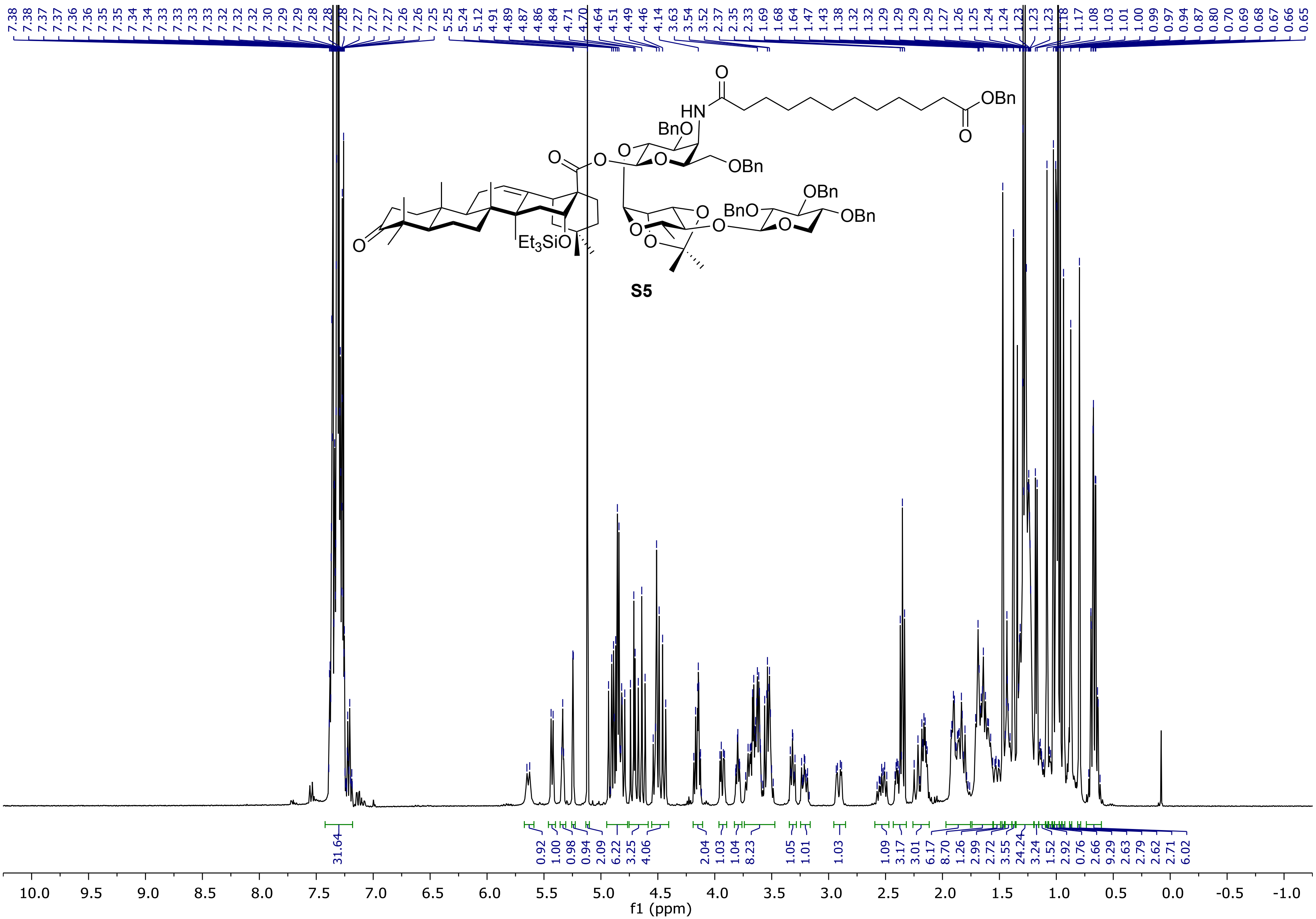

S5 APT ^{13}C-NMR (101 MHz, $CDCl_3$)

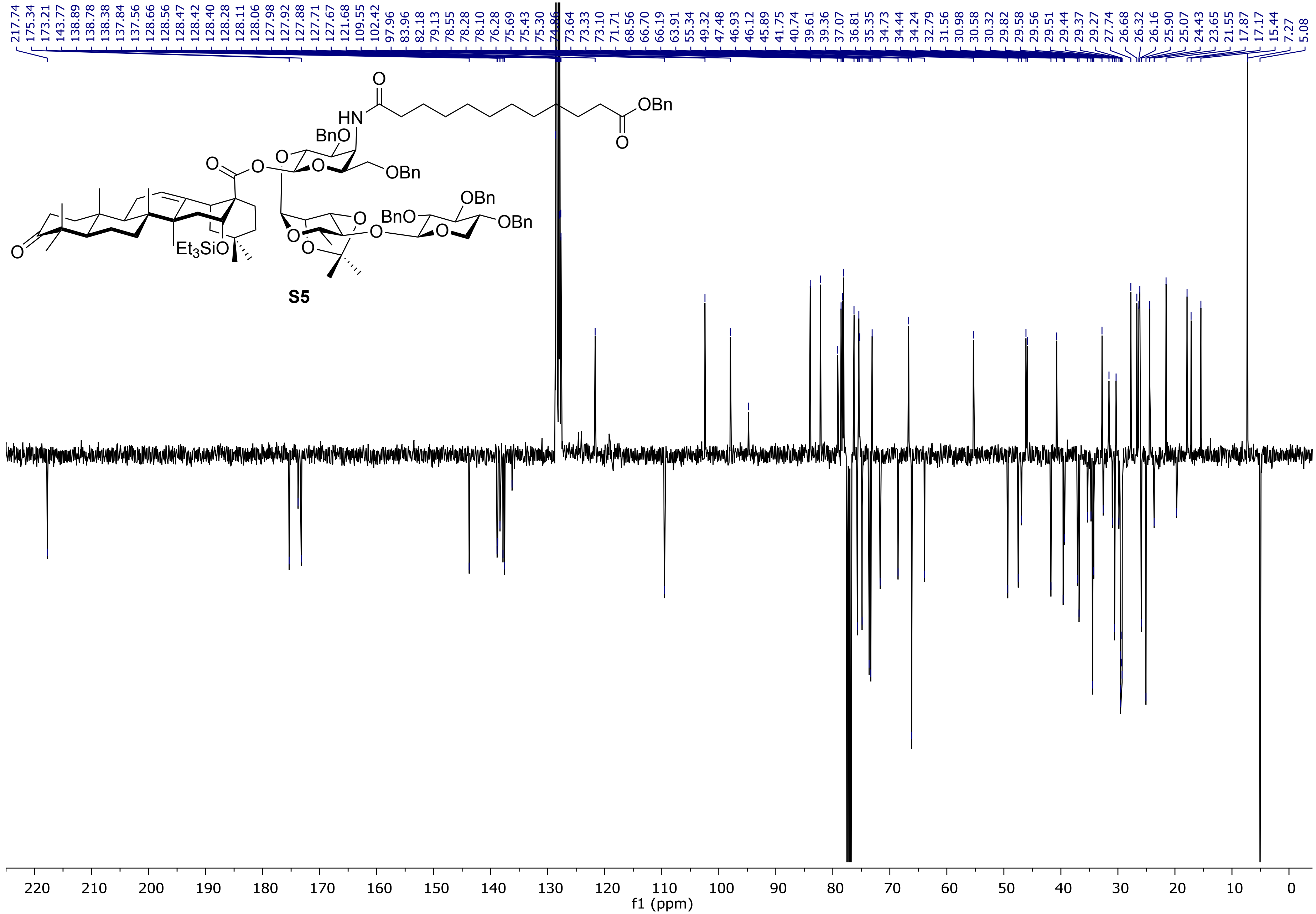

S5 ^1H-^1H COSY (400 MHz, $CDCl_3$)

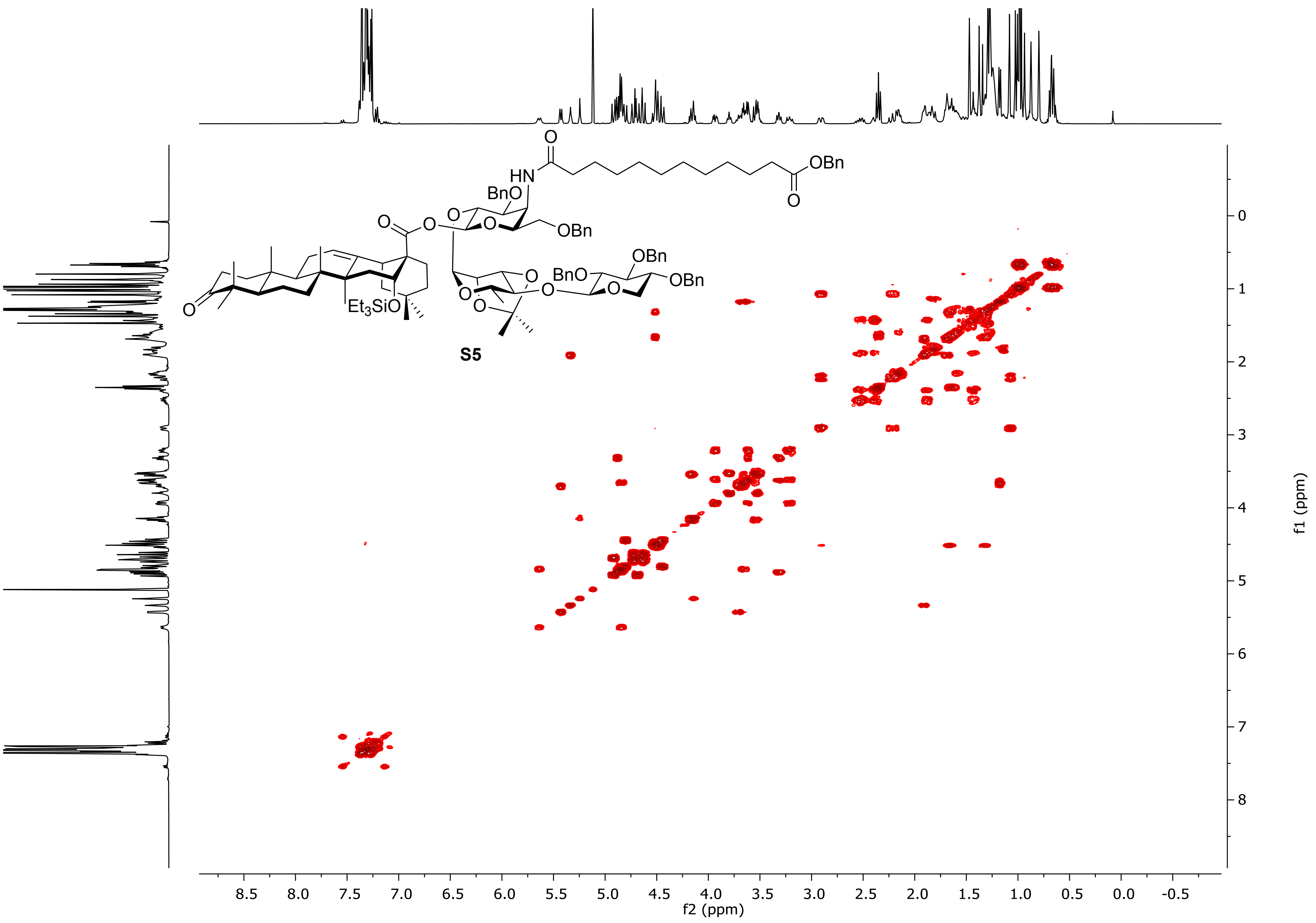

S5 ^{1}H-^{13}C HSQC (400 MHz, $CDCl_3$)

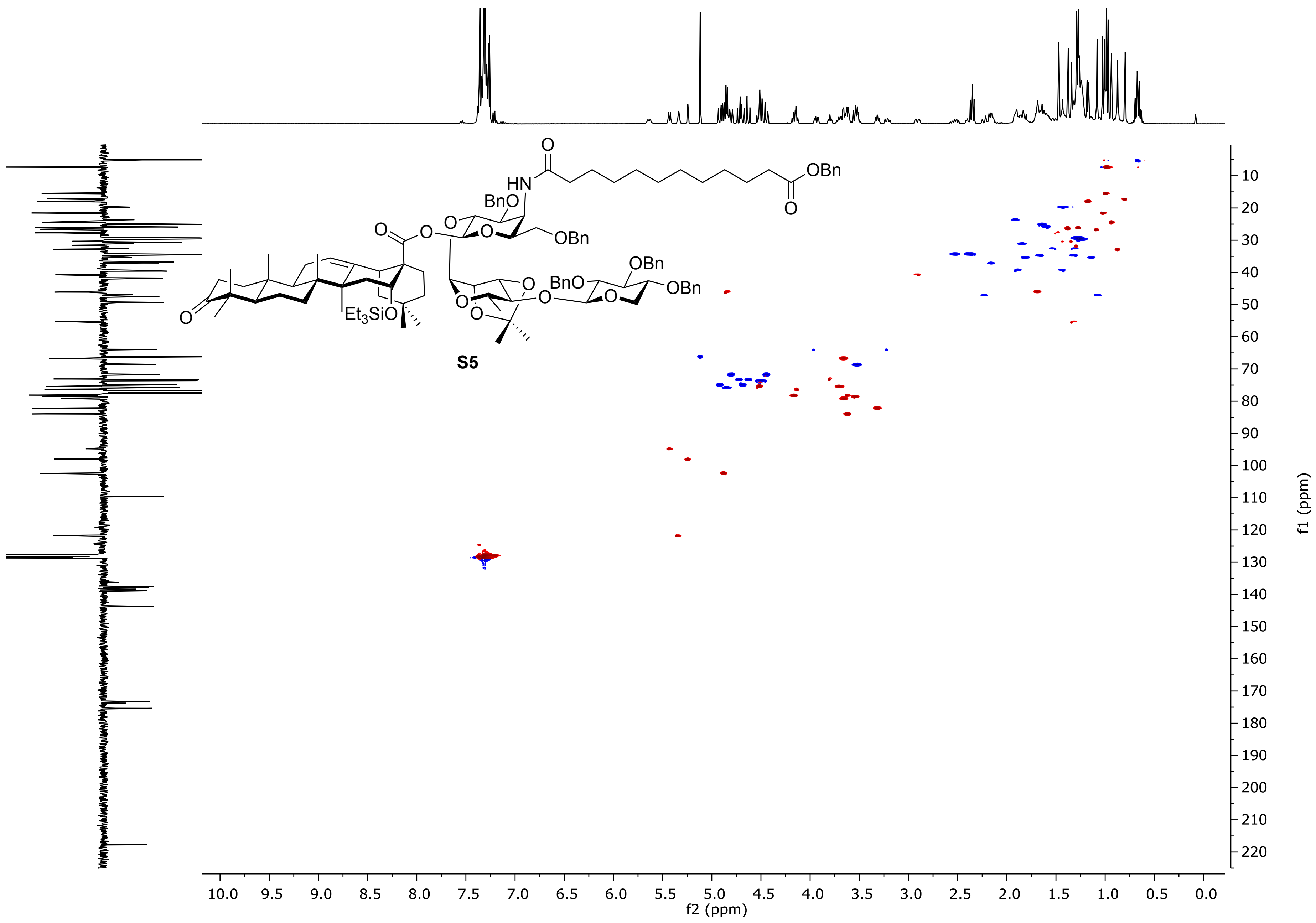

5 ^{1}H-NMR (400 MHz, methanol-d_4)

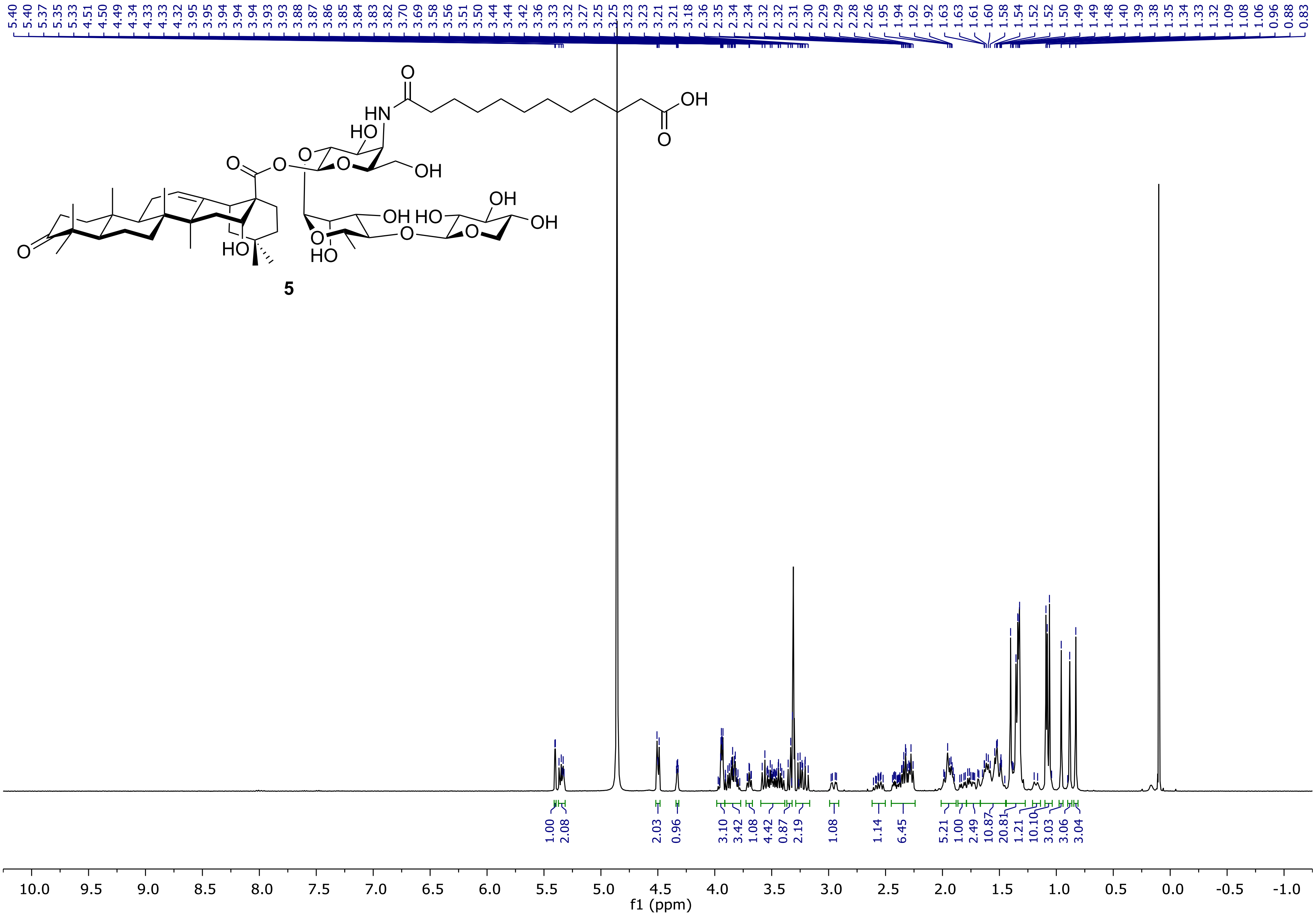

5 APT ^{13}C-NMR (101 MHz, methanol-d_4)

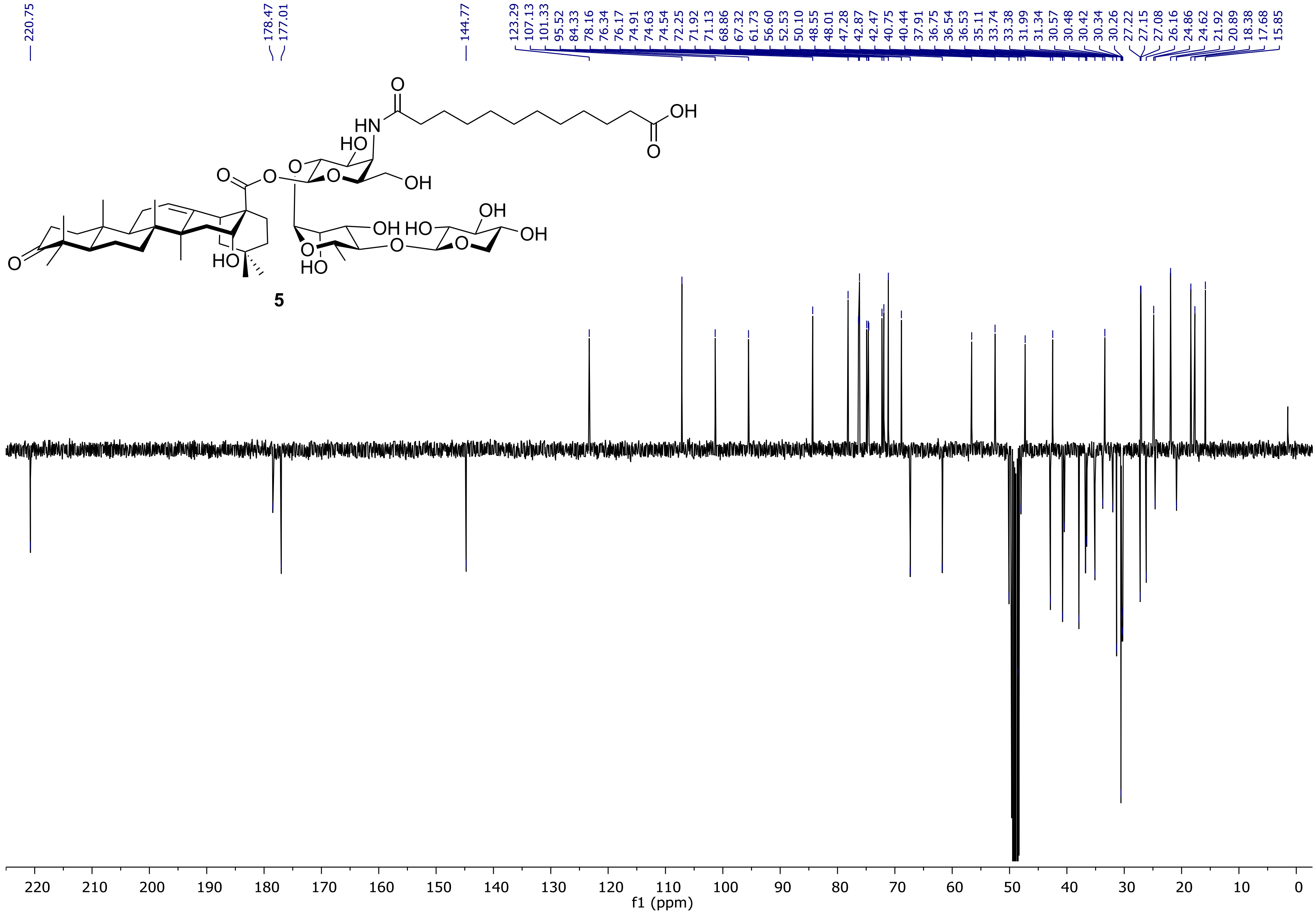

5 ^{1}H-^{1}H COSY (400 MHz, methanol-d_4)

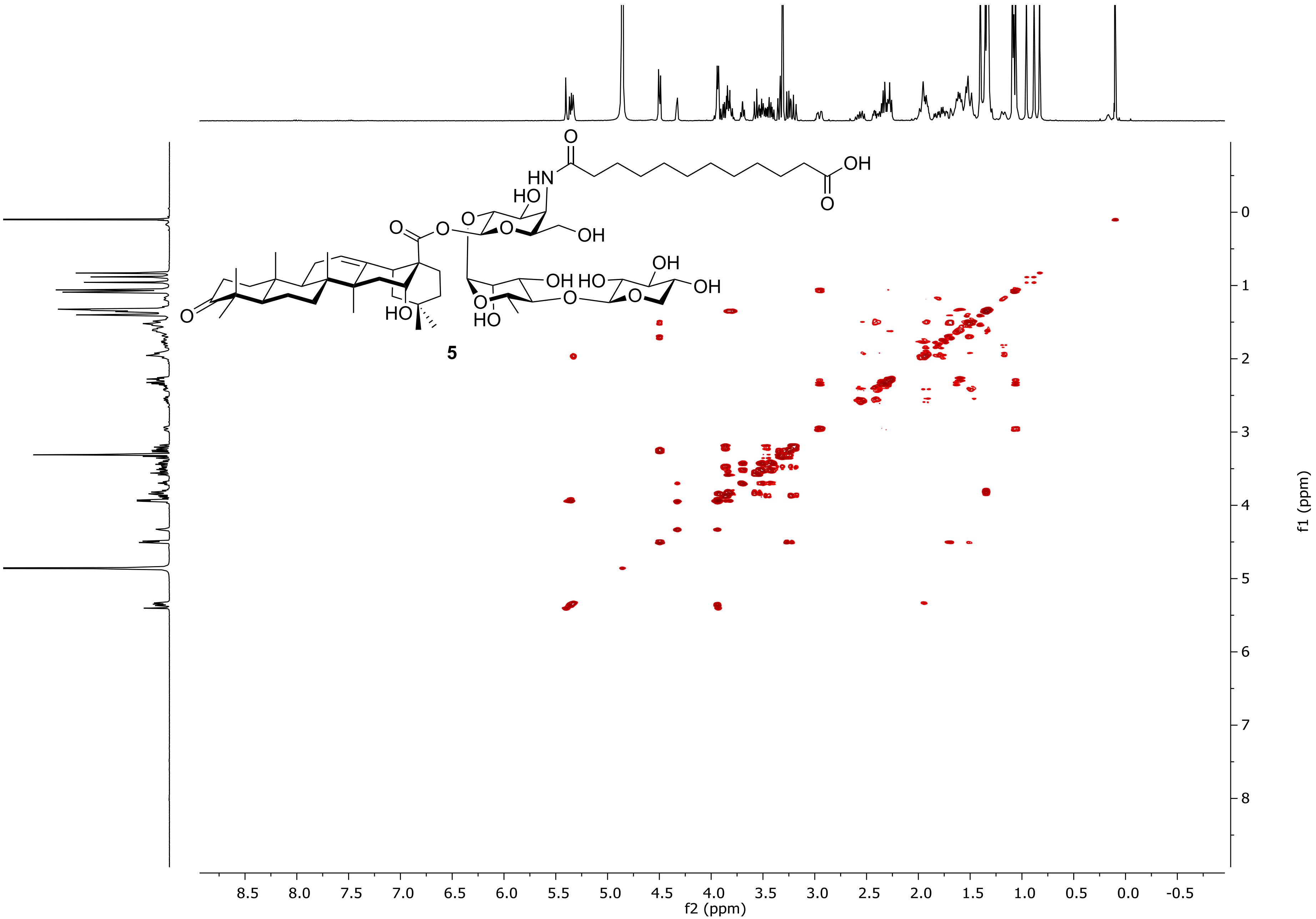

5 ^{1}H-^{13}C HSQC (400 MHz, methanol-d_4)

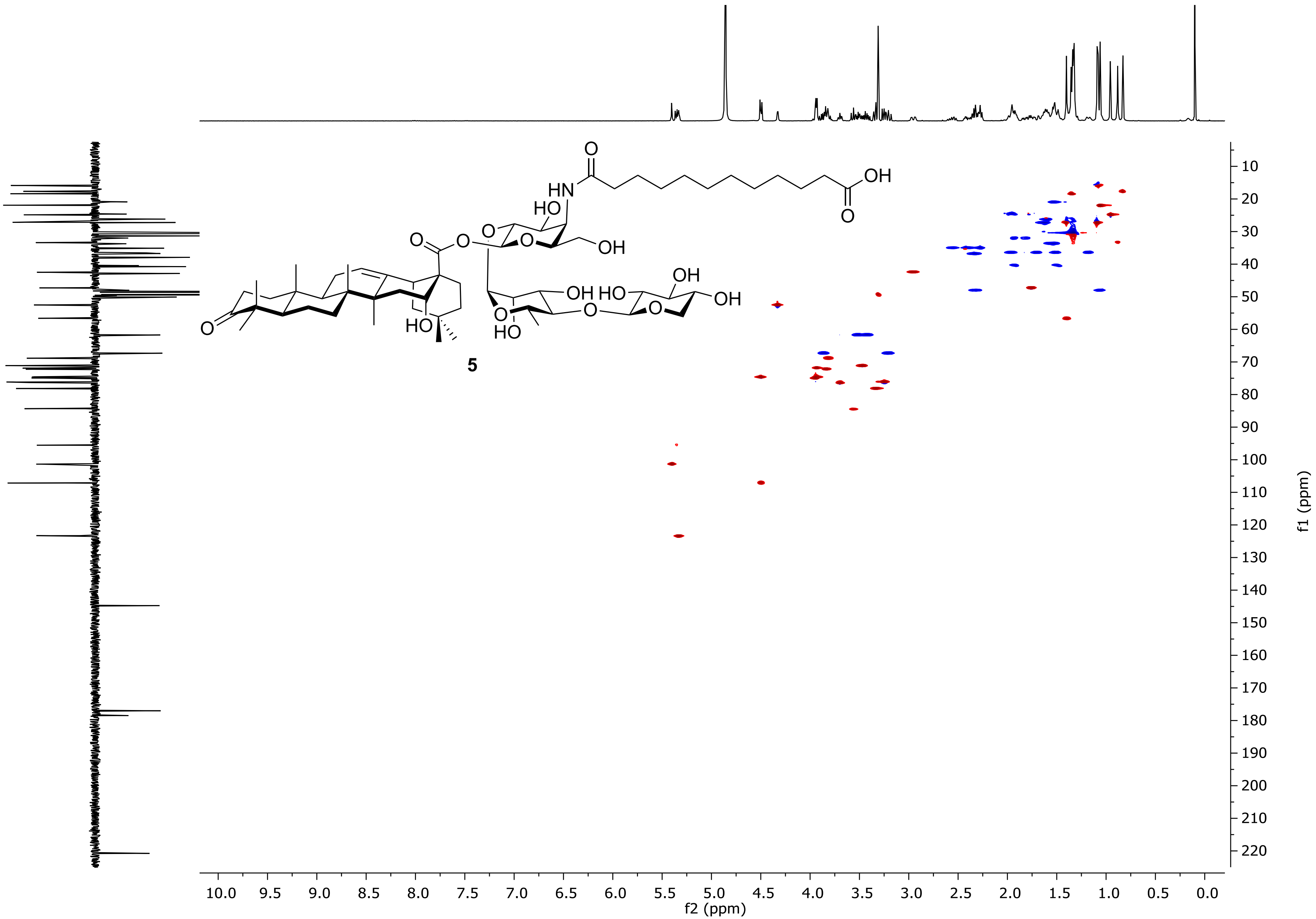

6 ^{1}H-NMR (400 MHz, methanol-d_4)

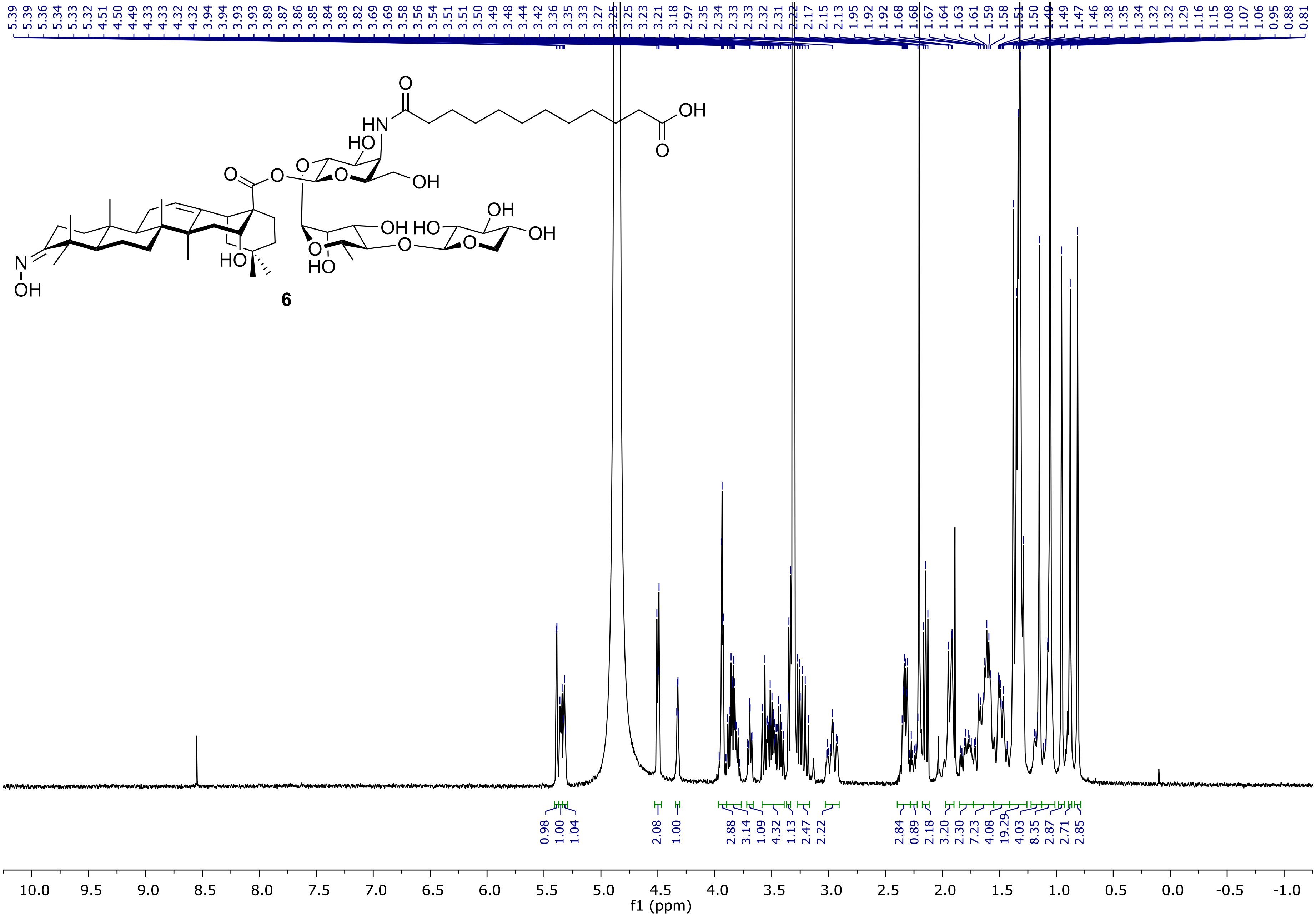

6 APT ^{13}C-NMR (101 MHz, methanol-d_4)

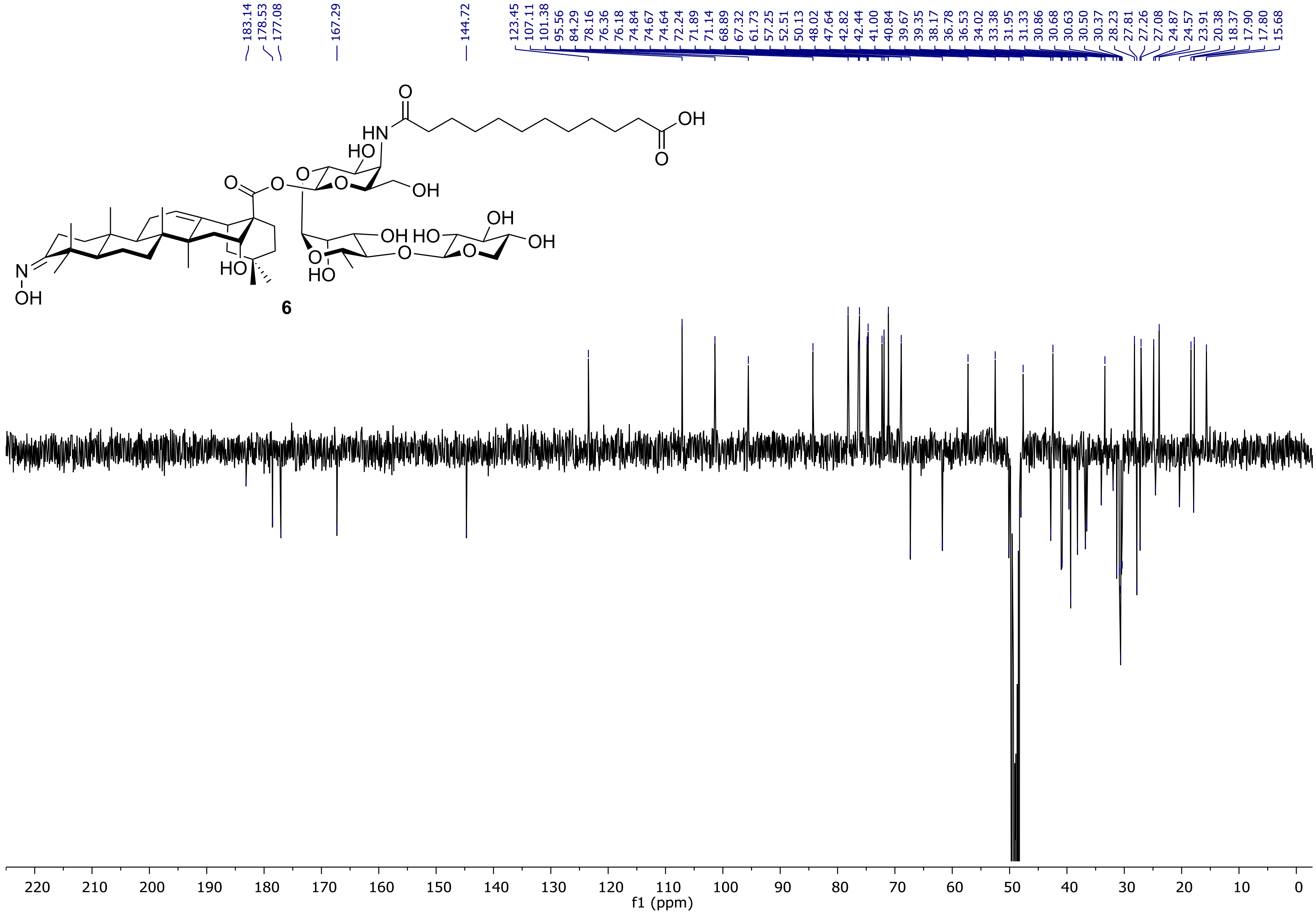

6 ^{1}H-^{1}H COSY (400 MHz, methanol-d_4)

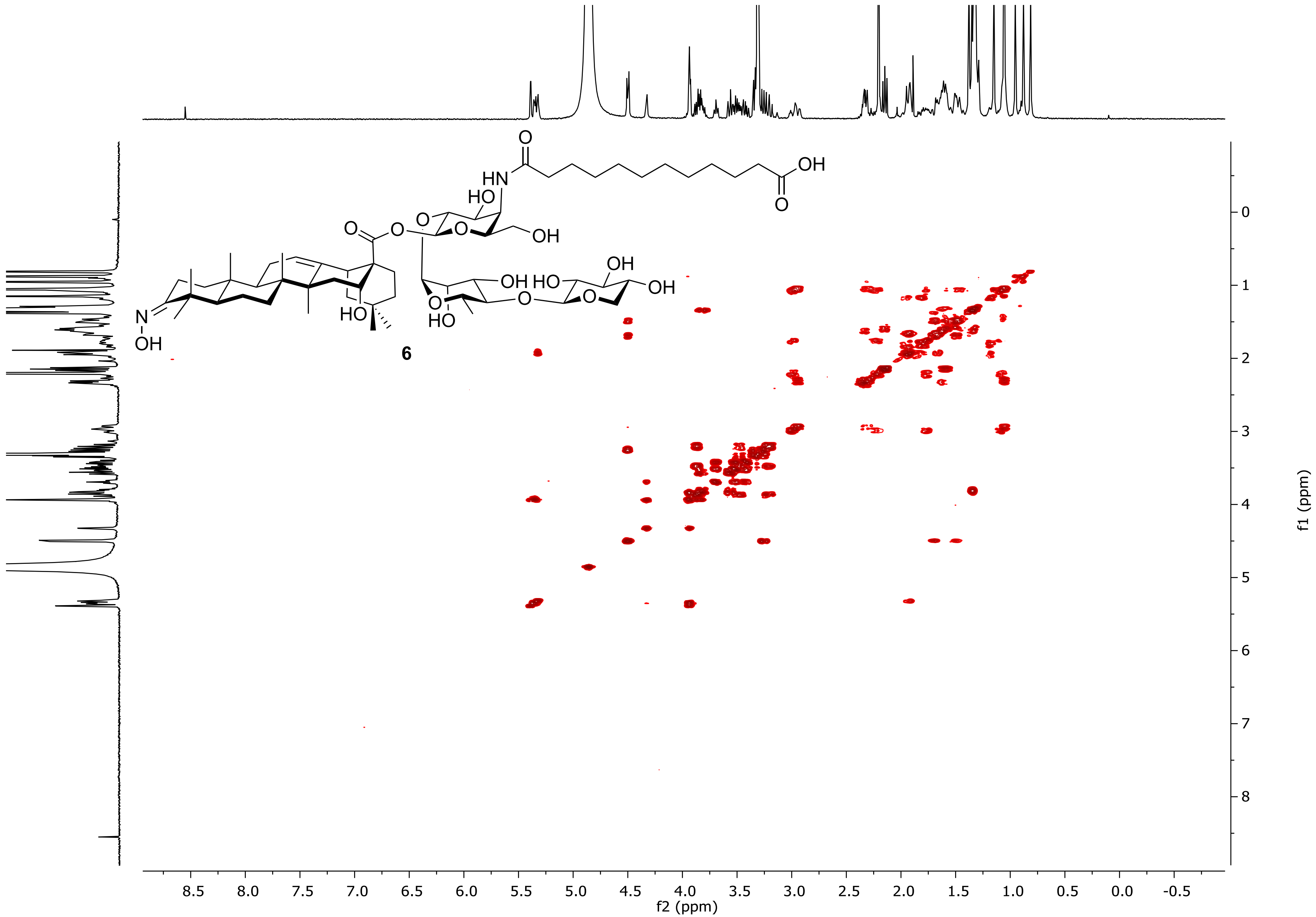

6 ^{1}H-^{13}C HSQC (400 MHz, methanol-d_4)

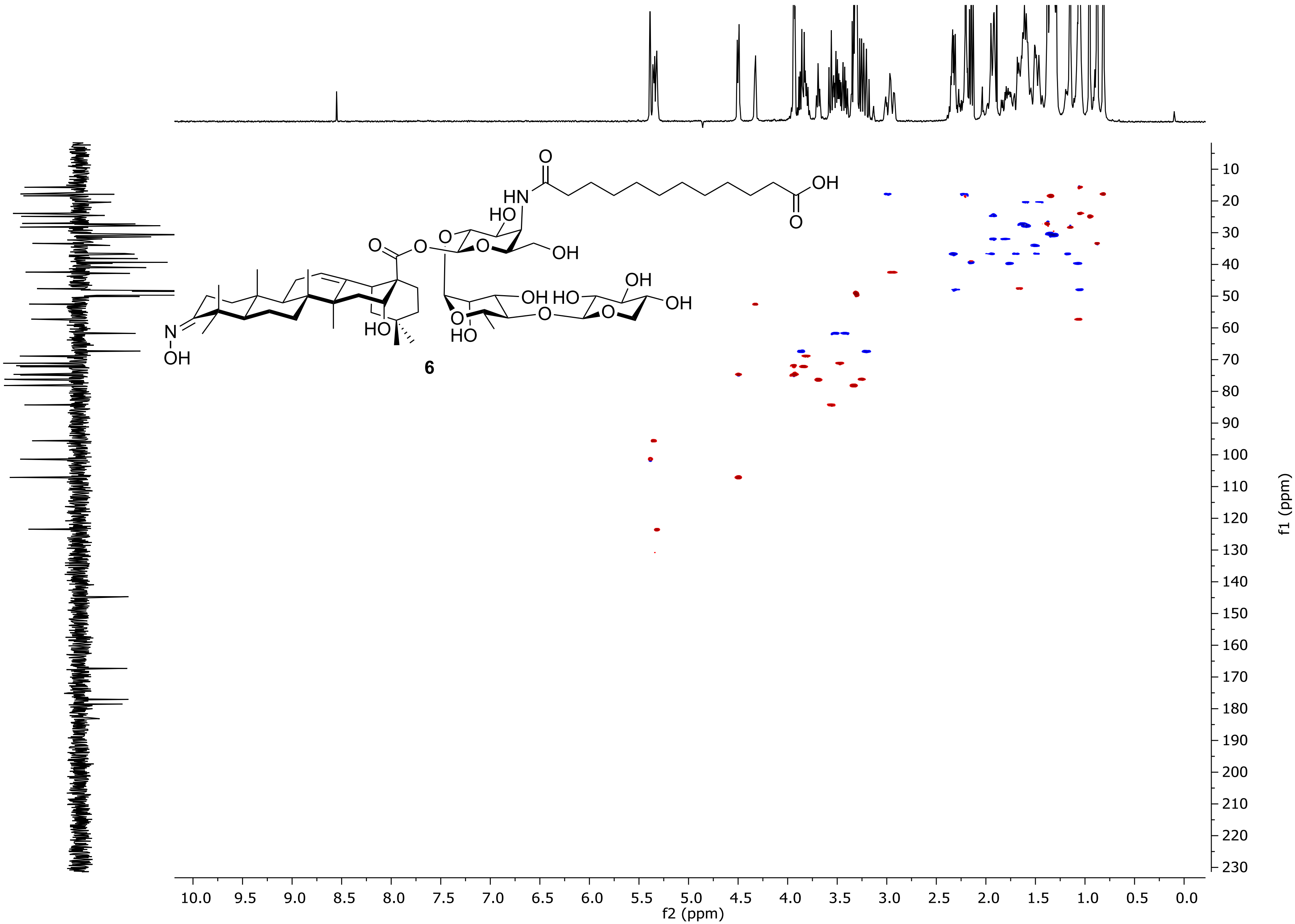

VI. SUPPLEMENTARY MATERIAL REFERENCES

[1] Wang H, Yu F, Peng Y, Wang Q, Han X, Xu R, Zhou X, Wan C, Fan Z, Jiao P, Zhang Y, Zhang L, Zhou D, Xiao S. *Eur. J. Med. Chem.* (2015) 102:594-599.

[2] Ghirardello M, Ruiz-de-Angulo A, Sacristan N, Barriales D, Jiménez-Barbero J, Poveda A, Corzana F, Anguita J, Fernández-Tejada A. *Chem. Commun.* (2020) 56:719–722.

[3] Fernández-Tejada A, Chea EK, George C, Pillarsetty N, Gardner JR, Livingston PO, Ragupathi G, Lewis JS, Tan DS, Gin DY. *Nature Chem*. (2014) 6:635–643.
